# Supplementary material for: Effective engineering of a ketoreductase for the biocatalytic synthesis of an ipatasertib precursor
Source: Commun Chem. 2024 Feb 28;7:46. doi: 10.1038/s42004-024-01130-5 (PMC10902378; doi:10.1038/s42004-024-01130-5)
Supplement: Supplementary file 2 — Supplementary Information [file 42004_2024_1130_MOESM2_ESM.pdf]

Supplementary Information

Effective Engineering of a Ketoreductase for the Biocatalytic Synthesis of an Ipatasertib Precursor

Sumire Honda Malca[1], Nadine Duss[1], Jasmin Meierhofer[1], David Patsch[1], Michael Niklaus[1], Stefanie Reiter[1], Steven Paul Hanlon[2], Dennis Wetzl[2], Bernd Kuhn[3], Hans Iding[2], and Rebecca Buller[1]✉

1. Competence Center for Biocatalysis, Institute of Chemistry and Biotechnology, Zurich University of Applied Sciences, Einsiedlerstrasse 31, 8820, Waedenswil, Switzerland
2. Process Chemistry & Catalysis, F. Hoffmann-La Roche Ltd., Grenzacherstrasse 124, 4070 Basel, Switzerland
3. Pharmaceutical Research and Early Development, F. Hoffmann-La Roche Ltd., Grenzacherstrasse 124, 4070 Basel, Switzerland

✉ email: rebecca.buller@zhaw.ch

Table of Contents

Table of Contents 2
1. Supplementary Methods 3
  1.1. Materials 3
  1.2. Synthesis of substrates and products 3
  1.3. Cloning and transformation 4
  1.4. KRED expression for screening 4
  1.5. KRED expression for biocatalytic reactions, lysate lyophilization or protein purification 5
  1.6. Lysate treatment prior to UV assay 5
  1.7. Sequencing and activity data analysis 5
  1.8. Small-scale biocatalytic reactions 5
  1.9. Preparative-scale biocatalytic reactions 6
  1.10. HPLC-UV analysis of substrate and products 6
  1.11. Determination of kinetic constants 7
  1.12. Illustrations 7
2. Supplementary Figures 8
3. Supplementary Tables 27
4. *Ssal*-KRED sequences 53
5. Supplementary References 56

1. Supplementary Methods

1.1. Materials

Media, buffer salts, reagents and general chemicals were purchased from Merck/Sigma-Aldrich (Darmstadt, Germany), Carl Roth (Karlsruhe, Germany) or Roche Diagnostics (Rotkreuz, Switzerland). Q5 High-Fidelity DNA polymerase, T5 exonuclease, dNTPs, restriction enzymes and T4 DNA ligase were acquired from New England Biolabs (Ipswich, MA, USA). Oligonucleotides were synthesized by Microsynth (Balgach, Switzerland). General molecular cloning kits, In-Fusion HD Cloning Kit and BCA Protein Assay Kit were sourced from Macherey-Nagel (Dueren, Germany), Takara Bio (Shiga, Japan) and ThermoFisher Scientific (Rockford, IL, USA), respectively. NAD(P)$^+$-dependent glucose dehydrogenase GDH-105 was obtained as a lyophilized enzyme from Codexis, Inc. (San Francisco, CA, USA).

1.2. Synthesis of substrates and products

Starting material **1a** ((*R*)-ketone) as well as diastereomeric alcohols **2a** ((*R*,*R*)-*trans* alcohol; target product) and **2b** ((*R*,*S*)-*cis* alcohol (**Supplementary Fig. 1**) were synthesized internally and analyzed by HPLC-UV/MS and ^{1}H NMR. Diastereomers **2c** ((*S*,*R*)-*cis* alcohol) and **2d** ((*S*,*S*)-*trans* alcohol) were not synthesized but detected in trace amounts in preparative-scale KRED-catalyzed reactions and identified using chiral HPLC analysis. HPLC methods are described in **Supplementary Method 1.10**. NMR spectra were recorded on a Bruker Avance III 600 MHz spectrometer equipped with a 5 mm TCI, Z-gradient CryoProbe using TopSpin (4.x) as software. ACD/NMR Workbook 2019 was utilized for the analysis of NMR data.

Synthesis of 1a: (*R*)-4-(5-methyl-7-oxo-6,7-dihydro-5H-cyclopenta[d]pyrimidin-4-yl)piperazine-1-carboxylic acid *tert*-butyl ester was synthesized using triple jacket reactors, a Huber chiller, RX10 or Optimax reactors, an overhead stirrer with impeller, a reflux condenser, an argon/nitrogen inlet, a peristaltic pump (Ismatec), a membrane pump (KNF Simdos 02) in loop with balance (Mettler), a universal control box (UCB) and a pH electrode (Mettler/Knick Portamess 911). NMR spectra were recorded on a Bruker Avance III 600 MHz spectrometer equipped with a 5 mm TCI, Z-gradient CryoProbe. 25 g 4-[6-bromo-5-[(2*R*)-1-cyanopropan-2-yl]pyrimidin-4-yl]piperazine-1-carboxylic acid *tert*-butyl ester was dissolved in 75 mL toluene. At 7.5 °C 35.3 mL 2-PrMgCl (2 M in THF) was added within 4.5 h. The reaction mixture was directly added on a mixture of 100 mL 2-methyltetrahydrofuran (MeTHF) and 88 mL water at 2.5 °C. The pH was kept between 1.5 to 2.5 by simultaneous addition of aqueous sodium bisulfate (40 % w/w $NaHSO_4$ in water). After addition, the pH was adjusted to 4.5 to 5.5 with 4.5 mL 2 M aqueous sodium hydroxide (NaOH), followed by heating to 20 °C and stirring of the reddish-brown biphasic mixture for another 60 minutes at this temperature and pH. The phases were separated, and the lower aqueous phase was discarded. The organic phase was extracted with 25 mL water. The organic phase was concentrated by distillation to a volume of 37.5 mL. MeTHF (20 mL) was added to the brownish slurry at 45 °C. To finalize the crystallization, a mixture of 24 mL *n*-heptane / 36 mL methyl-*tert*-butyl ether (MTBE) was added within 90 minutes at 45 °C. The slurry was cooled to 0 °C and the crude product was isolated by filtration. The crude product was slurried in MeTHF at 45 °C for 2 h and a mixture of *n*-heptane/MTBE was added. The mixture was cooled to 0°C and the product was isolated by filtration and washed with a mixture of *n*-heptane/MTBE. The product was dried at 50 °C and reduced pressure until constant weight was attained. The pure product was isolated as a pale yellow to off-white powder. An average yield of 67 %, corresponding to 13.8 g, and an average purity of 99.4 % (w/w) **1a** were obtained. **1a** ESI-MS (positive): 333.40 (M+H$^+$; $C_{17}H_{24}N_4O_3$ + H$^+$). **1a** ^{1}H NMR (600 MHz, DMSO-d_6) δ ppm 8.63 (s, 1 H), 3.62 - 3.88 (m, 5 H), 3.39 - 3.56 (m, 4 H), 2.86 - 2.95 (m, 1 H), 2.20 - 2.29 (m, 1 H), 1.43 (s, 9 H), 1.16 - 1.23 (m, 3 H), 1.04 (d, *J*=6.0 Hz, 1 H).

Synthesis of 2a: 4-[(5*R*,7*R*)-7-hydroxy-5-methyl-6,7-dihydro-5H-cyclopenta[d]pyrimidin-4-yl]piperazine-1-carboxylic acid *tert*-butyl ester was synthesized from **1a** enzymatically (see **Supplementary Method 1.9**). For NMR spectra see **Supplementary Data 1**.

Synthesis of 2b: 4-[(5*R*,7*S*)-7-hydroxy-5-methyl-6,7-dihydro-5H-cyclopenta[d]pyrimidin-4-yl]piperazine-1-carboxylic acid *tert*-butyl ester was synthesized enzymatically using a 100 mL pH-stat with propeller-stirrer system (Metrohm). The reactor contained **1a** (3 g, 9.03 mmol, 1 eq), 21 mL 0.1 M MES buffer pH 5.8, 8 mL iPrOH and NADP$^+$ (15 mg, 0.0025 eq, s/c = 200). After stirring for 5 min at 40 °C, the reaction was started by addition of KRED-P2D03 lyophilized lysate (Codexis, Inc., 50 mg, s/e = 20). The pH decreased to 5.64 and was re-adjusted to 5.8. The reaction proceeded for 120 h at 40 °C. After this time, 0.1 mL reaction mixture was quenched with 0.9 mL HPLC-grade methanol, vortexed, centrifuged (3,220 g, 23 °C, 5 min) and filtered for achiral HPLC-UV (achiral IPC) analysis. The yield achieved was 96.1 area % *cis*-product (0.5 area % *trans*-product, 3.2 area % substrate). The reaction mixture was transferred for iPrOH (15 %) / acetone (1.3 %) evaporation (50 °C, 200 – 90 mbar). The crude product was filtered off (paper filter), washed with water and n-heptane and dried. A beige powder (2.8 g) was isolated with a HPLC purity of 96.2 area % *cis*-product (0.4 area % *trans*-product, 3.4 area % substrate). **2b** ESI-MS (positive): 335.21 (M+H$^+$; $C_{17}H_{26}N_4O_3$ + H$^+$). **2b** ^{1}H NMR (600 MHz, $CDCl_3$) δ ppm 1.23 - 1.34 (m, 3 H) 1.46 - 1.51 (m, 9 H) 1.55 - 1.65 (m, 1 H) 2.73 (dt, *J*=13.5, 7.9 Hz, 1 H) 3.19 - 3.35 (m, 1 H) 3.39 - 3.73 (m, 8 H) 3.73 - 3.92(m, 1 H) 5.01 (dd, *J*=8.2, 6.0 Hz, 1 H) 8.49 - 8.63 (m, 1 H).

1.3. Cloning and transformation

Supplemented in-house KRED collection. The panel of 63 literature-based and putative KRED candidates is presented in Table S1. Our initial in-house collection of 51 KREDs, previously shown to be active towards 2 - 5 industrially-relevant substrates,[1] was expanded with 12 additional genes. The selection of the newly introduced genes follows the same rationale used for the first collection.[1] The genes were codon-optimized for *E. coli*, synthesized and cloned into pET22b(+) by Twist Bioscience (San Francisco, CA, USA).

***Ssal*-KRED libraries and variants.** The mutational scanning library L1 was generated by Twist Bioscience and received as a cloned library in pET22b(+). Except for L8, libraries L2 - L13 were constructed by PCR amplification from regions close to an *Age*I or *Xma*I site, 52 or 180 bp upstream from the *Ssal*-KRED gene start codon to allow only one Sanger sequencing read (~ 1 kb) per variant. Targeted sites were mutated using ratio-tuned NDT/VHG/TGG primers (22c-trick technique),[2] NNK primers or other specific (non-degenerate or manually-mixed degenerate) mutagenic primers (**Supplementary Table 21**) on the wild type or defined variants as templates. The PCR-amplified fragments (1 – 3) were assembled by overlap extension PCR with general flanking primers (**Supplementary Table 21**) and cloned into *Age*I/*Bam*HI-, *Xma*I/*Bam*HI-linearized or inverse PCR-amplified backbone by In-Fusion cloning following the manufacturer's recommendations. Alternatively, T5-exonuclease-dependent assembly (TEDA)[3] was used for cloning of small libraries or single constructs. The 5-site CSM library L7 was prepared by Twist Bioscience as a pooled gene fragment library in a 2-step PCR-based approach using 20 and 160,000 mutagenic oligonucleotides on template pET22b(+)_*Ssal*-KRED_F97W to generate fragment 1 (L174X) and fragment 2 (A238X_L241X_M242X_Q245X), respectively. The library was PCR-amplified in-house with the same general flanking primers and inserted into the vector backbone by In-Fusion cloning. Focused combinatorial library L8 and hit combinations were generated using the MEGAWHOP PCR technique[4] with mutagenic primers on the wild-type or defined variants, followed by amplification of the megaprimer on selected plasmid templates and *Dpn*I digestion. Selected *Ssal*-KRED hit variants (M1 – M6) and the wild type were subcloned into pET28b(+) for the insertion of an *N*-terminal 6xHis-tag. Genes were cloned between *Nde*I and *Xho*I sites using a standard restriction digestion and ligation protocol.

Chemically ultracompetent *E. coli* Stellar cells (Takara Bio) and chemically competent *E. coli* NEB10-beta cells (New England Biolabs) were employed as cloning strains for transformation of large and small-sized libraries, respectively. *E. coli* BL21(DE3) was used as expression strain for all libraries. Isolated pooled library plasmids, plasmids from single transformants or *E. coli* pre-cultures (LB containing 200 mg L^{-1} Ampicillin) in a 96-well plate format were sent for Sanger sequencing (Microsynth, Balgach, Switzerland).

1.4. KRED expression for screening

Recombinant strains were cultivated in a 96-well plate format (Duetz system, Kuehner shaker, 5 cm shaking diameter) for sequencing and screening. All plates contained strains with empty vector (negative control) as well as the immediate parental enzyme in triplicates as reference for fold-improvement over the parent (FIOP) estimation.

Mutational scanning library L1. This library was screened on a second site. Precultures were started by inoculation of fresh single transformants into 0.5 mL LB medium containing 100 mg L^{-1} ampicillin, followed by incubation at 28 °C with shaking at 400 rpm for 18 h. Main cultures were started by addition of 50 µL preculture into 1 mL TB medium supplemented with 100 mg L^{-1} ampicillin. The cultures were incubated at 28 °C, 400 rpm for 3.5 h, followed by induction with 1 mM IPTG and overnight incubation under the same conditions indicated above. Prior to cell lysis, the pellets were centrifuged, frozen on dry-ice for 30 min and thawed at room temperature for 30 min. Cells were disrupted by addition of 200 µL lysis buffer (0.1 M potassium phosphate buffer pH 7, 2 mM $MgCl_2$, 1 mg mL^{-1} lysozyme from chicken egg white, 0.75 mg mL^{-1} polymyxin B sulfate and 0.2 mg mL^{-1} DNase I). Cell suspensions were incubated at 30 °C with shaking at 300 rpm for 1 h, followed by centrifugation at 4 °C, 3,220 g for 30 min. Supernatants were immediately used for the UV-based activity assay.

Libraries L2 – L13. Precultures were started by inoculation of fresh single transformants or glycerol stocks into 500 µL LB medium containing 100 mg L^{-1} ampicillin, followed by incubation at 28 °C with shaking at 300 rpm for 18 h. Main cultures were started by inoculation of 25 µL preculture into 500 µL ZYM-5052 autoinduction medium without trace elements (10 g L^{-1} peptone, 5 g L^{-1} yeast extract, 5 g L^{-1} glycerol, 0.55 g L^{-1} glucose monohydrate, 2.1 g L^{-1} lactose monohydrate, 10.6 g L^{-1} sodium phosphate dibasic salt, 3.4 g L^{-1} potassium phosphate monobasic salt, 2.15 g L^{-1} ammonium chloride, 0.59 g L^{-1} sodium chloride, 0.663 g L^{-1} ammonium sulfate, 2 mM magnesium sulfate) supplemented with 100 mg L^{-1} ampicillin. The cultures were incubated at 20 °C, 300 rpm for 20 h in the same shaker. Optical cell densities at 600 nm were measured using a SpectraMAX Plus or Tecan Spark plate reader and corresponding software (SoftMax Pro 4.7.1 or SparkControl 2.1). Cell harvesting and lysis was performed as indicated above (protocol for L1). During the evolution campaign the cultivation protocol was optimized to yield more robust results. Changes included the use of fresh single transformants, a higher concentration of ampicillin as well as a lower inoculum volume to minimize beta-lactamase carry-over. Libraries L2 – L13, except L5, were measured at least once using the optimized protocol: Precultures were started by inoculation of fresh single transformants into 500 µL LB medium containing 200 mg L^{-1} ampicillin or, alternatively, two consecutive precultures using the same volume were prepared from glycerol stocks. Precultures were incubated at 28 °C with shaking at 300 rpm for 18 h. Main cultures were started by inoculation of 8 µL preculture into 500 µL ZYM-5052 autoinduction medium without trace elements and supplemented with 200 mg L^{-1} ampicillin. The cultures were incubated at 20 °C, 300 rpm for 20 h in the same shaker. After cell harvesting and lysis, freshly extracted supernatants were used for the UV-based activity assay.

1.5. KRED expression for biocatalytic reactions, lysate lyophilization or protein purification

Supplemented in-house KRED collection. Pre-cultures were started by inoculation of transformants into 1.2 mL LB medium containing 100 mg L^{-1} ampicillin, followed by incubation at 37 °C with shaking at 250 rpm for 18 h. Main cultures contained 1.2 mL LB with 100 mg L^{-1} ampicillin and were inoculated from the precultures using a microplate replicator. The cultures were incubated overnight at 37 °C and 250 rpm until an OD 600 nm of 0.5 - 0.6 was reached, followed by addition of 0.1 mM IPTG and incubation at 25 °C at 250 rpm for 24 h. Cells were harvested by centrifugation at 4 °C, 3,300 g for 20 min and the pellets were stored at - 20°C. Cells were lysed with 600 µL 30 mM MES buffer pH 6.6 containing 2 mM $MgCl_2$, 1 mg mL^{-1} lysozyme from chicken egg white, 0.75 mg mL^{-1} polymyxin B sulfate and 0.2 mg mL^{-1} DNase I, and shaking at 30 °C for 10 min. Fresh crude cell extracts (lysate and cell debris) were used for the initial screening (biocatalytic reaction).

0.2 mL- or ≥ 1 mL-scale biocatalytic reactions. For 0.2 mL-scale reactions, cells were grown using the optimized protocol for L2 – L13 and disrupted by enzymatic lysis or sonication. After the centrifugation step, cells were resuspended in 100 µL lysis buffer or in 100 µL of 0.1 M potassium phosphate buffer pH 7 containing 2 mM $MgCl_2$ for sonication. Resuspended pellets were pooled in a 50 mL Falcon tube and disrupted applying the following conditions: 50 % amplitude, 1 s on and 1 s off, 4 cycles with 1.5 min intervals on ice. Cell suspensions were centrifuged at 4° C, 20,000 g for 30 min. Protein concentration in clarified lysates was estimated using the BCA protein assay and bovine serum albumin as standard. For ≥ 1 mL-scale reactions, *Ssal*-KRED variants were grown in 20 mL LB with 100 mg L^{-1} ampicillin at 37 °C, 180 rpm overnight. Precultures (5 ml) were used to inoculate 2 L Erlenmeyer flasks containing 500 mL TB medium with 100 mg L^{-1} ampicillin, followed by incubation at 37 °C, 180 rpm until an OD 600 nm of 0.6 – 0.8 was reached. After addition of 1 mm IPTG, the cultures were incubated at 25 or 30 °C for 24 h. Cells were harvested by centrifugation at 4 °C, 9,300 g for 45 min. Pellets were weighed and resuspended in 2 mM potassium phosphate buffer pH 7 containing 0.04 mM $MgCl_2$ in a 1:2 biomass-buffer ratio. Cells were sonicated and centrifuged at 4 °C, 30,000 g for 20 min. Supernatants were lyophilized overnight using an Alpha 2-4 LDplus (Christ) set under – 85 °C and 0.14 mbar.

His-tagged *Ssal*-KRED wild-type and hit variants M1 – M6. Precultures were cultivated overnight in 5 mL LB with 50 mg L^{-1} kanamycin at 30 °C, 160 rpm and used to inoculate 500 mL autoinduction medium containing 50 mg L^{-1} kanamycin in a 2 L baffled Erlenmeyer flask. Main cultures were incubated at 20 °C, 110 rpm for 20 h. Cells were harvested by centrifugation at 4 °C, 3,300 g for 10 min. Cell pellets were resuspended in 30 mL Buffer A (50 mM potassium phosphate buffer pH 7.2 containing 2 mM $MgCl_2$) and disrupted by sonication using the same conditions indicated above. After centrifugation, fresh lysates were used for protein purification by FPLC on an ÄKTA pure system (GE Healthcare) equipped with a Ni-NTA column. Protein purification and desalting proceeded by using Buffer A (50 mM potassium phosphate buffer pH 7.2 containing 2 mM $MgCl_2$), Buffer B (Buffer A with 300 mM imidazole) and Desalting Buffer (100 mM potassium phosphate buffer pH 7.2 containing 2 mM $MgCl_2$). After desalting, proteins were concentrated in an Amicon centrifugal filter unit (10 kDa MWCO) at 4 °C and 3,300 g. Protein purity and concentration were respectively determined by SDS-PAGE analysis and absorbance at 280 nm using the molar extinction coefficient of each protein variant.

1.6. Lysate treatment prior to UV assay

Selected *Ssal*-KRED variants were subjected to heat or organic solvent treatment prior to the UV assay. Heat treatment of lysates was performed as reported elsewhere.[5] Lysates were either not heated and stored at 4 °C (non-treated control) or incubated at 50 °C for 30 min or 1 h, followed by immediate cooling down at 4 °C. To evaluate their solvent stability, lysates were either not exposed to iPrOH (non-treated control) or incubated together with 20 % (v/v) iPrOH at 10 °C with shaking at 1,000 rpm in a thermomixer for 1 or 2 h. Lysate volumes and concentrations of substrate stock solutions in iPrOH were adjusted to maintain the standard concentration of 8 % (v/v) iPrOH in the UV assay.

1.7. Sequencing and activity data analysis

Sequencing data was analyzed by aligning the Sanger FASTA sequencing files of multiple plates to the wild-type nucleotide sequence to identify on- and off-target mutations and output data were handled using Microsoft Excel (Version 2102, Build 13801.21004). The analysis of activity data was performed by determining the linear range of the curve by pairwise gradient estimation and *k*-means clustering.[6] Slopes (ΔA/min) were used to calculate the fold-improvement over the parent values (FIOP = slope of variant ÷ slope of parent). In addition, raw absorbance reads at 600 nm of main cultures were used as input to calculate the optical cell densities of the cultures the variant lysates derived from. In most cases they were comparable and only used to flag issues, thus FIOP values were not normalized with optical cell density values. Plate heatmaps of the cell density and FIOP values were generated for effective data visualization.

1.8. Small-scale biocatalytic reactions

Supplemented in-house KRED collection. Reactions were carried out in a 96-deep well plate format and using a final volume of 1.2 mL. Samples contained 30 mM MES buffer pH 6.6, 600 µL crude cell extract, 10 mM of **1a**, 1 mM NADH, 1 mM NADPH, 20 mM D-glucose monohydrate, 0.02 mg mL^{-1} glucose dehydrogenase (GDH-105, ~50 U mg^{-1}), and 2 mM $MgCl_2$. Plates were sealed and

incubated for 24 h at 30 °C with shaking at 250 rpm (Duetz system, Kuehner shaker). Next, 600 µL of the reaction mixture were transferred to a new 96-deep well plate and quenched with 600 µL HPLC-grade methanol, followed by centrifugation to separate the precipitated protein. Supernatants containing dissolved substrate and product were measured by HPLC-UV (260 nm) using one of the achiral methods described in **Supplementary Method 1.10**.

0.2 mL-scale reactions at 100 g L^{-1} substrate with fresh lysates. Reactions were carried out in 2 mL Eppendorf tubes. Samples contained 20 mg of **1a**, 0.1 M potassium phosphate buffer pH 7.2, 2 mM $MgCl_2$, 0.1 % (w/v) $NADP^+$ disodium salt (s/c = 100), clarified lysate equivalent to 3.5 mg mL^{-1} total protein (determined by the BCA assay) and 8 % (v/v) iPrOH in 2 mL Eppendorf tubes. Reactions were run in a thermomixer at 25 °C and with shaking at 1,000 rpm. After a given time, reactions were quenched with HPLC-grade methanol to achieve a final dilution factor of 100. Samples were centrifuged to separate the precipitated protein. Supernatants containing dissolved substrate and product were measured by HPLC-UV (260 nm) using one of the achiral methods described in **Supplementary Method 1.10**.

1 mL-scale reactions at 100 g L^{-1} substrate with lyophilized lysates. Samples contained 0.1 g of **1a**, 0.1 M potassium phosphate buffer pH 7.2, 2 mM $MgCl_2$, 0.1 % (w/v) $NADP^+$ disodium salt (s/c = 100), 8 % (v/v) iPrOH and lyophilized lysate derived from selected variants and controls in different substrate-to-enzyme (s/e) ratios. Reactions were run at 23 - 30 °C and with shaking at 1,500 rpm. After a given time, reactions were quenched with HPLC-grade methanol and analyzed by HPLC-UV (260 nm) using one of the achiral methods described in described in **Supplementary Method 1.10**.

1.9. Preparative-scale biocatalytic reactions

100 mL-scale reactions using iPrOH as final reductant. The reaction was performed in a 200 mL 3-necked sulfonation flask with a KPG-stirrer system and N_2 flow. The flask contained **1a** (10 g, 0.03 mol, 1 eq), water (39 mL), 1 M potassium phosphate buffer pH 7.2 (10 mL), 0.1 M $MgCl_2.6H_2O$ (2 mL), iPrOH (8 mL) and $NADP^+$ (100 mg, 0.004 eq, s/c = 100, previously dissolved in 1 mL). After stirring for 5 min, the reaction was started by addition of M6 lyophilized lysate (2 g, s/e = 5, previously dissolved in 30 mL water), followed by incubation at 23 °C for 30 h. Afterwards, 0.1 mL reaction mixture was quenched with 0.9 mL HPLC-grade methanol, vortexed, centrifuged (3,220 g, 23 °C, 5 min) and filtered for achiral HPLC-UV analysis. The reaction mixture was transferred to a 500 mL pear flask. The reaction vessel was rinsed twice with 10 mL water each time and collected in the new flask. Using a rotary evaporator, the suspension was subjected to 40 °C, 200 – 60 mbar for iPrOH/water evaporation. The suspension was cooled down to room temperature and then, using a Büchner funnel, it was filtered off on a P3 suction filter (7 cm), washed twice with 25 mL water (total = 50 mL) and twice with 25 mL heptane (total = 50 mL). The funnel was left standing upside down on weighing paper in the chemical hood. The crude product was dried at 40 °C/< 10 mbar to constant weight. 9.5 g (94.2 %) light beige powder as crude product was isolated. For chiral HPLC-UV analysis, 10 mg product was mixed with 1 mL HPLC-grade methanol, vortexed and centrifuged. Then, 100 µL of the previous solution was mixed with 900 µL methanol and filtered. The product was obtained with a HPLC purity of 99.7 area % **2a** (0.0 area % (*S*,*S*)-*trans*-product, 0.1 area % *cis*-products, 0.2 area % substrate) (**Supplementary Fig. 14**). **2a** ESI-MS (positive): 335.21 ($M+H^+$; $C_{17}H_{26}N_4O_3 + H^+$) (**Supplementary Fig. 15**). **2a** ^1H NMR (600 MHz, $CDCl_3$) δ ppm 8.54 (s, 1 H), 5.11 (t, *J*=7.2 Hz, 1 H), 3.78 (ddd, *J*=13.1, 7.3, 3.3 Hz, 2 H), 3.64 (ddd, *J*=13.0, 6.9, 3.3 Hz, 2 H), 3.46 – 3.60 (m, 5 H), 3.24 – 3.46 (m, 1 H), 2.12 – 2.23 (m, 2 H), 1.49 (s, 9 H), 1.20 (d, *J*=7.1 Hz, 3 H). (**Supplementary Data 1**).

100 mL-scale reactions using glucose as final reductant. To evaluate the performance of M6 at a lower biocatalyst concentration, the reaction was conducted in the presence of iPrOH and glucose in a pH-STAT 902 Titrando equipped with a 175 mL glass vessel, pH probe, NaOH-inlet and 900 Touch control. The reaction vessel contained **1a** (10 g, 0.03 mol, 1 eq), water (34 mL), 1 M potassium phosphate buffer pH 7.2 (10 mL), 1 M D(+)-glucose monohydrate (7.13 g, 1.2 eq, 36 mL), 0.1 M $MgCl_2.6H_2O$ (2 mL), iPrOH (8 mL) and $NADP^+$ (100 mg, 0.004 eq, s/c = 100) and GDH-105 (100 mg, s/e = 100). After stirring for 5 min, the reaction was started by addition of M6 lyophilized lysate (0.2 g, s/e = 50). The pH was maintained constant by addition of 1 M NaOH (Titrisol) (30.08 mL, 0.03 mol, 1 eq). The reaction was incubated at 23 °C for 27 h. Sample analysis and product isolation were performed as described in the previous paragraph. 9.5 g (94.0%) light beige powder as crude product was isolated with a HPLC purity of 99.5 area % **2a** (0.0 area % (*S*,*S*)-*trans*-product, < 0.05 area % *cis*-product, 0.4 area % substrate) (**Supplementary Fig. 17**).

1.10. HPLC-UV analysis of substrate and products

Achiral method. Reaction mixtures were quenched with HPLC-grade methanol in a convenient ratio according to the concentration of substrate. Samples were centrifuged at 4 °C, 3,300 g for 10 min after protein precipitation. Supernatants were analyzed by LC-UV at 260 nm on an Agilent 1290 HPLC system using one of the following methods: a) Kinetex XB-C18 column (50 mm x 4.6 mm, 2.6 µm), using water and methanol as solvents A and B, respectively. The column was heated at 50 °C, the flow rate was 0.8 mL min^{-1}, and the injection volume was 2 µL. The following gradient was used: 0 – 0.1 min, B = 50 %; 0.1 – 4 min, B = 50 – 58 %; 4 – 4.1 min, B = 58 – 95 %; 4.1 – 4.6 min, B = 95 %; 4.6 – 4.7 min, B = 95 – 50 %; 4.7 – 5 min, B = 50 %, 5 – 5.1 min, B = 50 – 95 %; 5.1 – 5.4 min, B = 95 %; 5.4 – 5.5 min, B = 95 – 50 %; and 5.5 – 5.9 min, B = 50 %. b) Agilent InfinityLab Poroshell 120 Eclipse EC-C18 column (50 mm x 3.0 mm x 2.7 µm), using the same mobile phases, column temperature, flow rate and injection volume. The following gradient was used: 0 – 0.1 min, B = 40 %; 0.1 – 6 min, B = 40 – 52.7 %; 6 – 6.2 min, B = 52.7 – 95 %; 6.2 – 7.2 min, B = 95 %; 7.2 – 8 min, B = 95 – 40 %; 8 – 9 min, B = 40 %. c) Kinetex EVO-C18 column (50 mm x 4.6 mm, 5 µm), using the same mobile phases. The

column was heated at 40 °C, the flow rate was 2 mL min^{-1}, and the injection volume was 1 µL. The following isocratic method was applied: 0 – 7.5 min, B = 40 %. The method a or b was used after UV assay screening or 0.2 mL-scale biocatalytic reactions. Standards **1a**, **2a** and **2b** were treated as the samples prior to the HPLC-UV analysis.

Agilent OpenLAB CDS (2.4, Build 2.204.0661) was utilized for the acquisition of LC-UV/MS data. For quick verification of selectivity from UV assay samples, diastereomeric excess values were estimated from the relative peak areas of the alcohol products. In case of 0.2 mL-scale biocatalytic reactions, conversions were calculated using a calibration curve of **2a**. Diastereomeric excess values were estimated using the relative peak areas corresponding to the retention times of **2a** and **2b** (e.g., **Supplementary Fig. 4**). Method c was applied on a second site for ≥ 1 mL-scale reactions. Conversion levels and selectivity were determined from the relative peak areas.

Chiral method. This method was used to estimate HPLC product purity (as area %) from 100 mL-scale reactions. LC-UV analysis was carried out at 254 nm using an Agilent 1290 HPLC system equipped with a Chiralpak IC-3 column (150 mm x 4.6 mm, 3 µm) heated at 30°C, using heptane and ethanol + 0.1 % diethanolamine as solvents A and B, respectively. The flow rate was 0.8 mL min^{-1} and the injection volume was 5 µL. The following gradient was used: 0 – 5 min, B = 40 %; 5 – 15 min, B = 100 %; 15 – 17 min, B = 100 %, 17 – 17.1 min, B: 40 %. The retention times of each substrate and product stereoisomer are indicated in **Supplementary Fig. 14** and **Supplementary Fig. 17**.

1.11. Determination of kinetic constants

Reactions were measured in 96-well Greiner microtiter plates in a final volume of 200 µL containing: (1) 178 µL of 0.1 M potassium phosphate buffer pH 7 with 2 mM $MgCl_2$ and 0.01 mg mL^{-1} $NADP^+$, (2) 6 µL diluted purified protein in a final concentration of 0.1 - 1 µM (adjusted according to each variant); and (3) 16 µL of a stock solution of **1a** in iPrOH for a final concentration of 1 - 800 µM. The substrate consumption rate was measured at 340 nm, 25 °C on a Tecan Infinite M Nano+ spectrophotometer. A calibration curve of **1a** enabled the calculation of amount of substrate consumed per time unit. Kinetic constants were calculated by non-linear regression using Prism 9.2.0 (GraphPad Software, San Diego, CA, USA). Michaelis-Menten plots were generated using the same software.

1.12. Illustrations

Microsoft Excel, BioRender, Adobe Illustrator 24.1.1, ChemDraw 21.0, PyMOL 2.5.x, Plotly (5.x) Python (3.8.x) were employed for illustration purposes.

2. Supplementary Figures

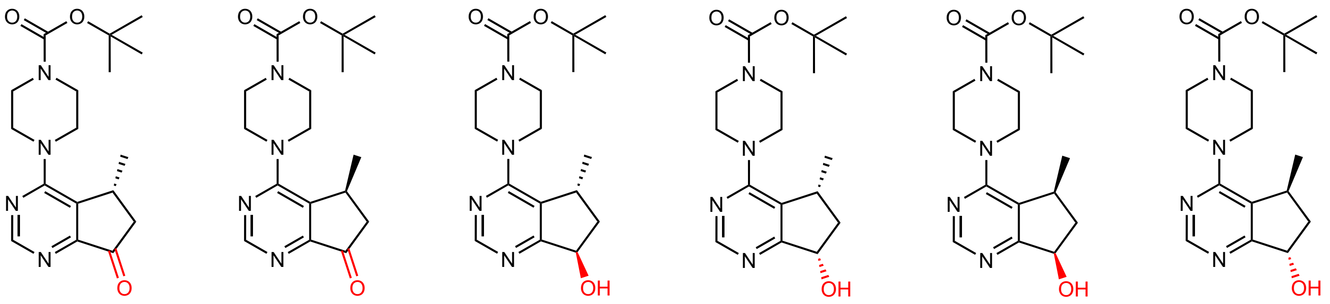

1a	1b	2a	2b	2c	2d
(*R*)	(*S*)	(*R*,*R*)-*trans*	(*R*,*S*)-*cis*	(*S*,*R*)-*cis*	(*S*,*S*)-*trans*

Supplementary Figure 1. Starting material and products. Structures of ketone enantiomers **1a** and **1b** and all four possible diastereomeric alcohol products (**2a** – **2d**) derived thereof. Compounds **1a** and **2a** are the desired substrate and product, respectively.

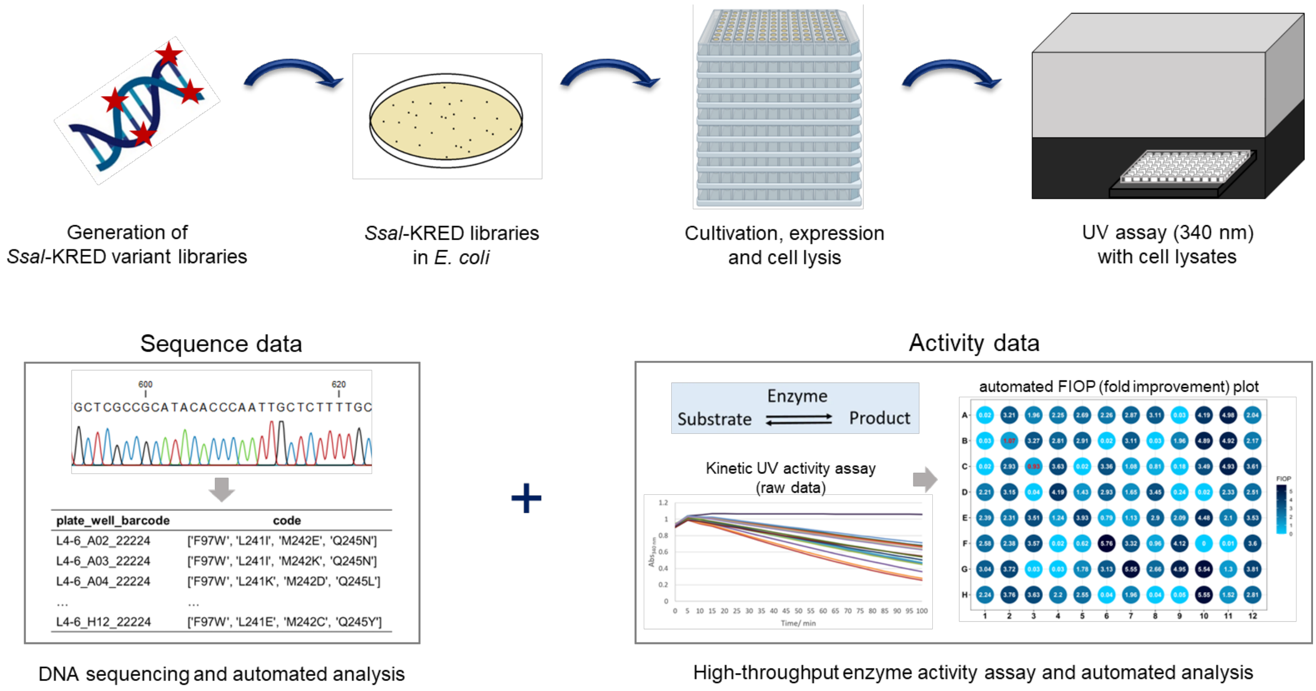

Supplementary Figure 2. *Ssal*-KRED engineering workflow and sequence-activity data collection. Fold-improvement over the parent (FIOP) was calculated as follows: FIOP = initial reaction rate of variant ÷ initial reaction rate of parent. Images created with BioRender.

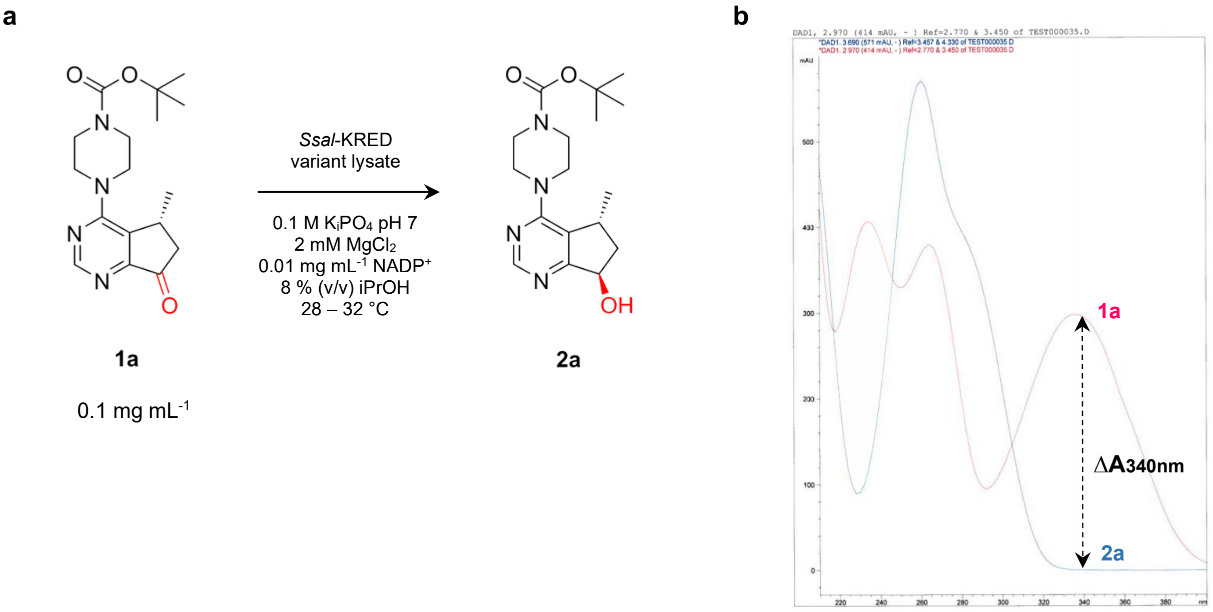

Supplementary Figure 3. UV kinetic assay conditions and principle. a Assay conditions. **b** The consumption rate of **1a** is followed at 340 nm.

a

Q245T, 7.5 mM **1a** (zoomed HPLC-UV chromatogram)

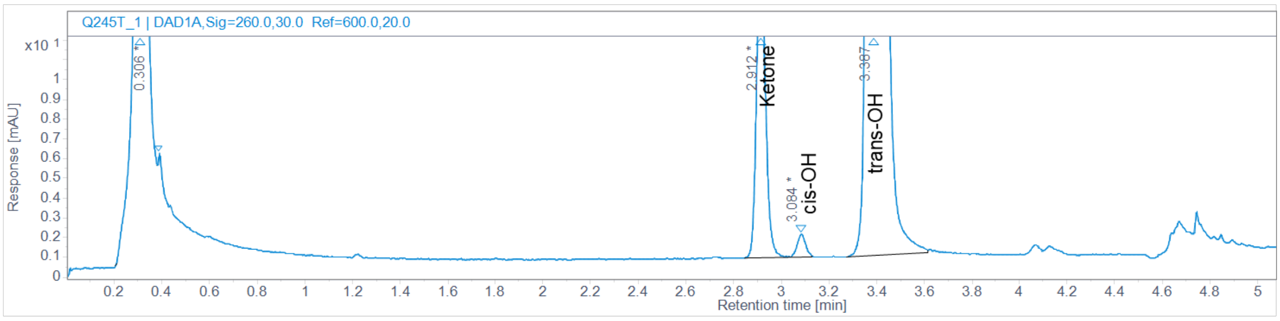

b

M1, 7.5 mM **1a** (zoomed HPLC-UV chromatogram)

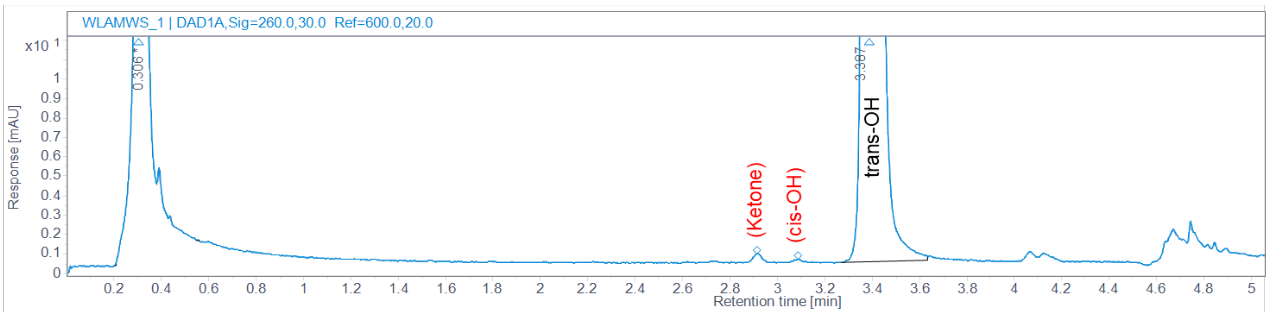

Supplementary Figure 4. Achiral HPLC-UV chromatograms of variants Q245T and M1. a Q245T exhibited a diastereomeric excess of 99.7 % (*trans*). **b** M1 exhibited a diastereomeric excess > 99.9 % (*trans*). The 0.2 mL-scale reactions contained 7.5 mM **1a** and were incubated at 28 °C for 1 h.

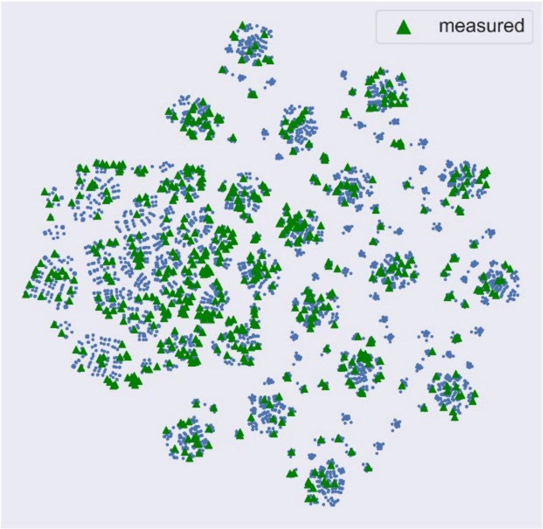

Supplementary Figure 5. t-distributed Stochastic Neighbor Embedding (t-SNE)[7] representation of the encoded enzyme variants of L4. Blue dots represent the sequence space (all possible combinations, 8’000 in total), while the measured variants are highlighted in green.

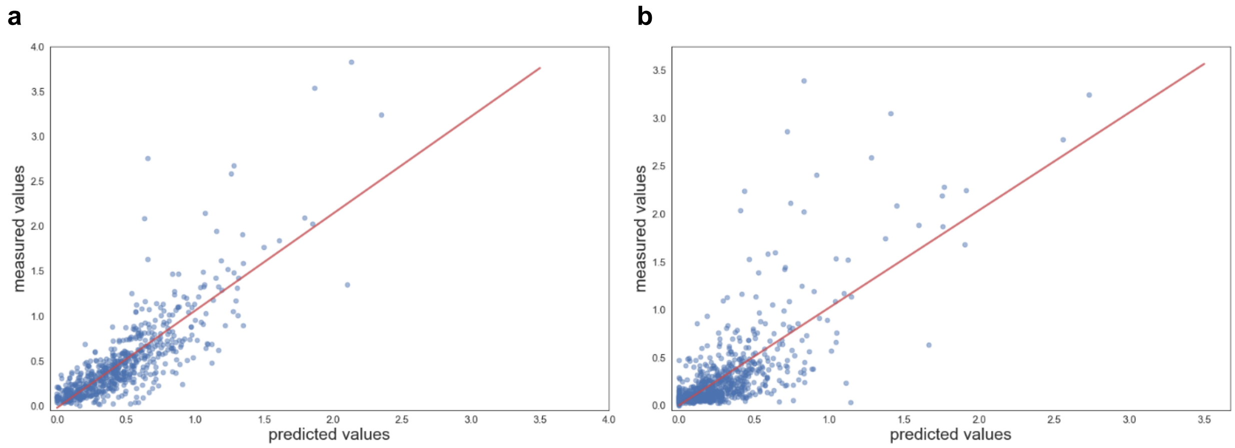

Supplementary Figure 6. Out-of-fold predicted vs. measured values. a L9 results: the activity values were predicted (*x*-axis) using a Gaussian process, trained on the measured data (*y*-axis). An R^2 score of 0.66 was noted. **b** L10 results: The training data included 3-site CSM libraries L3, L4, L6 and 5-site CSM library L7. The out-of-fold predictions on L7 vs. the actual measured values are shown. An R^2 score of 0.77 was noted. Measured and predicted values are provided in https://github.com/ccbiozhaw/Ssal-KRED_evolution.

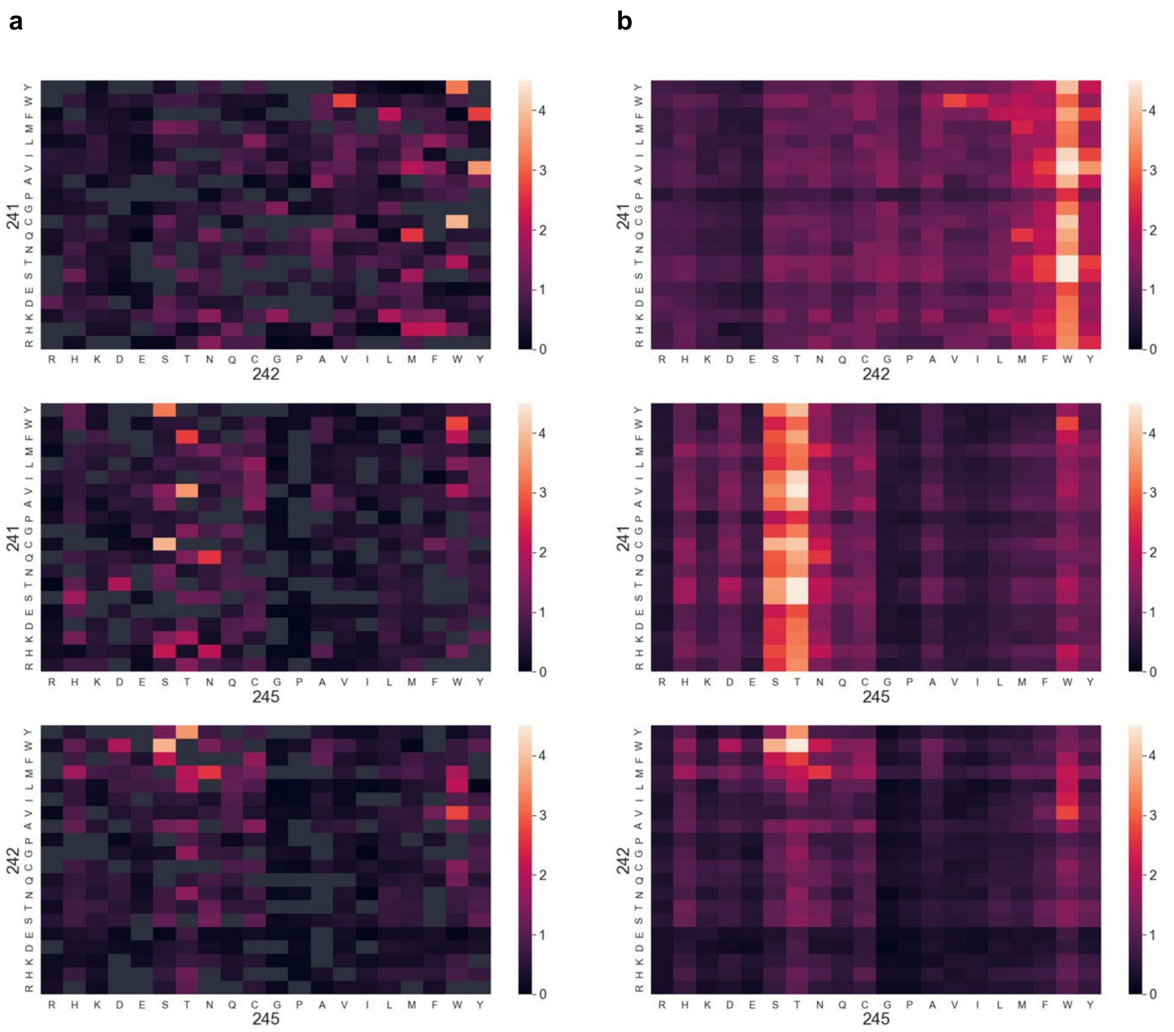

Supplementary Figure 7. Overview of measured and predicted improvements for key positions 241, 242 and 245 (Library L4). a Heatmaps of the measured activity of a residue pair irrespective of the mutation at the third position. Gray boxes denote amino acid combinations which were not measured in the screening of L4. **b** Measured data is supplemented with predicted data for all remaining amino acid combinations. Distinct hotspots appear, especially for positions 242 and 245. This analysis enabled us to select influential residues for recombination. Measured and predicted values are provided in https://github.com/ccbiozhaw/Ssal-KRED_evolution.

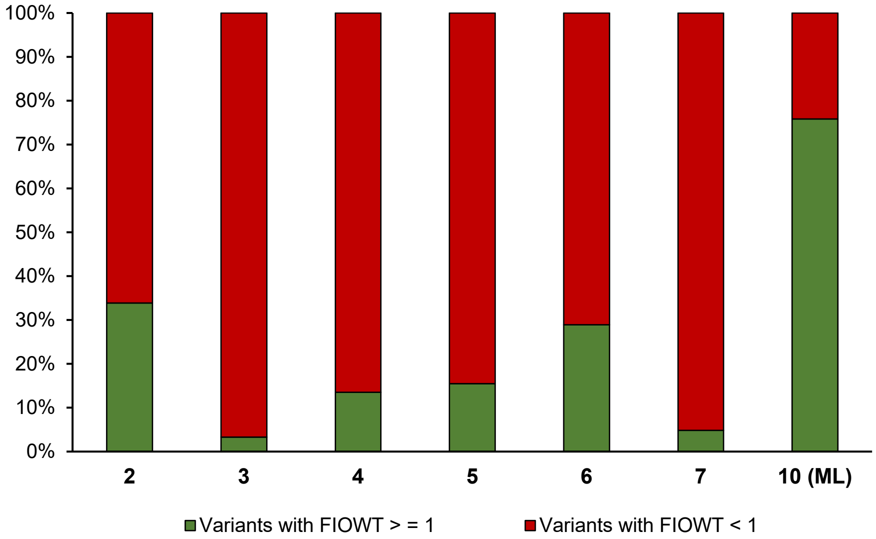

Library	sites targeted	# positive variants (FIOWT ≥ 1)	# negative variants (FIOWT < 1)	# total variants	% positive variants (FIOWT ≥ 1)	% negative variants (FIOWT < 1)
**2**	97, 174, 238, 241, 242, 245	39	76	115	33.9	66.1
**3**	174-242-245	15	435	450	3.3	96.7
**4**	241-242-245	92	588	680	13.5	86.5
**5**	238-242-245	68	372	440	15.5	84.5
**6**	174-238-241	163	400	563	29	71
**7**	174-238-241-242-245	37	725	762	4.9	95.1
**10 (ML)**	(174-238-241-242-245)	261	83	344	75.9	24.1

Supplementary Figure 8. Distribution of “positive” (FIOWT ≥ 1) and “negative” (FIOWT < 1) variants screened in libraries L2 – L7 as well as in the machine learning (ML)-filtered library L10. Unique on-target variants are considered for libraries L2 – L7 as sequence-function results were available for these datapoints. In case of L10, the total number of screened transformants is considered as only the hits were sequenced. For L10, predicted variants with mutations on the 5 sites were obtained, followed by a filtering process according to criteria specified in **Supplementary Table 13**.

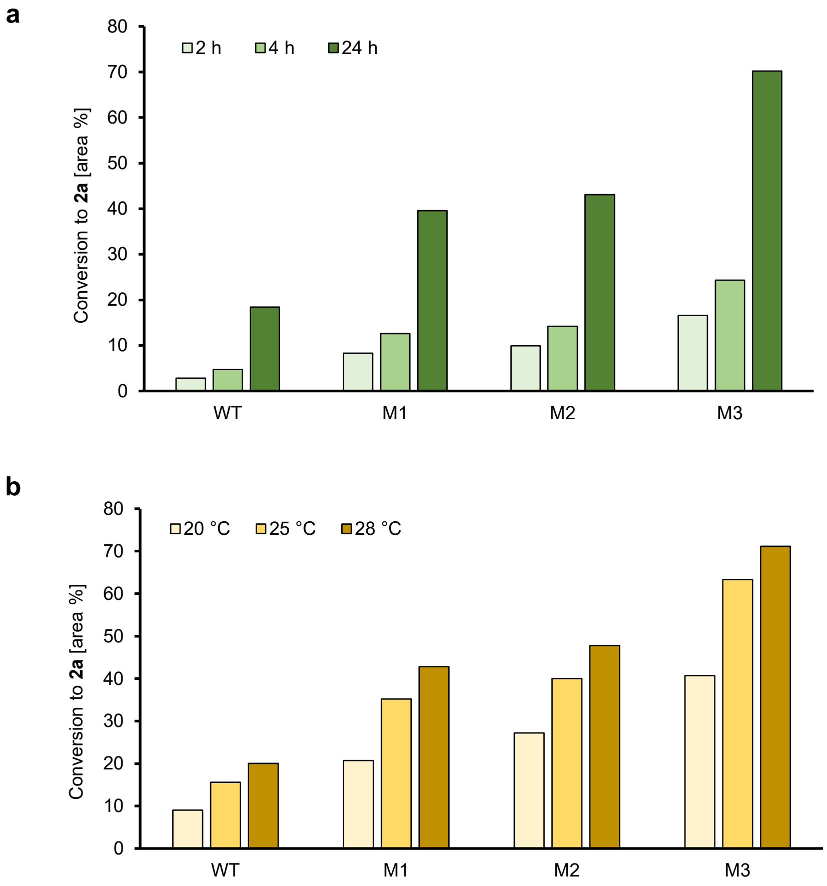

Supplementary Figure 9. 0.2 mL-scale reactions at 100 g L^{-1} substrate catalyzed by the wild type (WT) and hit variants M1, M2 and M3. **a** Reactions after 2, 4 and 24 h at 28 °C. **b** Reactions conducted at 20, 25 and 28 °C for 24 h. Reactions (0.2 mL) contained 0.1 M potassium phosphate buffer pH 7.2, 2 mM $MgCl_2$, 20 mg of **1a**, 3.5 mg mL^{-1} total protein (fresh lysate), 0.1 % (w/v) $NADP^+$ and 8 % (v/v) iPrOH. These experiments were conducted once. Source data for this figure is available (**Supplementary Data 3**).

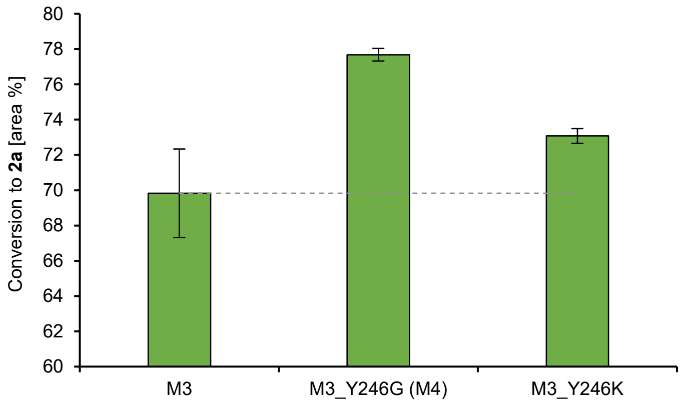

Supplementary Figure 10. 0.2 mL-scale reactions with 100 g L^{-1} substrate using the L11 hits and parent M3. Reactions were performed in duplicates in a volume of 0.2 mL containing 0.1 M potassium phosphate buffer pH 7.2 with 2 mM $MgCl_2$, 20 mg of **1a**, 3.5 mg mL^{-1} total protein (fresh lysate), 8 % (v/v) iPrOH and 0.1 % (w/v) $NADP^+$. Reactions were incubated at 25 °C with shaking at 1,000 rpm for 24 h. This experiment was conducted in duplicates. Source data for this figure is available (**Supplementary Data 4**).

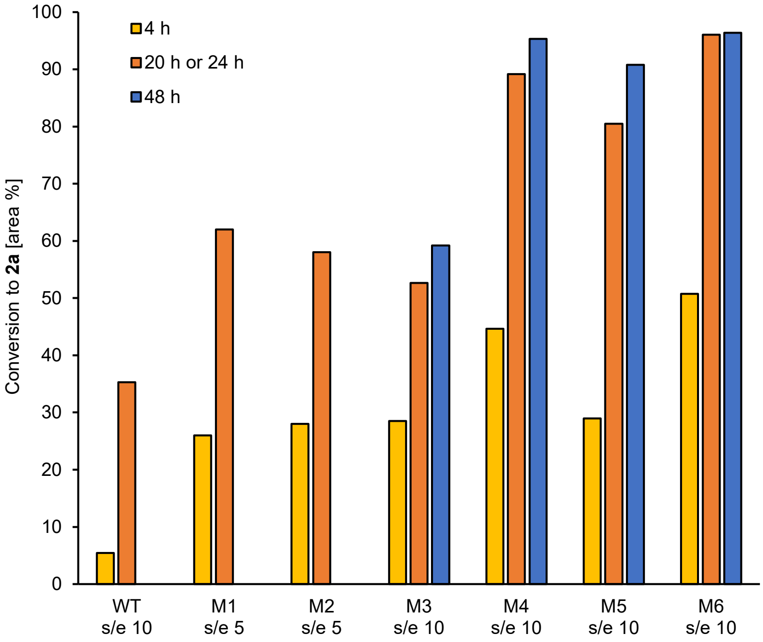

Supplementary Figure 11. Comparison of performance of the wild-type enzyme and hit variants in 1 mL-scale reactions at 100 g L^{-1} substrate. Reactions were conducted at 30 °C and in the presence of iPrOH as the cofactor recycling system. All *Ssal*-KRED variants were tested as lyophilized enzyme lysates in a substrate-to-enzyme (s/e) ratio of 10 and sampled at least after 4 h and 24 h, except for M1 and M2, which were tested in a s/e of 5 and sampled after 4 h and 20 h. In all cases, the diastereomeric excess (*de*) was > 99.5 %. This experiment was conducted once. Source data for this figure is available (**Supplementary Data 5**).

a b

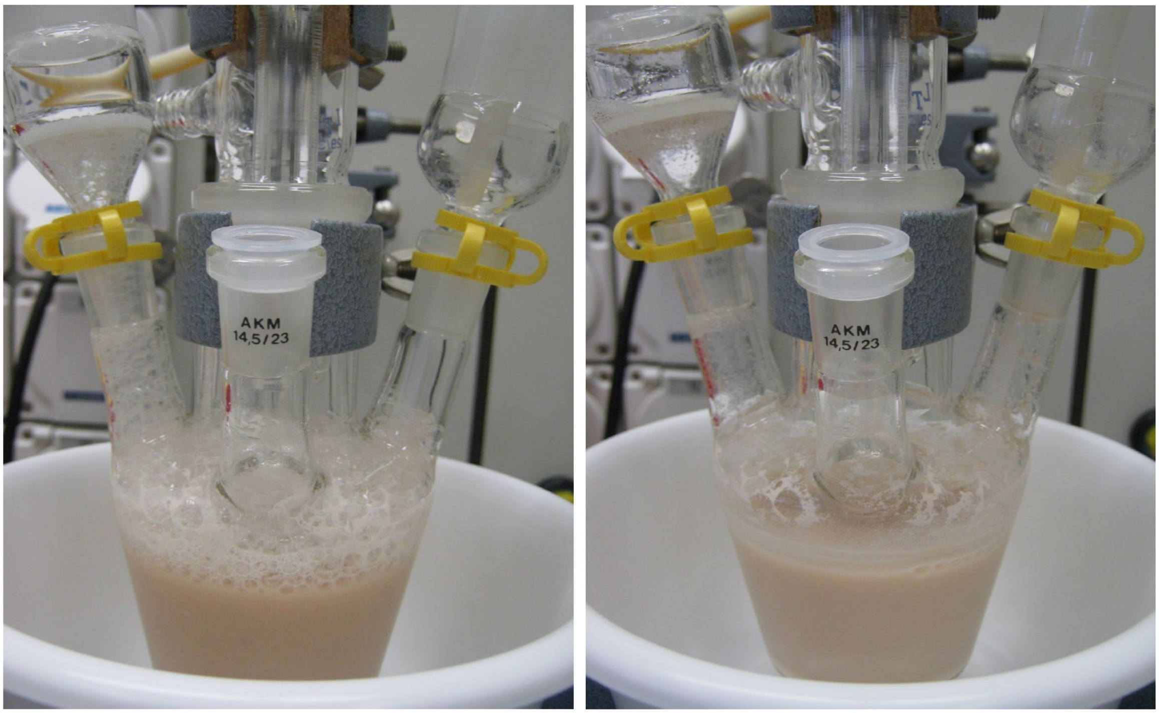

Supplementary Figure 12. 100 mL-scale reaction set up for reduction of 1a by variant M6 in the iPrOH-based recycling system. a Start time. **b** After 4 hours. The reaction vessel contained **1a** (10 g, 0.03 mol, 1 eq), water (39 mL), 1 M potassium phosphate buffer pH 7.2 (10 mL), 0.1 M $MgCl_2.6H_2O$ (2 mL), iPrOH (8 mL), $NADP^+$ (100 mg, 0.004 eq, s/c = 100, previously dissolved in 1 mL), and M6 lyophilized lysate (2 g, s/e = 5, previously dissolved in 30 mL water). The reaction was run at 23 °C for 30 h.

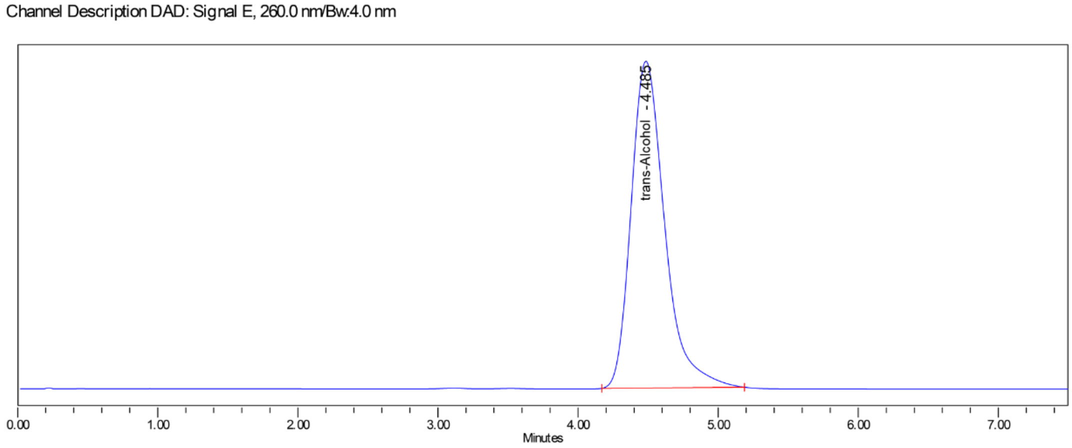

	RT (min)	Type	Width (sec)	Height (µV)	Area (µV*s)	% Area	Name	Compound Number
1	3.270	Missing					Ketone	**1a + 1b**
2	3.740	Missing					cis-Alcohol	**2b + 2c**
3	4.485	BB	61.2	315285.0	5250613.5	100.0	trans-Alcohol	**2a + 2d**
None								
Sum					5250613.5	100.0		
Max			61.2	315285.0				

Supplementary Figure 13. Achiral HPLC-UV chromatogram of 100 mL-scale reaction catalyzed by M6 using iPrOH as reductant.

Channel Description DAD: Signal A, 254.0 nm/Bw:4.0 nm

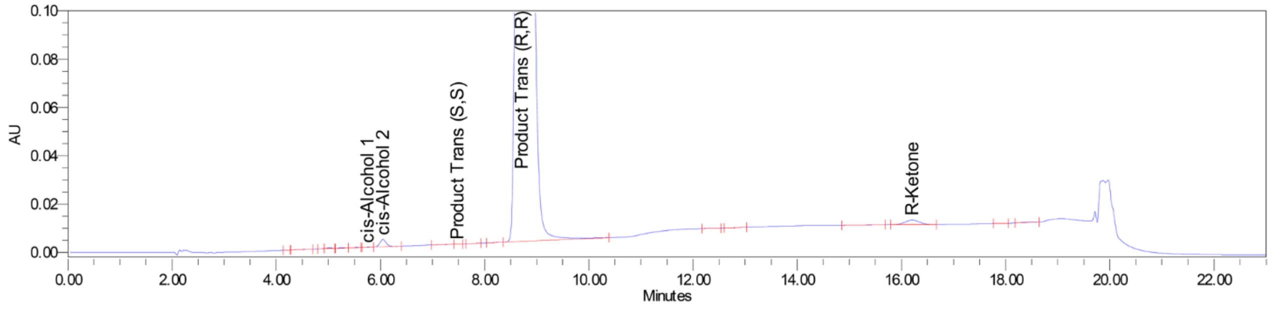

	RT (min)	Height (µV)	Area (µV*s)	% Area (adj.)	Content	Name	Compound Number
1	4.229	7.288	30.9438	0.0002	0.0000		
2	4.590	110.472	814.2829	0.0042	0.0000		
3	4.873	35.941	138.8906	0.0007	0.0000		
4	5.019	280.324	1558.0297	0.0081	0.0000		
5	5.249	189.071	1255.8547	0.0065	0.0000		
6	5.490	35.347	299.9698	0.0016	0.0000		
7	5.752	27.348	213.2892	0.0011	0.0011	cis-Alcohol 1	**2c**
8	6.046	2972.987	24753.3147	0.1290	0.1291	cis-Alcohol 2	**2b**
9	7.205	32.884	390.1904	0.0020	0.0000		
10	7.477	13.078	70.1680	0.0004	0.0004	Product Trans (S,S)	**2d**
11	7.864	8.354	74.5982	0.0004	0.0000		
12	8.200	24.567	233.6006	0.0012	0.0000		
13	8.715	1472692.040	19115019.9876	99.6358	99.6825	Product Trans (R,R)	**2a**
14	12.270	18.290	211.8170	0.0011	0.0000		
15	12.678	24.044	271.8387	0.0014	0.0000		
16	13.800				0.0000	S-Ketone	**1b**
17	15.334	33.235	733.3594	0.0038	0.0000		
18	16.207	1855.025	35843.8129	0.1868	0.1869	R-Ketone	**1a**
19	17.918	23.672	252.3904	0.0013	0.0000		
20	18.316	192.673	2734.5718	0.0143	0.0000		
Sum			19184900.9103	100.0000	100.0000		
Max		1472692.040					

Supplementary Figure 14. Chiral HPLC-UV chromatogram of 100 mL-scale reaction catalyzed by M6 using iPrOH as reductant.

Compound Label	m/z	RT	Algorithm	Mass
Cpd 1: C17 H26 N4 O3	335.2083	1.67	Find By Formula	334.2011

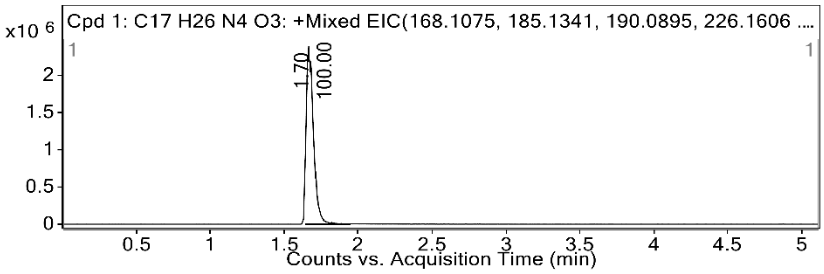

MS Spectrum

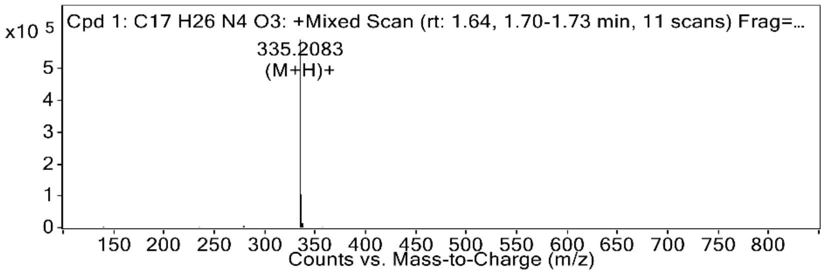

MS Zoomed Spectrum

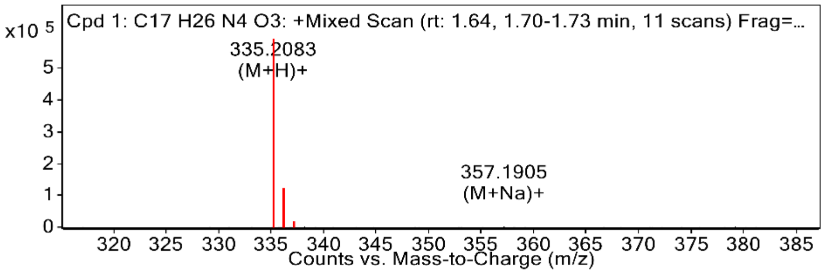

Supplementary Figure 15. Mass spectra of 2a from 100 mL-scale reaction catalyzed by M6 using iPrOH as reductant.

Channel Description DAD: Signal E, 260.0 nm/Bw:4.0 nm

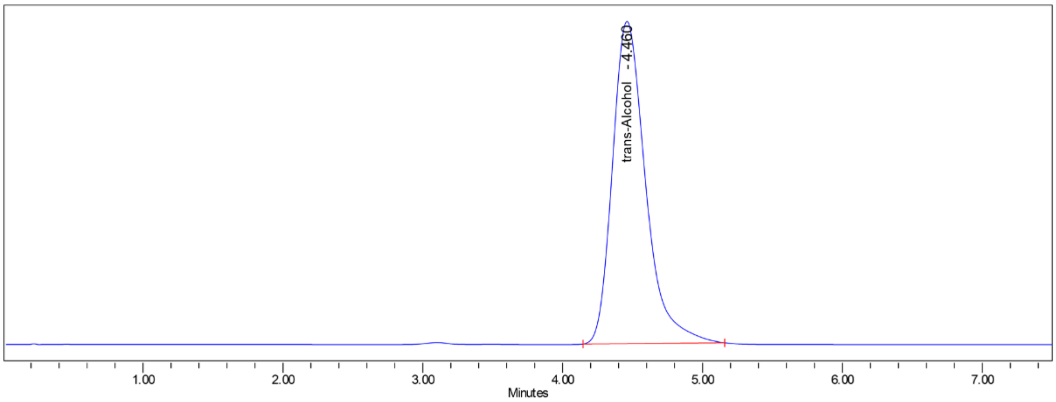

	RT (min)	Type	Width (sec)	Height (µV)	Area (µV*s)	% Area	Name	Compound Number
1	3.270	Missing					Ketone	**1a + 1b**
2	3.740	Missing					cis-Alcohol	**2b + 2c**
3	4.460	BB	60.8	332249.5	5469163.0	100.0	trans-Alcohol	**2a + 2d**
None								
Sum					5469163.0	100.0		
Max			60.8	332249.5				

Supplementary Figure 16. Achiral PLC-UV chromatogram of 100 mL-scale reaction catalyzed by M6 using glucose as reductant.

Channel Description DAD: Signal A, 254.0 nm/Bw:4.0 nm

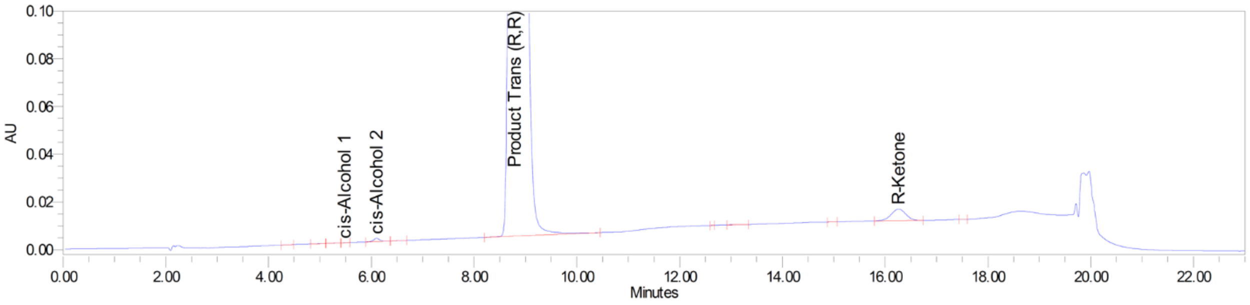

	RT (min)	Height (µV)	Area (µV*s)	% Area (adj.)	Content	Name	Compound Number
1	4.349	28.553	191.5239	0.0009	0.0000		
2	5.014	50.134	482.6024	0.0022	0.0000		
3	5.179	24.051	244.1911	0.0011	0.0000		
4	5.476	17.078	74.5061	0.0003	0.0003	cis-Alcohol 1	**2c**
5	6.100	1297.082	10886.6093	0.0491	0.0491	cis-Alcohol 2	**2b**
6	6.572	25.997	218.4781	0.0010	0.0000		
7	7.643				0.0000	Product Trans (S,S)	**2d**
8	8.792	1670711.151	22059307.3303	99.5098	99.5202	Product Trans (R,R)	**2a**
9	12.634	16.594	40.0930	0.0002	0.0000		
10	13.096	89.318	887.9060	0.0040	0.0000		
11	13.800				0.0000	S-Ketone	**1b**
12	14.948	35.617	173.9426	0.0008	0.0000		
13	16.261	4866.088	95398.0111	0.4303	0.4304	R-Ketone	**1a**
14	17.477	20.199	78.5679	0.0004	0.0000		
Sum			22167983.7617	100.0000	100.0000		
Max		1670711.151					

Supplementary Figure 17. Chiral HPLC-UV chromatogram of 100 mL-scale reaction catalyzed by M6 using glucose as reductant.

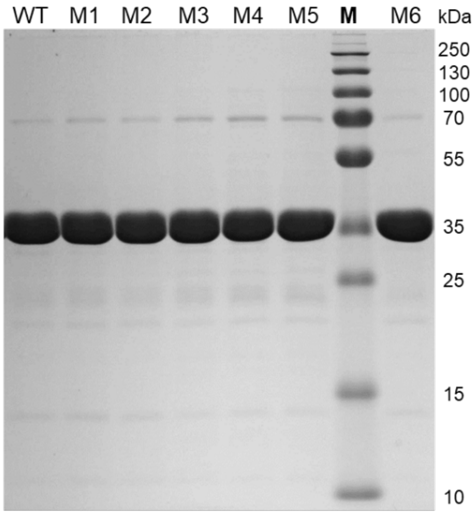

Supplementary Figure 18. SDS-PAGE analysis of purified N-terminally 6xHis-tagged wild-type *SsaI*-KRED (WT) and selected variants. Samples (10 µg protein measured using absorbance values at 280 nm and the molar extinction coefficient of each variant) were loaded on a 12.5 % acrylamide gel. The theoretical molecular weight of the His-tagged WT is 39.5 kDa.

a

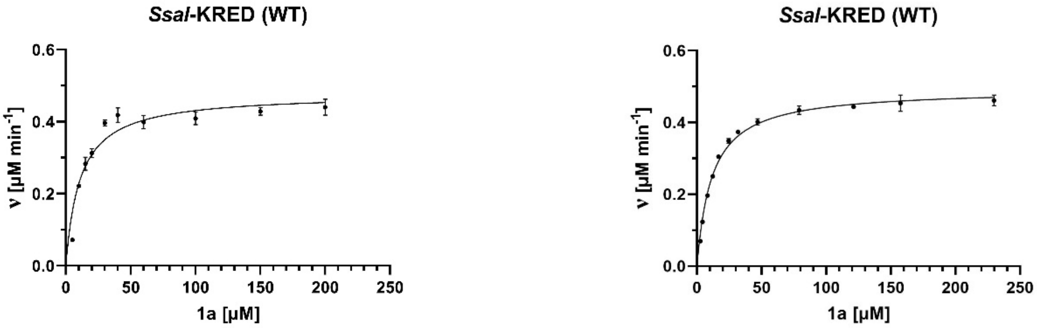

UV kinetic assay

Parameter	Values
app. V_{max} [µM min^{-1}]	0.487 ± 0.018
app. K_M [mM]	0.0119 ± 0.0011
E_0 [µM]	1.0
app. k_{cat} [min^{-1}]	0.487 ± 0.018
app. k_{cat}/K_M [min^{-1} mM^{-1}]	41 ± 2.2

HPLC-UV

Parameter	Values
app. V_{max} [µM min^{-1}]	0.493 ± 0.0152
app. K_M [mM]	0.0115 ± 0.0005
E_0 [µM]	1.0
app. k_{cat} [min^{-1}]	0.493 ± 0.015
app. k_{cat}/K_M [min^{-1} mM^{-1}]	43.1 ± 0.6

b

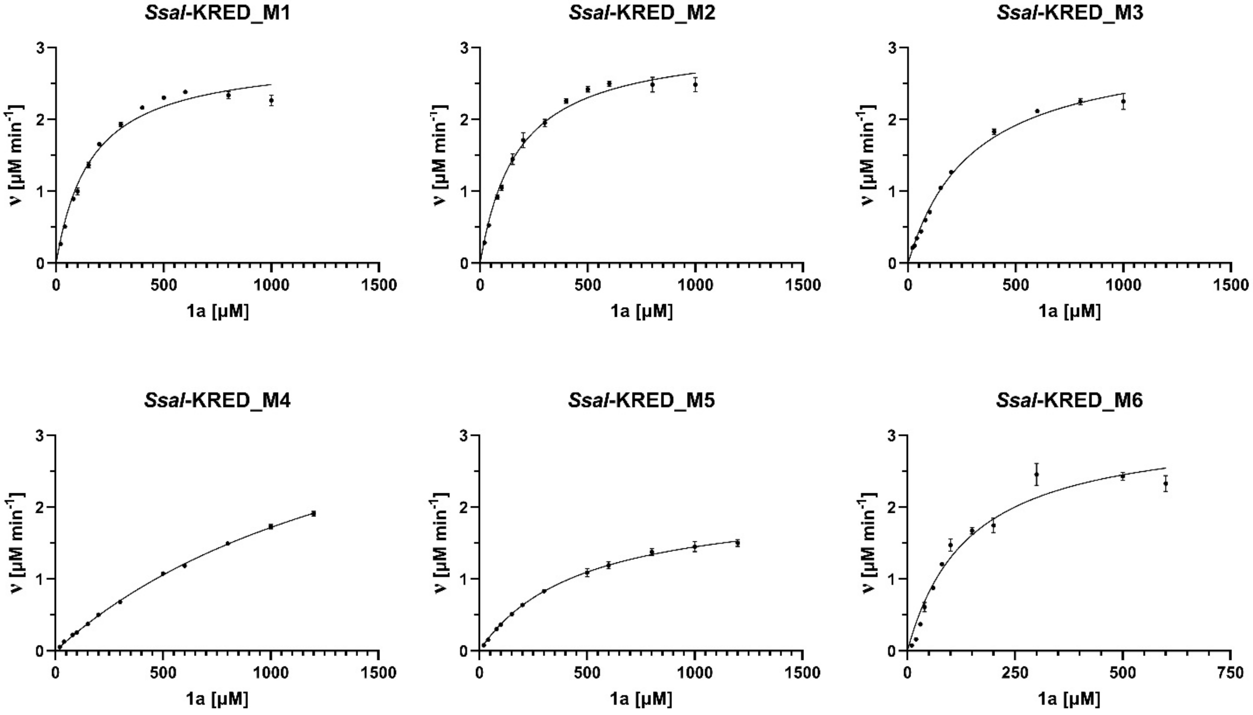

Parameter	WT	M1	M2	M3	M4[a]	M5	M6
**app. V_{max} [µM min^{-1}]**	0.487 ± 0.018	2.89 ± 0.055	3.109 ± 0.095	3.068 ± 0.072	4.582 ± 0.235	2.130 ± 0.098	3.153 ± 0.081
**E_0 [µM]**	1	0.5	0.5	0.2	0.1	0.1	0.1

[a] saturation was not reached.

Supplementary Figure 19. Determination of apparent kinetic constants towards 1a. **a** Michaelis-Menten plots of wild-type *Ssal*-KRED (WT) measured in triplicate via the UV assay (left) and HPLC-UV (right). **b** Michaelis-Menten plots of hit variants from measurements in triplicate using the UV assay. Apparent V_{max} and enzyme concentrations are indicated. Source data for this figure is available (**Supplementary Data 6**).

3. Supplementary Tables

Supplementary Table 1. Activity and selectivity of the in-house KRED collection[a] towards **1a**.

KRED	Source organism	Protein ID	retrieved from Ref.	Conversion [%]	selectivity	*de* [%]
1	*Lactobacillus brevis*	1ZJY_A	8	9.0	*cis*	35
2	*Lactobacillus brevis*	Q84EX5	9	22.0	*cis*	42
**3**	***Lactobacillus hokkaidonensis***	**A0A0A1GQU9**	**-**	**12.8**	***trans***	**27**
4	*Lactobacillus kefiri*	4RF5_A	10	14.7	*cis*	59
5	*Lactobacillus kefiri*	Q6WVP7	11	81.9	*cis*	88
6	*Lactobacillus hilgardii*	A0A6G9Q1W5	-	4.5	*cis*	48
7	*Weissella thailandensis*	G0UH95	-	1.9	*cis*	43
8	*Lactobacillus composti*	WP_035452573.1	-	0.7	*cis*	13
9	*Leuconostoc carnosum*	K0DAB2	-	45.8	*cis*	22
10	*Weissella confusa*	A0A5E8FA41	-	1.2	*cis*	41
11	*Pediococcus acidilactici*	K9IC06	-	0.7	*cis*	29
12	*Lactobacillus otakiensis*	S4NNE4	-	1.5	*cis*	63
13	*Lactobacillus* sp.	A0A3R8GRD4	-	0.5	*cis*	6
14	*Scardovia inopinata*	W5IGT2	-	2.4	*cis*	51
15	*Lactobacillus reuteri*	KEK13971.1	-	53.7	*cis*	97
16	*Acetobacter pomorum*	F1YWW2	-	2.7	*cis*	81
17	*Acetobacter pasteurianus*	S6D646	12	0.8	*cis*	46
18	*Acetobacter tropicalis*	F7VE72	-	3.8	*cis*	86
19	*Rhodococcus qingshengii*	WP_007734806.1	-	11.9	*cis*	96
20	*Methylibium* sp.	W7WM99	-	8.1	*cis*	94
21	*Weissella hellenica*	A0A4Y4G9L8	-	0.0	-	-
22	*Weissella* sp.	WP_042491976.1	-	0.2	*cis*	100
23	*Bacillus subtilis*	G4EPZ5	13	0.9	*trans*	9
24	*Debaryomyces hansenii*	Q6BQ25	14	0.6	*cis*	4
25	*Candida parapsilosis*	B2KJ46	15	0.8	*trans*	12
26	*Candida magnoliae*	Q9C4B3	16	0.7	*cis*	24
27	*Weissella hellenica*	A0A4Y4G1R6	-	0.0	-	-
28	*Pseudomonas tolaasii*	WP_016970017.1	-	0.3	*trans*	100
29	*Weissella hellenica*	A0A5B8TNR6	-	0.0	-	-
30	*Candida glabrata*	Q6FR42	17	5.0	*cis*	96
31	*Saccharomyces cerevisiae S288C*	P14065	18	1.3	*cis*	46
32	*Saccharomyces cerevisiae* RM11-1a	B3LN10	19	4.9	*cis*	76
33	*Aerococcus viridans*	D4YGD0	-	0.4	*cis*	20
34	*Microbacterium luteolum*	Q76KC2	20	6.2	*cis*	74
35	*Aspergillus oryzae* RIB40	Q2UMR5	-	0.3	*trans*	56
36	*Rhodococcus* sp.	A0A1Q8I6M1	21	0.4	*cis*	16
37	*Rhodococcus erythropolis*	Q6YBW1	22	0.4	*cis*	14
38	*Candida parapsilosis*	A1X808	23	0.4	*cis*	20
39	*Thermoanaerobacter ethanolicus*	Q2MJT8	24	1.3	*trans*	19
40	*Pseudomonas tolaasii*	WP_016974539.1	-	0.2	*trans*	100
41	*Ogataea glucozyma*	A0A0H4SN47	25	65.0	*cis*	98
**42**	***Vitis vinifera***	**P93799**	26	**3.2**	***trans***	**68**
43	*Aspergillus oryzae* RIB40	Q2UJ34	-	3.7	*cis*	94
44[b]	*Pseudomonas tolaasii*	WP_080520040.1	-	43.2	*cis*	100
45	*Streptomyces cyanogenus*	Q9ZGC1	27	47.1	*cis*	99
46	*Curvularia lunata*	3IS3_A	28	3.4	*cis*	77
47	*Magnaporthe grisea*	Q9HFV6	29	1.1	*cis*	82
48[b]	*Pseudomonas tolaasii*	WP_016973621.1	-	8.9	*cis*	53
49	*Pseudomonas fluorescens*	A0A1Q5WM56	-	2.7	*cis*	40
50	*Pseudomonas tolaasii*	WP_016973456.1	-	28.7	*cis*	41
51	*Weissella hellenica*	A0A4Y4G167	-	0.0	-	-

Supplementary Table 1 (continued). Activity and selectivity of the supplemented in-house KRED collection[a] towards **1a**.

KRED	Source organism	Protein ID	retrieved from Ref.	Conversion [%]	selectivity	*de* [%]
52	*Weissella minor*	KRN76072.1	-	0.4	*cis*	61
53	*Novosphingobium aromaticivorans*	WP_011906790.1	-	0.3	*cis*	58
**54**	***Sporidiobolus salmonicolor***	**Q9UUN9**	30,31	**92.1**	***trans***	**99**
55	*Thermoanaerobacter pseudethanolicus*	ABY93890.1	-	0.4	*cis*	17
56	*Nocardioides* sp. S5	WP_032491529.1	-	2.1	*cis*	73
57	*Lactobacillus helveticus*	A0A2V4EQM7	-	0.6	*cis*	10
58	*Devosia riboflavina*	A0A087LNW6	-	0.3	*cis*	58
59	*Pseudomonas tolaasii*	WP_016969242.1	-	0.6	*cis*	35
60	*Aspergillus oryzae*	OOO13522.1	-	0.4	*cis*	47
61	*Aspergillus oryzae* RIB40	XP_001819099.1	-	0.2	*trans*	100
62	*Aspergillus oryzae* RIB40	XP_001821943.1	-	0.4	*cis*	62
63	*Scedosporium apiospermum*	XP_016641805.1	-	0.2	*trans*	100
	EV	-	-	0.5	*cis*	1

EV, empty vector pET22b(+). Top KRED candidates are highlighted in bold letters.

[a] The in-house KRED collection (KRED 1 – 51)[1] was supplemented with 12 new KRED candidates (52 – 63).

[b] In contrast to Voss, Küng *et al*., 2021,[1] KRED 44 and KRED 48 were herein produced in their original (non-truncated) version.

Reactions were performed with *E. coli* crude cell extracts and 3 g L^{-1} **1a**, equivalent to 10 mM **1a**, in the presence of NADH, NADPH and the glucose/glucose dehydrogenase system for cofactor regeneration (see **Supplementary Method 1.8**).

Supplementary Table 2. L1 hits evaluated as non-treated, heat- or solvent-treated lysates.

No. sites	No. beneficial mutations	AA	Mutation	FIOWT	FIOWT, 50 °C, 17 h	FIOWT, 20 % (v/v) iPrOH, 17 h
1	1	V144	R	1.6		
2	2	D150	N	2.1		
3	3	T164	I	1.6		
4	4	L174	T	1.2	n.d.	n.d.
	5		V	1.2	n.d.	n.d.
	6		M	1.2	n.d.	n.d.
5	7	N202	V	1.6		
	8		H	1.3		
6	9	Y208	F	1.6	n.d.	n.d.
7	10	P216	Q	1.4		
8	11	M228	K	1.4		
	12		Q	1.3		
	13		R	1.3		
	14		N	1.4		
9	15	E234	D	1.4		
	16		C	1.4		
10	17	A238	Y	1.3		
	18		K	1.2	n.d.	n.d.
	19		H	1.5		
11	20	M242	F	2.2		
	21		A	1.4		
12	22	Y246	M	1.7		
	23		S	1.8		
13	24	G254	A	1.6		
14	25	L258	C	1.7		
	26		F	1.5		
	27		A	1.3		
	28		V	1.4		
15	29	C260	G	1.6		
16	30	V262	C	2.2		
17	31	I266	T	1.3		
	32		Y	1.4		
18	33	T274	V	1.3		
19	34	G276	R	1.3		
	35		A	1.4		
	36		H	1.4		
20	37	F278	I	1.4		
	38		S	1.3		
21	39	A298	T	1.3		
22	40	T312	F	1.3		
23	41	L316	M	1.6		
24	42	I318	Q	1.4		
25	43	R324	I	1.4		
	44		V	1.5		
	45		S	1.3		
26	46	G326	I	2.1		
	47		V	1.6		
	48		P	1.7		
27	49	T342	M	1.9		

(50 °C)	(20 % iPrOH)
≥ 0.6	≥ 0.65
0.1 – 0.59	< 0.65
< 0.1	

The mutational scanning library contained SSM libraries on every second amino acid position (171 sites). Approximately 7700 transformants (76 % library coverage) were screened and variants with improved performance were sequenced.

The library was cultivated under different conditions compared to the rest (see **Supplementary Method 1.4**).

The table contains only mutants verified in at least two independent experiments. FIOWT values were estimated by HPLC (threshold value: 1.2). L1 revealed 27 positions in the substrate environment, second sphere and protein surface as having a positive impact on reductase performance. Notably, L1-investigated residues L174, A238 and M242 also appeared as L2 hot spots.

Surface residue positions V144, D150, L316, G326, T342 were selected for L8.

n.d., not determined.

Supplementary Table 3. L2 variants and their FIOWT values[a] (average of duplicates).

AA	Amino acid position					
	F97	L174	A238	L241	M242	Q245
**A**	0.08	1.29	**1.00**	1.02	0.89	0.58
**C**	0.39	0.40	0.80	0.96	1.02	1.12
**D**	0.02	0.00	0.66	0.88	0.00	0.85
**E**	0.00	0.05	0.69	0.71	0.04	1.14
**F**	**1.00**	0.69	1.28	1.00	2.63	0.29
**G**	0.30	0.61	1.07	0.84	0.21	0.04
**H**	0.01	0.28	1.41	0.98	0.14	1.38
**I**	0.00	1.04	1.20	1.16	1.22	0.25
**K**	0.00	0.51	2.11	1.20	0.15	0.45
**L**	0.67	**1.00**	0.44	**1.00**	0.90	0.29
**M**	0.76	0.96	1.71	1.22	**1.00**	0.47
**N**	0.00	0.38	1.52	1.14	0.20	2.35
**P**	0.05	0.45	1.13	0.60	0.17	0.15
**Q**	0.05	0.49	1.87	1.02	0.26	**1.00**
**R**	0.05	0.41	1.94	1.20	0.29	0.33
**S**	0.00	0.44	0.96	0.81	0.75	1.35
**T**	0.00	1.01	1.03	0.82	0.85	3.55
**V**	0.00	1.09	0.92	0.92	0.45	0.11
**W**	1.41	0.84	1.02	1.20	1.66	0.69
**Y**	0.03	1.19	1.72	0.92	0.47	0.74

[a] UV assays were performed using 3 % (v/v) lysate.

Supplementary Table 4. L3 – L6 variants with highest FIOWT values within each library.[a] All data is provided in https://github.com/ccbiozhaw/Ssal-KRED_evolution.

Lib.	No.	Plate_Well	F97	L174	A238	L241	M242	Q245	FIOWT
L3	1	L3_ML010_A09	W	N	A	L	M	T	2.7
	2	L3_ML003_A03	W	S	A	L	M	N	2.3
	3	L3_ML001_B05	W	L	A	L	P	T	2.1
	4	L3_ML006_G02	W	L	A	L	A	T	1.9
	5	L3_ML009_E08	W	Q	A	L	A	T	1.9
	6	L3_ML001_H08	W	S	A	L	M	M	1.8
**L4**	**1**	**L4_ML006_G10**	**W**	**L**	**A**	**M**	**W**	**S**	**5.0**
	2	L4_ML010_E09	W	L	A	C	W	S	4.8
	3	L4_ML010_H04	W	L	A	Y	W	S	4.3
	4	L4_ML008_H02	W	L	A	V	Y	T	3.4
	5	L4_ML002_E05	W	L	A	Q	M	N	2.9
	6	L4_ML005_C09	W	L	A	W	V	W	2.7
	7	L4_ML006_D11	W	L	A	F	Y	T	2.6
	8	L4_ML010_C06	W	L	A	H	F	S	2.4
	9	L4_ML005_C07	W	L	A	V	M	T	2.4
	10	L4_ML010_C02	W	L	A	L	F	C	1.9
	11	L4_ML001_H08	W	L	A	V	F	S	1.7
	12	L4_ML006_G01	W	L	A	V	M	W	1.6
	13	L4_ML010_C09	W	L	A	I	M	C	1.6
L5	1	L5_ML001_C07	W	L	K	N	I	Q	3.0
	2	L5_ML008_G12	W	L	N	R	L	Q	2.7
	3	L5_ML009_F08	W	L	K	K	V	Q	2.6
	4	L5_ML001_A12	W	L	L	L	M	Q	2.6
	5	L5_ML001_B06	W	L	H	H	I	Q	2.4
	6	L5_ML007_H08	W	L	K	M	M	Q	2.0
	7	L5_ML007_H06	W	L	K	M	V	Q	1.8
	8	L5_ML010_F05	W	L	N	K	I	Q	1.7
	9	L5_ML006_F12	W	L	L	M	M	Q	1.4
	10	L5_ML006_G05	W	L	K	K	A	Q	1.3
L6	1	L6_ML002_B08	W	T	Y	W	M	Q	2.8
	2	L6_ML011_B11	W	V	Y	L	M	Q	2.8
	3	L6_ML008_H02	W	M	H	R	M	Q	2.4
	4	L6_ML003_F09	W	M	L	R	M	Q	2.4
	5	L6_ML002_B04	W	M	K	K	M	Q	2.4
	6	L6_ML002_C01	W	V	Q	H	M	Q	2.3
	7	L6_ML006_E04	W	Y	K	N	M	Q	2.2
	8	L6_ML004_B03	W	A	L	M	M	Q	2.1
	9	L6_ML004_B01	W	M	Q	N	M	Q	2.1
	10	L6_ML012_B08	W	I	N	L	M	Q	2.1
	11	L6_ML004_G06	W	T	R	M	M	Q	2.0
	12	L6_ML006_F11	W	L	K	L	M	Q	2.0
	13	L6_ML009_H02	W	M	K	L	M	Q	2.0
	14	L6_ML011_D06	W	K	Y	Q	M	Q	2.0
	15	L6_ML006_C05	W	V	R	N	M	Q	2.0
	16	L6_ML011_A09	W	T	L	Y	M	Q	2.0
	17	L6_ML002_D02	W	T	R	Q	M	Q	1.9
	18	L6_ML010_D08	W	M	H	S	M	Q	1.9
	19	L6_ML002_D10	W	V	T	L	M	Q	1.8
	20	L6_ML002_E08	W	T	H	H	M	Q	1.8
	21	L6_ML002_H06	W	V	T	M	M	Q	1.8
	22	L6_ML005_C07	W	T	Q	L	M	Q	1.8
	23	L6_ML012_B11	W	V	S	L	M	Q	1.8
	24	L6_ML009_B03	W	L	L	M	M	Q	1.8
	25	L6_ML003_G03	W	T	K	M	M	Q	1.8
	26	L6_ML004_B09	W	L	R	V	M	Q	1.8
	27	L6_ML002_F08	W	R	H	R	M	Q	1.8
	28	L6_ML003_B06	W	M	Q	Q	M	Q	1.8

[a] UV assays were performed using 3 % (v/v) lysate.

Supplementary Table 5. L7 variants with highest FIOWT values.[a] All data is provided in https://github.com/ccbiozhaw/Ssal-KRED_evolution.

No.	Plate_Well	F97	L174	A238	L241	M242	Q245	FIOWT
1	L7_ML011_F04	W	I	K	H	W	D	3.4
2	L7_ML011_C07	W	M	R	Q	M	S	3.2
3	L7_ML017_B04	W	L	R	G	M	W	3.1
4	L7_ML011_E04	W	W	K	D	I	F	2.9
5	L7_ML017_C07	W	M	N	K	M	T	2.8
6	L7_ML014_A09	W	I	W	S	P	N	2.7
7	L7_ML007_E03	W	L	L	T	F	C	2.6
8	L7_ML002_E04	W	V	L	Y	W	S	2.4
9	L7_ML015_A03	W	I	G	C	Y	T	2.4
10	L7_ML011_H04	W	M	D	A	M	T	2.3
11	L7_ML005_C08	W	M	R	Y	M	F	2.2
12	L7_ML002_F05	W	Q	D	R	I	M	2.0
13	L7_ML018_F10	W	C	N	N	W	S	2.0
14	L7_ML014_F08	W	I	G	M	W	H	2.1
15	L7_ML015_C08	W	M	R	M	F	S	2.1
16	L7_ML008_F04	W	I	H	L	M	S	1.9
17	L7_ML006_F06	W	T	T	Q	M	N	1.9
18	L7_ML010_H08	W	I	W	S	P	N	1.8
19	L7_ML001_A05	W	G	L	A	M	M	1.6
20	L7_ML001_H10	W	K	G	T	C	T	1.6

[a] UV assays were performed using 3 % (v/v) lysate.

Supplementary Table 6. FIOWT values and selectivity of the wild type and M1 under optimized UV assay conditions (measured as quadruplicates).

Variant name	6-site variant code[a] (97, 241, 242, 245, 316, 342)	FIOWT[b]	*de* [area %] (*trans*)
WT	F L M Q L T	1.0	> 99.9
M1	W M W S L T	8.3 ± 0.4	> 99.9

[a] Surface residues are underlined.
[b] UV assays were carried out with 1 % (v/v) lysate.

Supplementary Table 7. List of variants selected for ML-based library L9.

Variant No.	ML prediction ranking	ML-predicted variant[a] (positions 97, 241, 242, 245)
1	1	W T W T
2	2	W V W T
3	3	W S W T
4	4	W I W T
5	5	W C W T
6	6	W Y W T
7	7	W A W T
8	8	W Q W T
9	9	W F W T
10	10	W T W S
11	11	W S W S
12	12	W N W T
13	13	W V W S
14	14	W R W T
15	15	W H W T
16	16	W G W T
17	17	W M W T
18	18	W K W T
19	19	W I W S
20	20	W D W T
21	21	W L W T
22	22	W W W T
23	23	W A W S
24	24	W Q W S
25	29	W I Y T
26	31	W T F T
27	32	W V F T
28	35	W S F T
29	44	W M M N
30	50	W C W N

[a] Mutation F97W was kept fixed. ML applied on positions 241, 242 and 245.

Supplementary Table 8. FIOWT and selectivity of top 10 hits of ML-based library L9 (average of duplicates).

Variant abbreviation	4-site variant code (97, 241, 242, 245)	FIOWT 1.5 % (v/v) lysate[a]	*de* [area %] (*trans*)	
			3 mM 1a (1 h)	7.5 mM 1a (1 h)
**WT**	F L M Q	1.0	> 99.9	> 99.9
**Q245T (top L2)**	F L M T	4.8	> 99.9	99.7[b]
**WMWS (top L4 = M1)**	W M W S	8.3	> 99.9	> 99.9
**WQWS**	W Q W S	8.0	> 99.9	> 99.9
**WSWS**	W S W S	7.4	> 99.9	> 99.9
**WAWS**	W A W S	7.2	> 99.9	> 99.9
**WTWS**	W T W S	6.1	> 99.9	> 99.9
**WVWS**	W V W S	5.4	> 99.9	> 99.9
**WLWT**	W L W T	4.7	> 99.9	> 99.9
**WCWT**	W C W T	4.1	> 99.9	> 99.9
**WQWT**	W Q W T	3.7	> 99.9	> 99.9
**WTFT**	W T F T	3.3	n.d.	n.d.
**WAWT**	W A W T	3.2	n.d.	n.d.

n.d., not determined.

[a] Screening of L2 – L8 was carried out using 3 % (v/v) lysate. Starting from L9, screening was measured at ≤ 1.5 % (v/v) lysate as it was more accurate for FIOWT > 4.

[b] HPLC-UV chromatogram in **Supplementary Figure 4**.

Supplementary Table 9. L8 variants and their FIOWT values (average of duplicates).[a]

No.	Variant name	Substitutions					FIOWT	Retained activity vs. non treated [%]	
		V144	D150	L316	G326	T342		50 °C, 30 min	20 % (v/v) iPrOH, 2 h
	negative control	n.a.	n.a.	n.a.	n.a.	n.a.	0.10	n.a.	n.a.
	WT (VDLGT)	-	-	-	-	-	1.00	87	99
1	RDLGT	R	-	-	-	-	1.03	–	–
2	VNLGT	-	N	-	-	-	1.00	–	–
3	VDMGT	-	-	M	-	-	1.06	–	–
4	VDLIT	-	-	-	I	-	1.22	–	–
5	VDLGM	-	-	-	-	M	1.10	–	–
6	RNLGT	R	N	-	-	-	1.12	–	–
7	RDMGT	R	-	M	-	-	1.19	–	–
8	RDLIT	R	-	-	I	-	1.28	–	–
9	RDLGM	R	-	-	-	M	0.99	–	–
10	VNMGT	-	N	M	-	-	1.07	–	–
11	VNLIT	-	N	-	I	-	1.17	–	–
12	VNLGM	-	N	-	-	M	0.97	–	–
13	VDMIT	-	-	M	I	-	1.35	32	104
14	VDMGM	-	-	M	-	M	1.29	–	–
15	VDLIM	-	-	-	I	M	1.11	–	–
16	RNMGT	R	N	M	-	-	1.29	–	–
17	RNLIT	R	N	-	I	-	1.19	–	–
18	RNLGM	R	N	-	-	M	0.95	–	–
19	RDMIT	R	-	M	I	-	1.51	34	106
20	RDMGM	R	-	M	-	M	1.20	–	–
21	RDLIM	R	-	-	I	M	1.19	–	–
22	VNMIT	-	N	M	I	-	1.24	–	–
23	VNMGM	-	N	M	-	M	1.25	–	–
24	VNLIM	-	N	-	I	M	1.02	–	–
25	VDMIM	-	-	M	I	M	1.35	35	92
26	RNMIT	R	N	M	I	-	1.23	–	–
27	RNMGM	R	N	M	-	M	1.24	–	–
28	RNLIM	R	N	-	I	M	0.90	–	–
29	RDMIM	R	-	M	I	M	1.37	48	69
30	VNMIM	-	N	M	I	M	1.24	–	–
31	RNMIM	R	N	M	I	M	1.32	8	63

Focused combinatorial library L8 targeted surface residue positions identified in the mutational scanning library L1. Positions V144, D150, L316, G326 and T342 were substituted with only two amino acids: the native amino acid and the beneficial mutation found in the mutational scanning library L1 (**Supplementary Table 2**). Therefore, the sequence space is 2^5 = 32 variants – 1 (wildtype) = 31 variants. This library was investigated in the context of the wild-type enzyme. Lysates derived from the top 5 performing variants (all containing mutation G326I) were subjected to heat and organic solvent treatment prior to the UV assay; however, all of them displayed decreased thermal stability. Consequently, the best combination lacking G326I (VDMGM) was considered for studies in the context of more evolved variants.

-, unchanged residue; –, not determined; n.a., not applicable.

[a] UV assays were performed using 3 % (v/v) lysate.

Supplementary Table 10. FIOWT values of substrate- and surface environment combination variants as well as controls (average of triplicates).

Parent	4-site variant code (97, 241, 242, 245)	FIOWT[a] No surface residue mutation	FIOWT[a] + VDMGM (L316M/T342M)	Increase in FIOWT + VDMGM (L316M/T342M)
WT	F L M Q	1.0	1.4	↑ 38 %
Q245T	F L M T	3.8	5.1	↑ 33 %
WMWS	W M W S	8.0	8.5	↑ 6 %
WCWS	W C W S	5.8	6.6	↑ 14 %
WYWS	W Y W S	5.1	5.6	↑ 9 %
WVYT	W V Y T	4.2	4.9	↑ 17 %

Substrate- and surface environment mutations were selected from the best L4 variants (**Supplementary Table 4**) and selected L8 combination (**Supplementary Table 9**), respectively.
[a] UV assays were carried out with 1.5 % (v/v) lysate.

Supplementary Table 11. FIOWT values and selectivity of M2 under optimized UV assay conditions (measured as quadruplicates).

Variant name	6-site variant code[a] (97, 241, 242, 245, 316, 342)	FIOWT[b]	*de* [area %] (*trans*)
WT	F L M Q L T	1.0	> 99.9
M2	W M W S M M	9.0 ± 0.4	> 99.9

[a] Surface residues are underlined.

[b] UV assays were carried out with 1 % (v/v) lysate.

Supplementary Table 12. Selected substrate- and surface-environment hit combination variants and their retained activity after heat or solvent treatment (average of triplicates).

Variant abbreviation	8-site variant code[a] (97, 144, 150, 241, 242, 245, 316, 326, 342)	Retained activity[b]	
		50 °C, 1 h	20 % (v/v) iPrOH, 1 h
WT	F V D L M Q L G T	89 %	105 %
WMWS (M1)	W V D M W S L G T	86 %	108 %
WMWS + VDMGM (M2)	W V D M W S M G M	86 %	103 %

[a] Surface residues are underlined.

[b] The retained activity was measured using the UV assay at 1 % (v/v) lysate. Activity of non-treated controls is equivalent to 100 % in each case.

Supplementary Table 13. ML-filtered library L10. Selected positions and proposed amino acids for combinatorial mutagenesis.

Environment	Position No.	Residue in WT	Proposed residues	Fixed or variable	No. substitutions
Substrate	97	F	W	fixed	1
	174	L	A, M, L, V, I	variable	5
	238	A	L, R, K, G, N	variable	5
	241	L	M, Q, S	variable	3
	242	M	W	fixed	1
	245	Q	S	fixed	1
Surface	316	L	M	fixed	1
	342	T	M	fixed	1

Pos. 174: top 50 predictions only included residues L and M. Residues A, V and I were selected because they performed well in SSM library L4 and, similarly to residues L and M, due to their hydrophobicity.

Pos. 238: top 50 predictions included residues L, R, K and G. Asn performed well individually (L2) and showed up in the top 200 predicted variants.

Pos. 241: Residues M, Q, S were found in the best variants of ML-derived L9. Although A was good in predictions, it was not considered, as the top 3 variants of L4 and L9 did not contain it.

Pos. 242: Fixed because it was the most beneficial in L4/L9.

Pos. 245: Fixed because it was the most beneficial in L4/L9.

Supplementary Table 14. Hit variants of filtered ML-based library L10 (measured as quadruplicates).

Variant name	3-site variant code (174, 238, 241)	FIOP[a] (vs. M2)	FIOWT[b]
**M3**	**L K M**	**2.5 ± 0.1**	**22.2**
L10_P4_D01	L G M	1.9 ± 0.1	17.3
L10_P1_F05	L K Q	1.8 ± 0.1	16.0
L10_P2_A07	L R M	1.7 ± 0.1	15.3
L10_P3_D08	L K S	1.7 ± 0.0	15.1
L10_P4_C07	L L M	1.3 ± 0.1	11.7
L10_P2_A05	L N Q	1.3 ± 0.1	11.4
L10_P1_H05	I K M	1.2 ± 0.1	10.6
L10_P1_D08	L R Q	1.2 ± 0.0	10.6
M2	L A M	1.0 ± 0.1	9.0[b]
M1	L A M	0.9 ± 0.1	8.5[b]

All L10 variants additionally contain parental mutations F97W, L241M, M242W, Q245S, L316M and T342M (M2).

[a] UV assays were carried out with 0.25 % (v/v) lysate.

[b] Values estimated from FIOP.

Supplementary Table 15. L11 hit variants (average of triplicates).

Variant No.	Description	FIOP (vs. M3)[a]	FIOWT[b]
**M3 + Y246G (M4)**	**Y246G**	**1.10**	**24.4**
M3 + Y246K	Y246K	1.08	24.0

[a] UV assays were carried out with 0.25 % (v/v) lysate.

[b] Values estimated from FIOP.

Supplementary Table 16. L12 top variant.

Targeted sites	No. of variants FIOP ≥ 1 (vs. M4)[a]	Best FIOP (vs. M4)[a]	Observation	Sequencing results of variants with FIOP ≥ 1 (vs. M4)[a]
P206X	2	1.02	Low number of transformants	Silent mutations in all variants
N207X	1	1.00	-	Silent mutations in all variants
Y208X	0	-	-	-
T209X	5 -7	< 1.20	-	Silent mutations in all variants
S222X	19	< 1.20	-	Silent mutations in all variants
T223X	10	< 1.17	-	Silent mutations in all variants
S224X	15	1.20	-	**S224A (FIOP = 1.2) → M5**
W226X	3	< 1.18	-	Silent mutations in all variants

[a] UV assays were carried out with 0.25 % (v/v) lysate.

Supplementary Table 17. Retained activity of heat-treated lysates derived from L1 – L12 hit variants (average of duplicates).

Library	Best variant	Retained activity[a] [%], (50 °C, 1 h)
L1 – L8	M2	89
L9, L10	M3	95
L11	M4	92
L12	M5	98

[a] UV assays were carried out with 0.5 % (v/v) lysate. The activity of non-treated lysate of each variant was normalized to 100 %.

Supplementary Table 18. L13 top variants (average of quadriplicates).

Variant abbreviation	T134	V135	FIOP (vs. M5)[a]	FIOWT[b]
**M5 + VV (M6)**	**V**	**V***	**2.0**	**58.0**
M5 + VC	V	C	1.5	43.5
M5 + VT	V	T	1.5	43.5
M5 + CV	C	V	1.3	37.7
M5 + AV	A	V	1.2	34.8
M5 + QV	Q	V*	1.1	31.9
M5 + MV	M	V	1.1	31.9

*silent mutation (V135: GTT instead of original GTG).

[a] UV assays were carried out with 0.25 % (v/v) lysate.

[b] Values estimated from FIOP.

Supplementary Table 19. Summary of rationale for the selection of *Ssal*-KRED hot spots investigated in libraries L2 – L13.

No.	Pos.	Environment / Interaction	Library (L)	Amino acid mutations in library	Substrate binding mode model		Exp. Data (L1)		Literature-based	
					proposed mutation	Effector	mutation	FIOWT	(binding) role	mutagen-esis
1	F97	subs. Tunnel entrance	2 – 7, 9, 10	all	–	–	–	–	[30]	–
2	L174	substrate	2 – 7, 10	all	–	–	T, V, M	1.2	[30,32]	–
3	A238	subs. Tunnel entrance	2 – 7, 10	all	–	–	Y, K, H	1.2 – 1.5	[30]	–
4	L241	subs. Tunnel entrance	2 – 7, 9, 10	all	–	–	–	–	[30]	–
5	M242	substrate	2 – 7, 9, 10	all	–	–	F, A	1.4 – 2.2	[33]	[33]
6	Q245	substrate	2 – 7, 9, 10	all	–	–	–	–	[32–34]	[32–34]
7	V144	surface	8	V, R	–	–	R	1.6	–	–
8	D150	surface	8	D, N	–	–	N	2.1	–	–
9	L316	surface	8	L, M	–	–	M	1.6	–	–
10	G326	surface	8	G, I	–	–	I	2.1	–	–
11	T342	surface	8	T, M	–	–	M	1.9	–	–
12	P243	substrate, 242, 245	11	all	–	–	–	–	[32]	[32]
13	Y246	245	11	all	–	–	S, M	1.7 – 1.8	–	–
14	P206	NAPDH_C4	12	all	L, I	[a]	–	–	[30]	–
15	N207	NADPH_C4	12	all	n.p.g.	[b]	–	–	[35]	[35]
16	Y208	substrate	12	all	n.p.g.	[a]	F	1.6	[35]	[35]
17	T209	NADPH_C4, substrate	12	all	I	[a]	–	–	[35]	[35]
18	S222	NADPH, substrate	12	all	–	–	–	–	[35]	[35]
19	T223	NADPH_C4, substrate	12	all	–	–	–	–	[35]	[35]
20	S224	substrate	12	all	–	–	–	–	[35]	[35]
21	W226	subs. Tunnel entrance	12	all	–	–	–	–	[30,35]	–
22	T134	substrate	13	all	–	–	–	–	[32]	[32]
23	V135	substrate	13	all	–	–	–	–	[32]	[32]

–, not explored, not determined; n.p.g., not particularly given

NADPH_C4, hydride of C4 from the nicotinamide ring of NADPH.

[a] Potential for improved hydrophobic contacts.

[b] Potential for improved polar interactions.

Supplementary Table 20. Summary of libraries generated in the *Ssal*-KRED engineering campaign.

Lib. No.	Library type	Parent	Environ-ment	Targeted positions in parent	Construct-ion method (CM)	Library size (*L*) according to CM	Trans-formants screened (*T*)	Theo-retical No. UOT variants	UOT variants screen-ed	Screen-ing cov-erage[b] [%]
L1	mutational scanning	WT	whole protein	171 even-numbered positions	NNK	5,472	7,695	3,249	2,657[c]	75.5
L2	SSM	WT	substrate	F97	22c-trick	22	71	20	19[d]	100
			substrate	L174	22c-trick	22	86	20	19[d]	100
			substrate	A238	22c-trick	22	70	20	19[d]	100
			substrate	L241	22c-trick	22	103	20	19[d]	100
			substrate	M242	22c-trick	22	110	20	19[d]	100
			substrate	Q245	22c-trick	22	110	20	19[d]	100
L3	3-site CSM	F97W	substrate	L174, M242, Q245	22c-trick	10,648	450	8,000	450[d]	5.6
L4	3-site CSM	F97W	substrate	L241, M242, Q245	22c-trick	10,648	680	8,000	680[d]	8.5
L5	3-site CSM	F97W	substrate	A238, L241, M242	22c-trick	10,648	440	8,000	440[d]	5.5
L6	3-site CSM	F97W	substrate	L174, A238, L241	22c-trick	10,648	563	8,000	563[d]	7.0
L7	5-site CSM	F97W	substrate	L174, A238, L241, M242, Q245	Twist	3‘200,000	762	3‘200,000	762[d]	0.024
L8[a]	focused combinatorial	WT	surface	V144, D150, L316, G326, T342	nd-MP	31	31	31	31[d]	100
	hit combination	M1	substrate + surface	V144, L316, G326, T342	nd-MP	10	10	10	10[d]	100
L9	L4-derived ML	F97W	substrate	L241, M242, Q245	22c-trick, d-/nd-MP	33	118	30	28[d]	93.3
L10	L2 – L7-derived filtered ML	M2	substrate	L174, A238, L241	d-/nd-MP	75	344	75	74[c]	98.9
L11	2-site CSM	M3	substrate	P243, Y246	22c-trick	484	1,424	400	379[c]	94.7
L12	SSM	M4	NADPH	P206	NNK	32	178	20	19[c]	99.6
		M4	NADPH, substrate	N207	NNK	32	178	20	19[c]	99.6
		M4	NADPH, substrate	Y208	NNK	32	178	20	19[c]	99.6
		M4	NADPH	T209	NNK	32	178	20	19[c]	99.6
		M4	substrate	S222	NNK	32	178	20	19[c]	99.6
		M4	NADPH, substrate	T223	NNK	32	178	20	19[c]	99.6
		M4	substrate	S224	NNK	32	178	20	19[c]	99.6
		M4	substrate	W226	NNK	32	178	20	19[c]	99.6
L13	2-site CSM	M5	substrate	T134, V135	22c-trick	484	534	400	267[c]	66.7
Total						3‘249,569	15,025	3‘236,475	**6,607**[c]	

SSM, single-site saturation mutagenesis; CSM, combinatorial saturation mutagenesis; d- or nd-MP, degenerate or non-degenerate mutagenic primers; UOT, unique on-target variants.

[a] L8 variants were individually generated with nd-MP, verified by sequencing, and screened (no oversampling needed).

[b] Upon sequencing of a complete library or a fraction of it, the screening coverage was calculated as: UOT variants screened / theoretical No. UOT variants. Upon sequencing of hits only, the screening coverage was calculated as a probability with: $T = - L \cdot \ln(1 - F)$, where L = Library size, T = transformants screened and F = fractional library completeness.[2] [c] estimated number of UOT variants screened (calculated from screening coverage, as only hits were sequenced). [d] real number of UOT variants screened (all verified by sequencing). Screening was conducted via the UV assay.

Supplementary Table 21. Oligonucleotides used in this study.

Application	Name	Sequence (5'→3')
Sequencing	Seq_KP104_XmaI-fw	GTG GTT GAG CAG CTG CTG GAA CAC
In-Fusion flanking primers (¥also available as desalted)	InF_NDT_fw	GGT GCT GGT TAC CGG TGC GAA CGG TTT TGT GGC G
	InF_XmaI_fw_PAGE¥	ACG CGA AAT ACC CGG GTC GTT TTG AGA CCG CGG TGG
	InF_NDT_rev_PAGE¥	GCT CGA ATT CGG ATC CTT ACG CGG TCT CGC TGC C
	InF_T342M_BamHI_rev_PAGE	GCT CGA ATT CGG ATC CTT ACG CCA TCT CGC TGC C
L2	F97_NDT_fw	CG AGC GTG GTT AGC NDT AGC AAC AAG TAT GAT
	F97_NDT_rv	ATC ATA CTT GTT GCT AHN GCT AAC CAC GCT CG
	F97_VHG_fw	CG AGC GTG GTT AGC VHG AGC AAC AAG TAT GAT
	F97_VHG_rv	ATC ATA CTT GTT GCT CDB GCT AAC CAC GCT CG
	F97_TGG_fw	CG AGC GTG GTT AGC TGG AGC AAC AAG TAT GAT
	F97_TGG_rv	ATC ATA CTT GTT GCT CCA GCT AAC CAC GCT CG
	L174_NDT_fw	AT CCG CAA AAG AGC NDT TGG GTG TAT GCG GCG A
	L174_NDT_rv	T CGC CGC ATA CAC CCA AHN GCT CTT TTG CGG AT
	L174_VHG_fw	AT CCG CAA AAG AGC VHG TGG GTG TAT GCG GCG A
	L174_VHG_rv	T CGC CGC ATA CAC CCA CDB GCT CTT TTG CGG AT
	L174_TGG_fw	AT CCG CAA AAG AGC TGG TGG GTG TAT GCG GCG A
	L174_TGG_rv	T CGC CGC ATA CAC CCA CCA GCT CTT TTG CGG AT
	A238_NDT_fw	TT AAC GGC GAG GTG TCT CCG NDT CTG GCG CTG ATG C
	A238_NDT_rv	G CAT CAG CGC CAG AHN CGG AGA CAC CTC GCC GTT AA
	A238_VHG_fw	TT AAC GGC GAG GTG TCT CCG VHG CTG GCG CTG ATG C
	A238_VHG_rv	G CAT CAG CGC CAG CDB CGG AGA CAC CTC GCC GTT AA
	A238_TGG_fw	TT AAC GGC GAG GTG TCT CCG TGG CTG GCG CTG ATG C
	A238_TGG_rv	G CAT CAG CGC CAG CCA CGG AGA CAC CTC GCC GTT AA
	L241_NDT_fw	T CCG GCG CTG GCG NDT ATG CCG CCG CAG TAC TAT
	L241_NDT_rv	ATA GTA CTG CGG CGG CAT AHN CGC CAG CGC CGG A
	L241_VHG_fw	T CCG GCG CTG GCG VHG ATG CCG CCG CAG TAC TAT
	L241_VHG_rv	ATA GTA CTG CGG CGG CAT CDB CGC CAG CGC CGG A
	L241_TGG_fw	T CCG GCG CTG GCG TGG ATG CCG CCG CAG TAC TAT
	L241_TGG_rv	ATA GTA CTG CGG CGG CAT CCA CGC CAG CGC CGG A
	M242_NDT_fw	T CCG GCG CTG GCG CTG NDT CCG CCG CAG TAC TAT
	M242_NDT_rv	ATA GTA CTG CGG CGG AHN CAG CGC CAG CGC CGG A
	M242_VHG_fw	T CCG GCG CTG GCG CTG VHG CCG CCG CAG TAC TAT
	M242_VHG_rv	ATA GTA CTG CGG CGG CDB CAG CGC CAG CGC CGG A
	M242_TGG_fw	T CCG GCG CTG GCG CTG TGG CCG CCG CAG TAC TAT
	M242_TGG_rv	ATA GTA CTG CGG CGG CCA CAG CGC CAG CGC CGG A
	Q245_NDT_fw	G CTG ATG CCG CCG NDT TAC TAT GTG AGC GCG GTT
	Q245_NDT_rv	AAC CGC GCT CAC ATA GTA AHN CGG CGG CAT CAG C
	Q245_VHG_fw	G CTG ATG CCG CCG VHG TAC TAT GTG AGC GCG GTT
	Q245_VHG_rv	AAC CGC GCT CAC ATA GTA CDB CGG CGG CAT CAG C
	Q245_TGG_fw	G CTG ATG CCG CCG TGG TAC TAT GTG AGC GCG GTT
	Q245_TGG_rv	AAC CGC GCT CAC ATA GTA CCA CGG CGG CAT CAG C
L3 (‡primers used also for L6)	Lib3_fw	ACT ATG TGA GCG CGG TTG ACA TCG GTC TGC TGC ACC
	L174_rev_NDT‡	CGC CGC ATA CAC CCA AHN GCT CTT TTG CGG
	L174_rev_VHG‡	CGC CGC ATA CAC CCA CDB GCT CTT TTG CGG
	L174_rev_TGG‡	CGC CGC ATA CAC CCA CCA GCT CTT TTG CGG
	L174down_F‡	GGG TGT ATG CGG CGA GCA AAA CCG AGG CGG
	mut_Lib3_MQ_P1_R	C CGC GCT CAC ATA GTA AHN CGG CGG AHN CAG CGC CAG CGC CGG AG
	mut_Lib3_MQ_P2_R	C CGC GCT CAC ATA GTA CDB CGG CGG CDB CAG CGC CAG CGC CGG AG
	mut_Lib3_MQ_P3_R	C CGC GCT CAC ATA GTA AHN CGG CGG CDB CAG CGC CAG CGC CGG AG
	mut_Lib3_MQ_P4_R	C CGC GCT CAC ATA GTA CDB CGG CGG AHN CAG CGC CAG CGC CGG AG
	mut_Lib3_MQ_P5_R	C CGC GCT CAC ATA GTA AHN CGG CGG CCA CAG CGC CAG CGC CGG AG
	mut_Lib3_MQ_P6_R	C CGC GCT CAC ATA GTA CCA CGG CGG AHN CAG CGC CAG CGC CGG AG
	mut_Lib3_MQ_P7_R	C CGC GCT CAC ATA GTA CDB CGG CGG CCA CAG CGC CAG CGC CGG AG
	mut_Lib3_MQ_P8_R	C CGC GCT CAC ATA GTA CCA CGG CGG CDB CAG CGC CAG CGC CGG AG
	mut_Lib3_MQ_P9_R	C CGC GCT CAC ATA GTA CCA CGG CGG CCA CAG CGC CAG CGC CGG AG

Supplementary Table 21 (continued). Oligonucleotides used in this study.

Application	Name	Sequence (5'→3')
L4	mut_Lib4-Prim1_R	CGC GCT CAC ATA GTA AHN CGG CGG AHN AHN CGC CAG CGC CGG AGA C
	mut_Lib4-Prim2_R	CGC GCT CAC ATA GTA AHN CGG CGG AHN CDB CGC CAG CGC CGG AGA C
	mut_Lib4-Prim3_R	CGC GCT CAC ATA GTA AHN CGG CGG CDB AHN CGC CAG CGC CGG AGA C
	mut_Lib4-Prim4_R	CGC GCT CAC ATA GTA CDB CGG CGG AHN AHN CGC CAG CGC CGG AGA C
	mut_Lib4-Prim5_R	CGC GCT CAC ATA GTA AHN CGG CGG CDB CDB CGC CAG CGC CGG AGA C
	mut_Lib4-Prim6_R	CGC GCT CAC ATA GTA CDB CGG CGG AHN CDB CGC CAG CGC CGG AGA C
	mut_Lib4-Prim7_R	CGC GCT CAC ATA GTA CDB CGG CGG CDB AHN CGC CAG CGC CGG AGA C
	mut_Lib4-Prim8_R	CGC GCT CAC ATA GTA CDB CGG CGG CDB CDB CGC CAG CGC CGG AGA C
	mut_Lib4-Prim9_R	CGC GCT CAC ATA GTA AHN CGG CGG AHN CCA CGC CAG CGC CGG AGA C
	mut_Lib4-Prim10_R	CGC GCT CAC ATA GTA AHN CGG CGG CCA AHN CGC CAG CGC CGG AGA C
	mut_Lib4-Prim11_R	CGC GCT CAC ATA GTA CCA CGG CGG AHN AHN CGC CAG CGC CGG AGA C
	mut_Lib4-Prim12_R	CGC GCT CAC ATA GTA AHN CGG CGG CDB CCA CGC CAG CGC CGG AGA C
	mut_Lib4-Prim13_R	CGC GCT CAC ATA GTA AHN CGG CGG CCA CDB CGC CAG CGC CGG AGA C
	mut_Lib4-Prim14_R	CGC GCT CAC ATA GTA CDB CGG CGG AHN CCA CGC CAG CGC CGG AGA C
	mut_Lib4-Prim15_R	CGC GCT CAC ATA GTA CDB CGG CGG CCA AHN CGC CAG CGC CGG AGA C
	mut_Lib4-Prim16_R	CGC GCT CAC ATA GTA CCA CGG CGG AHN CDB CGC CAG CGC CGG AGA C
	mut_Lib4-Prim17_R	CGC GCT CAC ATA GTA CCA CGG CGG CDB AHN CGC CAG CGC CGG AGA C
	mut_Lib4-Prim18_R	CGC GCT CAC ATA GTA CDB CGG CGG CDB CCA CGC CAG CGC CGG AGA C
	mut_Lib4-Prim19_R	CGC GCT CAC ATA GTA CDB CGG CGG CCA CDB CGC CAG CGC CGG AGA C
	mut_Lib4-Prim20_R	CGC GCT CAC ATA GTA CCA CGG CGG CDB CDB CGC CAG CGC CGG AGA C
	mut_Lib4-Prim21_R	CGC GCT CAC ATA GTA AHN CGG CGG CCA CCA CGC CAG CGC CGG AGA C
	mut_Lib4-Prim22_R	CGC GCT CAC ATA GTA CCA CGG CGG AHN CCA CGC CAG CGC CGG AGA C
	mut_Lib4-Prim23_R	CGC GCT CAC ATA GTA CCA CGG CGG CCA AHN CGC CAG CGC CGG AGA C
	mut_Lib4-Prim24_R	CGC GCT CAC ATA GTA CDB CGG CGG CCA CCA CGC CAG CGC CGG AGA C
	mut_Lib4-Prim25_R	CGC GCT CAC ATA GTA CCA CGG CGG CDB CCA CGC CAG CGC CGG AGA C
	mut_Lib4-Prim26_R	CGC GCT CAC ATA GTA CCA CGG CGG CCA CDB CGC CAG CGC CGG AGA C
	mut_Lib4-Prim27_R	CGC GCT CAC ATA GTA CCA CGG CGG CCA CCA CGC CAG CGC CGG AGA C
	mut_Lib4-Prim1_F	CT CCG GCG CTG GCG NDT NDT CCG CCG NDT TAC TAT GTG AGC GCG
	mut_Lib4-Prim2_F	CT CCG GCG CTG GCG NDT NDT CCG CCG VHG TAC TAT GTG AGC GCG
	mut_Lib4-Prim3_F	CT CCG GCG CTG GCG NDT VHG CCG CCG NDT TAC TAT GTG AGC GCG
	mut_Lib4-Prim4_F	CT CCG GCG CTG GCG VHG NDT CCG CCG NDT TAC TAT GTG AGC GCG
	mut_Lib4-Prim5_F	CT CCG GCG CTG GCG NDT VHG CCG CCG VHG TAC TAT GTG AGC GCG
	mut_Lib4-Prim6_F	CT CCG GCG CTG GCG VHG NDT CCG CCG VHG TAC TAT GTG AGC GCG
	mut_Lib4-Prim7_F	CT CCG GCG CTG GCG VHG VHG CCG CCG NDT TAC TAT GTG AGC GCG
	mut_Lib4-Prim8_F	CT CCG GCG CTG GCG VHG VHG CCG CCG VHG TAC TAT GTG AGC GCG
	mut_Lib4-Prim9_F	CT CCG GCG CTG GCG NDT NDT CCG CCG TGG TAC TAT GTG AGC GCG
	mut_Lib4-Prim10_F	CT CCG GCG CTG GCG NDT TGG CCG CCG NDT TAC TAT GTG AGC GCG
	mut_Lib4-Prim11_F	CT CCG GCG CTG GCG TGG NDT CCG CCG NDT TAC TAT GTG AGC GCG
	mut_Lib4-Prim12_F	CT CCG GCG CTG GCG NDT VHG CCG CCG TGG TAC TAT GTG AGC GCG
	mut_Lib4-Prim13_F	CT CCG GCG CTG GCG NDT TGG CCG CCG VHG TAC TAT GTG AGC GCG
	mut_Lib4-Prim14_F	CT CCG GCG CTG GCG VHG NDT CCG CCG TGG TAC TAT GTG AGC GCG
	mut_Lib4-Prim15_F	CT CCG GCG CTG GCG VHG TGG CCG CCG NDT TAC TAT GTG AGC GCG
	mut_Lib4-Prim16_F	CT CCG GCG CTG GCG TGG NDT CCG CCG VHG TAC TAT GTG AGC GCG
	mut_Lib4-Prim17_F	CT CCG GCG CTG GCG TGG VHG CCG CCG NDT TAC TAT GTG AGC GCG
	mut_Lib4-Prim18_F	CT CCG GCG CTG GCG VHG VHG CCG CCG TGG TAC TAT GTG AGC GCG
	mut_Lib4-Prim19_F	CT CCG GCG CTG GCG VHG TGG CCG CCG VHG TAC TAT GTG AGC GCG
	mut_Lib4-Prim20_F	CT CCG GCG CTG GCG TGG VHG CCG CCG VHG TAC TAT GTG AGC GCG
	mut_Lib4-Prim21_F	CT CCG GCG CTG GCG NDT TGG CCG CCG TGG TAC TAT GTG AGC GCG
	mut_Lib4-Prim22_F	CT CCG GCG CTG GCG TGG NDT CCG CCG TGG TAC TAT GTG AGC GCG
	mut_Lib4-Prim23_F	CT CCG GCG CTG GCG TGG TGG CCG CCG NDT TAC TAT GTG AGC GCG
	mut_Lib4-Prim24_F	CT CCG GCG CTG GCG VHG TGG CCG CCG TGG TAC TAT GTG AGC GCG
	mut_Lib4-Prim25_F	CT CCG GCG CTG GCG TGG VHG CCG CCG TGG TAC TAT GTG AGC GCG
	mut_Lib4-Prim26_F	CT CCG GCG CTG GCG TGG TGG CCG CCG VHG TAC TAT GTG AGC GCG

Supplementary Table 21 (continued). Oligonucleotides used in this study.

Application	Name	Sequence (5'→3')
L4	mut_Lib4-Prim27_F	CT CCG GCG CTG GCG TGG TGG CCG CCG TGG TAC TAT GTG AGC GCG
L5	Lib5_fw	CCG CAG TAC TAT GTG AGC GCG GTT GAC ATC GGT C
	mut_Lib5-Prim1_R	AC ATA GTA CTG CGG CGG AHN AHN CGC CAG AHN CGG AGA CAC CTC GC
	mut_Lib5-Prim2_R	AC ATA GTA CTG CGG CGG AHN AHN CGC CAG CDB CGG AGA CAC CTC GC
	mut_Lib5-Prim3_R	AC ATA GTA CTG CGG CGG AHN CDB CGC CAG AHN CGG AGA CAC CTC GC
	mut_Lib5-Prim4_R	AC ATA GTA CTG CGG CGG CDB AHN CGC CAG AHN CGG AGA CAC CTC GC
	mut_Lib5-Prim5_R	AC ATA GTA CTG CGG CGG AHN CDB CGC CAG CDB CGG AGA CAC CTC GC
	mut_Lib5-Prim6_R	AC ATA GTA CTG CGG CGG CDB AHN CGC CAG CDB CGG AGA CAC CTC GC
	mut_Lib5-Prim7_R	AC ATA GTA CTG CGG CGG CDB CDB CGC CAG AHN CGG AGA CAC CTC GC
	mut_Lib5-Prim8_R	AC ATA GTA CTG CGG CGG CDB CDB CGC CAG CDB CGG AGA CAC CTC GC
	mut_Lib5-Prim9_R	AC ATA GTA CTG CGG CGG AHN AHN CGC CAG CCA CGG AGA CAC CTC GC
	mut_Lib5-Prim10_R	AC ATA GTA CTG CGG CGG AHN CCA CGC CAG AHN CGG AGA CAC CTC GC
	mut_Lib5-Prim11_R	AC ATA GTA CTG CGG CGG CCA AHN CGC CAG AHN CGG AGA CAC CTC GC
	mut_Lib5-Prim12_R	AC ATA GTA CTG CGG CGG AHN CDB CGC CAG CCA CGG AGA CAC CTC GC
	mut_Lib5-Prim13_R	AC ATA GTA CTG CGG CGG AHN CCA CGC CAG CDB CGG AGA CAC CTC GC
	mut_Lib5-Prim14_R	AC ATA GTA CTG CGG CGG CDB AHN CGC CAG CCA CGG AGA CAC CTC GC
	mut_Lib5-Prim15_R	AC ATA GTA CTG CGG CGG CDB CCA CGC CAG AHN CGG AGA CAC CTC GC
	mut_Lib5-Prim16_R	AC ATA GTA CTG CGG CGG CCA AHN CGC CAG CDB CGG AGA CAC CTC GC
	mut_Lib5-Prim17_R	AC ATA GTA CTG CGG CGG CCA CDB CGC CAG AHN CGG AGA CAC CTC GC
	mut_Lib5-Prim18_R	AC ATA GTA CTG CGG CGG CDB CDB CGC CAG CCA CGG AGA CAC CTC GC
	mut_Lib5-Prim19_R	AC ATA GTA CTG CGG CGG CDB CCA CGC CAG CDB CGG AGA CAC CTC GC
	mut_Lib5-Prim20_R	AC ATA GTA CTG CGG CGG CCA CDB CGC CAG CDB CGG AGA CAC CTC GC
	mut_Lib5-Prim21_R	AC ATA GTA CTG CGG CGG AHN CCA CGC CAG CCA CGG AGA CAC CTC GC
	mut_Lib5-Prim22_R	AC ATA GTA CTG CGG CGG CCA AHN CGC CAG CCA CGG AGA CAC CTC GC
	mut_Lib5-Prim23_R	AC ATA GTA CTG CGG CGG CCA CCA CGC CAG AHN CGG AGA CAC CTC GC
	mut_Lib5-Prim24_R	AC ATA GTA CTG CGG CGG CDB CCA CGC CAG CCA CGG AGA CAC CTC GC
	mut_Lib5-Prim25_R	AC ATA GTA CTG CGG CGG CCA CDB CGC CAG CCA CGG AGA CAC CTC GC
	mut_Lib5-Prim26_R	AC ATA GTA CTG CGG CGG CCA CCA CGC CAG CDB CGG AGA CAC CTC GC
	mut_Lib5-Prim27_R	AC ATA GTA CTG CGG CGG CCA CCA CGC CAG CCA CGG AGA CAC CTC GC
L6	Lib6_fw	ATG CCG CCG CAG TAC TAT GTG AGC GCG G
	mut_Lib6_AL_P1_R	C ATA GTA CTG CGG CGG CAT AHN CGC CAG AHN CGG AGA CAC CTC GCC
	mut_Lib6_AL_P2_R	C ATA GTA CTG CGG CGG CAT CDB CGC CAG CDB CGG AGA CAC CTC GCC
	mut_Lib6_AL_P3_R	C ATA GTA CTG CGG CGG CAT AHN CGC CAG CDB CGG AGA CAC CTC GCC
	mut_Lib6_AL_P4_R	C ATA GTA CTG CGG CGG CAT CDB CGC CAG AHN CGG AGA CAC CTC GCC
	mut_Lib6_AL_P5_R	C ATA GTA CTG CGG CGG CAT AHN CGC CAG CCA CGG AGA CAC CTC GCC
	mut_Lib6_AL_P6_R	C ATA GTA CTG CGG CGG CAT CCA CGC CAG AHN CGG AGA CAC CTC GCC
	mut_Lib6_AL_P7_R	C ATA GTA CTG CGG CGG CAT CDB CGC CAG CCA CGG AGA CAC CTC GCC
	mut_Lib6_AL_P8_R	C ATA GTA CTG CGG CGG CAT CCA CGC CAG CDB CGG AGA CAC CTC GCC
	mut_Lib6_AL_P9_R	C ATA GTA CTG CGG CGG CAT CCA CGC CAG CCA CGG AGA CAC CTC GCC
L8 (§primers used also for variants in Table S11)	InvPCR_NDT_fw	GTA AGG ATC CGA ATT CGA GCT CCG
	InvPCR_NDT_rev	GTT CGC ACC GGT AAC CAG CAC CAG
	VD_fw§	TGA TCC CGA AGC CGA ACG TTG AGG GTA TTT ACC TGG ATG AGA AGA GCT GGA AC
	VN_fw	TGA TCC CGA AGC CGA ACG TTG AGG GTA TTT ACC TGA ACG AGA AGA GCT GGA AC
	RD_fw§	TGA TCC CGA AGC CGA ACC GTG AGG GTA TTT ACC TGG ATG AGA AGA GCT GGA AC
	RN_fw	TGA TCC CGA AGC CGA ACC GTG AGG GTA TTT ACC TGA ACG AGA AGA GCT GGA AC
	LG_rev	TTC CTC GAT GCT ACG CCA ACC CGG ACG ACC CAG GCT TTT CAG AAT TTC CAG GCT CGG CGC GGT ATC
	MG_rev§	TTC CTC GAT GCT ACG CCA ACC CGG ACG ACC CAG GCT TTT CAG AAT TTC CAT GCT CGG CGC GGT ATC
	LI_rev	TTC CTC GAT GCT ACG CCA GAT CGG ACG ACC CAG GCT TTT CAG AAT TTC CAG GCT CGG CGC GGT ATC
	MI_rev§	TTC CTC GAT GCT ACG CCA GAT CGG ACG ACC CAG GCT TTT CAG AAT TTC CAT GCT CGG CGC GGT ATC
	T342M_fw	AGA CCT GGT TGG CAG CGA GAT GGC GTA AGG ATC
	T342M_rev	GAT CCT TAC GCC ATC TCG CTG CCA ACC AGG TCT
L9	L241_NDT_XWT_fw	G TCT CCG GCG CTG GCG NDT TGG CCG CCG ACC TAC TAT GTG AGC GCG G
	L241_VHG_XWT_fw	G TCT CCG GCG CTG GCG VHG TGG CCG CCG ACC TAC TAT GTG AGC GCG G
	L241_TGG_XWT_fw	G TCT CCG GCG CTG GCG TGG TGG CCG CCG ACC TAC TAT GTG AGC GCG G

Supplementary Table 21 (continued). Oligonucleotides used in this study.

Application	Name	Sequence (5'→3')
L9	L241_NDT_XWT_rev	C CGC GCT CAC ATA GTA GGT CGG CGG CCA AHN CGC CAG CGC CGG AGA C
	L241_VHG_XWT_rev	C CGC GCT CAC ATA GTA GGT CGG CGG CCA CDB CGC CAG CGC CGG AGA C
	L241_TGG_XWT_rev	C CGC GCT CAC ATA GTA GGT CGG CGG CCA CCA CGC CAG CGC CGG AGA C
	Q245N_CWN_fw	G GCG TGT TGG CCG CCG AAT TAC TAT GTG AGC GCG G
	Q245N_CWN_rev	C CGC GCT CAC ATA GTA ATT CGG CGG CCA ACA CGC C
	L241_RYT_XWS_fw	G TCT CCG GCG CTG GCG RYT TGG CCG CCG AGT TAC T
	L241_RYT_XWS_rev	A GTA ACT CGG CGG CCA ARY CGC CAG CGC CGG AGA C
	L241Q_QWS_fw	G TCT CCG GCG CTG GCG CAG TGG CCG CCG AGT TAC T
	L241Q_QWS_rev	A GTA ACT CGG CGG CCA CTG CGC CAG CGC CGG AGA C
	L241S_SWS_fw	G TCT CCG GCG CTG GCG AGT TGG CCG CCG AGT TAC T
	L241S_SWS_rev	A GTA ACT CGG CGG CCA ACT CGC CAG CGC CGG AGA C
	L241_AST_XFT_fw	G TCT CCG GCG CTG GCG AST TTT CCG CCG ACG TAC TAT G
	L241_AST_XFT_rev	C ATA GTA CGT CGG CGG AAA AST CGC CAG CGC CGG AGA C
	Y242F_VFT_fw	G TCT CCG GCG CTG GCG GTG TTT CCG CCG ACG TAC TAT G
	Y242F_VFT_rev	C ATA GTA CGT CGG CGG AAA CAC CGC CAG CGC CGG AGA C
	L241I_IYT_fw	G TCT CCG GCG CTG GCG ATT TAT CCG CCG ACG TAC T
	L241I_IYT_rev	A GTA CGT CGG CGG ATA AAT CGC CAG CGC CGG AGA C
	L241M_MMN_fw	G TCT CCG GCG CTG GCG ATG ATG CCG CCG AAT TAC T
	L241M_MMN_rev	A GTA ATT CGG CGG CAT CAT CGC CAG CGC CGG AGA C
L10	fw_GCG_174	GC GAT CCG CAA AAG AGC GCG TGG GTG TAT GCG GCG AGC
	rv_GCG_174	GCT CGC CGC ATA CAC CCA CGC GCT CTT TTG CGG ATC GC
	fw_VTG_174	GC GAT CCG CAA AAG AGC VTG TGG GTG TAT GCG GCG AGC
	rv_VTG_174	GCT CGC CGC ATA CAC CCA CAB GCT CTT TTG CGG ATC GC
	fw_ATT_174	GC GAT CCG CAA AAG AGC ATT TGG GTG TAT GCG GCG AGC
	rv_ATT_174	GCT CGC CGC ATA CAC CCA AAT GCT CTT TTG CGG ATC GC
	fw_AAS_238_CAG_241	G TTT AAC GGC GAG GTG TCT CCG AAS CTG GCG CAG TGG CCG CCG AGT TAC TAT GTG
	rv_AAS_238_CAG_241	CAC ATA GTA ACT CGG CGG CCA CTG CGC CAG STT CGG AGA CAC CTC GCC GTT AAA C
	fw_AAS_238_ATG_241	G TTT AAC GGC GAG GTG TCT CCG AAS CTG GCG ATG TGG CCG CCG AGT TAC TAT GTG
	rv_AAS_238_ATG_241	CAC ATA GTA ACT CGG CGG CCA CAT CGC CAG STT CGG AGA CAC CTC GCC GTT AAA C
	fw_AAS_238_AGC_241	G TTT AAC GGC GAG GTG TCT CCG AAS CTG GCG AGC TGG CCG CCG AGT TAC TAT GTG
	rv_AAS_238_AGC_241	CAC ATA GTA ACT CGG CGG CCA GCT CGC CAG STT CGG AGA CAC CTC GCC GTT AAA C
	fw_CTG_238_CAG_241	G TTT AAC GGC GAG GTG TCT CCG CTG CTG GCG CAG TGG CCG CCG AGT TAC TAT GTG
	rv_CTG_238_CAG_241	CAC ATA GTA ACT CGG CGG CCA CTG CGC CAG CAG CGG AGA CAC CTC GCC GTT AAA C
	fw_CTG_238_ATG_241	G TTT AAC GGC GAG GTG TCT CCG CTG CTG GCG ATG TGG CCG CCG AGT TAC TAT GTG
	rv_CTG_238_ATG_241	CAC ATA GTA ACT CGG CGG CCA CAT CGC CAG CAG CGG AGA CAC CTC GCC GTT AAA C
	fw_CTG_238_AGC_241	G TTT AAC GGC GAG GTG TCT CCG CTG CTG GCG AGC TGG CCG CCG AGT TAC TAT GTG
	rv_CTG_238_AGC_241	CAC ATA GTA ACT CGG CGG CCA GCT CGC CAG CAG CGG AGA CAC CTC GCC GTT AAA C
	fw_SGC_238_CAG_241	G TTT AAC GGC GAG GTG TCT CCG SGC CTG GCG CAG TGG CCG CCG AGT TAC TAT GTG
	rv_SGC_238_CAG_241	CAC ATA GTA ACT CGG CGG CCA CTG CGC CAG GCS CGG AGA CAC CTC GCC GTT AAA C
	fw_SGC_238_ATG_241	G TTT AAC GGC GAG GTG TCT CCG SGC CTG GCG ATG TGG CCG CCG AGT TAC TAT GTG
	rv_SGC_238_ATG_241	CAC ATA GTA ACT CGG CGG CCA CAT CGC CAG GCS CGG AGA CAC CTC GCC GTT AAA C
	fw_SGC_238_AGC_241	G TTT AAC GGC GAG GTG TCT CCG SGC CTG GCG AGC TGG CCG CCG AGT TAC TAT GTG
	rv_SGC_238_AGC_241	CAC ATA GTA ACT CGG CGG CCA GCT CGC CAG GCS CGG AGA CAC CTC GCC GTT AAA C
L11	mut_243-246-P1_fw	G AAG CTG GCG ATG TGG NDT CCG AGT NDT TAT GTG AGC GCG GTT G
	mut_243-246-P2_fw	G AAG CTG GCG ATG TGG VHG CCG AGT VHG TAT GTG AGC GCG GTT G
	mut_243-246-P3_fw	G AAG CTG GCG ATG TGG NDT CCG AGT VHG TAT GTG AGC GCG GTT G
	mut_243-246-P4_fw	G AAG CTG GCG ATG TGG VHG CCG AGT NDT TAT GTG AGC GCG GTT G
	mut_243-246-P5_fw	G AAG CTG GCG ATG TGG NDT CCG AGT TGG TAT GTG AGC GCG GTT G
	mut_243-246-P6_fw	G AAG CTG GCG ATG TGG TGG CCG AGT NDT TAT GTG AGC GCG GTT G
	mut_243-246-P7_fw	G AAG CTG GCG ATG TGG VHG CCG AGT TGG TAT GTG AGC GCG GTT G
	mut_243-246-P8_fw	G AAG CTG GCG ATG TGG TGG CCG AGT VHG TAT GTG AGC GCG GTT G
	mut_243-246-P9_fw	G AAG CTG GCG ATG TGG TGG CCG AGT TGG TAT GTG AGC GCG GTT G
	mut_243-246-P1_rv	C AAC CGC GCT CAC ATA AHN ACT CGG AHN CCA CAT CGC CAG CTT C
	mut_243-246-P2_rv	C AAC CGC GCT CAC ATA CDB ACT CGG CDB CCA CAT CGC CAG CTT C

Supplementary Table 21 (continued). Oligonucleotides used in this study.

Application	Name	Sequence (5'→3')
L11	mut_243-246-P3_fw	G AAG CTG GCG ATG TGG NDT CCG AGT VHG TAT GTG AGC GCG GTT G
	mut_243-246-P4_fw	G AAG CTG GCG ATG TGG VHG CCG AGT NDT TAT GTG AGC GCG GTT G
	mut_243-246-P5_fw	G AAG CTG GCG ATG TGG NDT CCG AGT TGG TAT GTG AGC GCG GTT G
	mut_243-246-P6_fw	G AAG CTG GCG ATG TGG TGG CCG AGT NDT TAT GTG AGC GCG GTT G
	mut_243-246-P7_fw	G AAG CTG GCG ATG TGG VHG CCG AGT TGG TAT GTG AGC GCG GTT G
	mut_243-246-P8_fw	G AAG CTG GCG ATG TGG TGG CCG AGT VHG TAT GTG AGC GCG GTT G
	mut_243-246-P9_fw	G AAG CTG GCG ATG TGG TGG CCG AGT TGG TAT GTG AGC GCG GTT G
	mut_243-246-P1_rv	C AAC CGC GCT CAC ATA AHN ACT CGG AHN CCA CAT CGC CAG CTT C
	mut_243-246-P2_rv	C AAC CGC GCT CAC ATA CDB ACT CGG CDB CCA CAT CGC CAG CTT C
	mut_243-246-P3_rv	C AAC CGC GCT CAC ATA AHN ACT CGG CDB CCA CAT CGC CAG CTT C
	mut_243-246-P4_rv	C AAC CGC GCT CAC ATA CDB ACT CGG AHN CCA CAT CGC CAG CTT C
	mut_243-246-P5_rv	C AAC CGC GCT CAC ATA AHN ACT CGG CCA CCA CAT CGC CAG CTT C
	mut_243-246-P6_rv	C AAC CGC GCT CAC ATA CCA ACT CGG AHN CCA CAT CGC CAG CTT C
	mut_243-246-P7_rv	C AAC CGC GCT CAC ATA CDB ACT CGG CCA CCA CAT CGC CAG CTT C
	mut_243-246-P8_rv	C AAC CGC GCT CAC ATA CCA ACT CGG CDB CCA CAT CGC CAG CTT C
	mut_243-246-P9_rv	C AAC CGC GCT CAC ATA CCA ACT CGG CCA CCA CAT CGC CAG CTT C
L12	P206X_fw	CTG AAC GCG GTT CTG NNK AAC TAC ACC ATC GGT AC
	P206X_rv	GT ACC GAT GGT GTA GTT MNN CAG AAC CGC GTT CAG
	N207X_fw	G AAC GCG GTT CTG CCG NNK TAC ACC ATC GGT ACC
	N207X_rv	GGT ACC GAT GGT GTA MNN CGG CAG AAC CGC GTT C
	Y208X_fw	GCG GTT CTG CCG AAC NNK ACC ATC GGT ACC ATT TTC
	Y208X_rv	GAA AAT GGT ACC GAT GGT MNN GTT CGG CAG AAC CGC
	T209X_fw	GTT CTG CCG AAC TAC NNK ATC GGT ACC ATT TTC G
	T209X_rv	C GAA AAT GGT ACC GAT MNN GTA GTT CGG CAG AAC
	S222X_fw	GAA ACC CAG AGC GGT NNK ACC AGC GGC TGG ATG
	S222X_rv	CAT CCA GCC GCT GGT MNN ACC GCT CTG GGT TTC
	T223X_M228_fw	CC CAG AGC GGT AGC NNK AGC GGC TGG ATG ATG AG
	T223X_M228_rv	CT CAT CAT CCA GCC GCT MNN GCT ACC GCT CTG GG
	S224X_M228_fw	CAG AGC GGT AGC ACC NNK GGC TGG ATG ATG AGC
	S224X_M228_rv	GCT CAT CAT CCA GCC MNN GGT GCT ACC GCT CTG
	W226X_M228_fw	GGT AGC ACC AGC GGC NNK ATG ATG AGC CTG TTT AAC
	W226X_M228_rv	GTT AAA CAG GCT CAT CAT MNN GCC GCT GGT GCT ACC
L13	fw_NDT_234_NDT_235	GTT CTG ACC AGC AGC NDT NDT AGC GCG CTG ATC CCG
	rev_AHN_234_AHN_235	CGG GAT CAG CGC GCT AHN AHN GCT GCT GGT CAG AAC
	fw_VHG_234_VHG_235	GTT CTG ACC AGC AGC VHG VHG AGC GCG CTG ATC CCG
	rev_CDB_234_CDB_235	CGG GAT CAG CGC GCT CDB CDB GCT GCT GGT CAG AAC
	fw_NDT_234_VHG_235	GTT CTG ACC AGC AGC NDT VHG AGC GCG CTG ATC CCG
	rev_AHN_234_CDB_235	CGG GAT CAG CGC GCT AHN CDB GCT GCT GGT CAG AAC
	fw_VHG_234_NDT_235	GTT CTG ACC AGC AGC VHG NDT AGC GCG CTG ATC CCG
	rev_CDB_234_AHN_235	CGG GAT CAG CGC GCT CDB AHN GCT GCT GGT CAG AAC
	fw_NDT_234_TGG_235	GTT CTG ACC AGC AGC NDT TGG AGC GCG CTG ATC CCG
	rev_AHN_234_CCA_235	CGG GAT CAG CGC GCT AHN CCA GCT GCT GGT CAG AAC
	fw_TGG_234_NDT_235	GTT CTG ACC AGC AGC TGG NDT AGC GCG CTG ATC CCG
	rev_CCA_234_AHN_235	CGG GAT CAG CGC GCT CCA AHN GCT GCT GGT CAG AAC
	fw_VHG_234_TGG_235	GTT CTG ACC AGC AGC VHG TGG AGC GCG CTG ATC CCG
	rev_CDB_234_CCA_235	CGG GAT CAG CGC GCT CDB CCA GCT GCT GGT CAG AAC
	fw_TGG_234_VHG_235	GTT CTG ACC AGC AGC TGG VHG AGC GCG CTG ATC CCG
	rev_CCA_234_CDB_235	CGG GAT CAG CGC GCT CCA CDB GCT GCT GGT CAG AAC
	fw_TGG_234_TGG_235	GTT CTG ACC AGC AGC TGG TGG AGC GCG CTG ATC CCG
	rev_CCA_234_CCA_235	CGG GAT CAG CGC GCT CCA CCA GCT GCT GGT CAG AAC

4. *SsaI*-KRED sequences

Nucleotide sequences of the wild type and hit variants

>*SsaI*-KRED_WT_nucleotide_sequence
ATGGCGAAGATCGACAACGCGGTGCTGCCGGAAGGTAGCCTGGTGCTGGTTACCGGTGCGAACGGTTTTGTGGCGAGCCACGTGGTTGAGCAGCTGCT
GGAACACGGTTACAAGGTTCGTGGTACCGCGCGTAGCGCGAGCAAACTGGCGAACCTGCAAAAGCGTTGGGACGCGAAATACCCGGGTCGTTTTGAGA
CCGCGGTGGTTGAAGACATGCTGAAGCAGGGCGCGTATGATGAAGTGATCAAGGGTGCGGCGGGCGTTGCGCACATTGCGAGCGTGGTTAGCTTCAGC
AACAAGTATGATGAGGTGGTTACCCCGGCGATCGGTGGCACCCTGAACGCGCTGCGTGCGGCGGCGGCGACCCCGAGCGTGAAACGTTTTGTTCTGAC
CAGCAGCACCGTGAGCGCGCTGATCCCGAAGCCGAACGTTGAGGGTATTTACCTGGATGAGAAGAGCTGGAACCTGGAGAGCATTGACAAGGCGAAAA
CCCTGCCGGAAAGCGATCCGCAAAAGAGCCTGTGGGTGTATGCGGCGAGCAAAACCGAGGCGGAACTGGCGGCGTGGAAGTTCATGGACGAGAACAAA
CCGCACTTTACCCTGAACGCGGTTCTGCCGAACTACACCATCGGTACCATTTTCGATCCGGAAACCCAGAGCGGTAGCACCAGCGGCTGGATGATGAG
CCTGTTTAACGGCGAGGTGTCTCCGGCGCTGGCGCTGATGCCGCCGCAGTACTATGTGAGCGCGGTTGACATCGGTCTGCTGCACCTGGGTTGCCTGG
TGCTGCCGCAAATTGAGCGTCGTCGTGTTTACGGTACCGCGGGCACCTTCGATTGGAACACCGTTCTGGCGACCTTTCGTAAGCTGTATCCGAGCAAA
ACCTTCCCGGCGGACTTTCCGGATCAGGGTCAAGACCTGAGCAAGTTCGATACCGCGCCGAGCCTGGAAATTCTGAAAAGCCTGGGTCGTCCGGGTTG
GCGTAGCATCGAGGAAAGCATTAAAGACCTGGTTGGCAGCGAGACCGCGTAA

>*SsaI*-KRED_M1_nucleotide_sequence
ATGGCGAAGATCGACAACGCGGTGCTGCCGGAAGGTAGCCTGGTGCTGGTTACCGGTGCGAACGGTTTTGTGGCGAGCCACGTGGTTGAGCAGCTGCT
GGAACACGGTTACAAGGTTCGTGGTACCGCGCGTAGCGCGAGCAAACTGGCGAACCTGCAAAAGCGTTGGGACGCGAAATACCCGGGTCGTTTTGAGA
CCGCGGTGGTTGAAGACATGCTGAAGCAGGGCGCGTATGATGAAGTGATCAAGGGTGCGGCGGGCGTTGCGCACATTGCGAGCGTGGTTAGCTGGAGC
AACAAGTATGATGAGGTGGTTACCCCGGCGATCGGTGGCACCCTGAACGCGCTGCGTGCGGCGGCGGCGACCCCGAGCGTGAAACGTTTTGTTCTGAC
CAGCAGCACCGTGAGCGCGCTGATCCCGAAGCCGAACGTTGAGGGTATTTACCTGGATGAGAAGAGCTGGAACCTGGAGAGCATTGACAAGGCGAAAA
CCCTGCCGGAAAGCGATCCGCAAAAGAGCCTGTGGGTGTATGCGGCGAGCAAAACCGAGGCGGAACTGGCGGCGTGGAAGTTCATGGACGAGAACAAA
CCGCACTTTACCCTGAACGCGGTTCTGCCGAACTACACCATCGGTACCATTTTCGATCCGGAAACCCAGAGCGGTAGCACCAGCGGCTGGATGATGAG
CCTGTTTAACGGCGAGGTGTCTCCGGCGCTGGCGATGTGGCCGCCGAGTTACTATGTGAGCGCGGTTGACATCGGTCTGCTGCACCTGGGTTGCCTGG
TGCTGCCGCAAATTGAGCGTCGTCGTGTTTACGGTACCGCGGGCACCTTCGATTGGAACACCGTTCTGGCGACCTTTCGTAAGCTGTATCCGAGCAAA
ACCTTCCCGGCGGACTTTCCGGATCAGGGTCAAGACCTGAGCAAGTTCGATACCGCGCCGAGCCTGGAAATTCTGAAAAGCCTGGGTCGTCCGGGTTG
GCGTAGCATCGAGGAAAGCATTAAAGACCTGGTTGGCAGCGAGACCGCGTAA

>*SsaI*-KRED_M2_nucleotide_sequence
ATGGCGAAGATCGACAACGCGGTGCTGCCGGAAGGTAGCCTGGTGCTGGTTACCGGTGCGAACGGTTTTGTGGCGAGCCACGTGGTTGAGCAGCTGCT
GGAACACGGTTACAAGGTTCGTGGTACCGCGCGTAGCGCGAGCAAACTGGCGAACCTGCAAAAGCGTTGGGACGCGAAATACCCGGGTCGTTTTGAGA
CCGCGGTGGTTGAAGACATGCTGAAGCAGGGCGCGTATGATGAAGTGATCAAGGGTGCGGCGGGCGTTGCGCACATTGCGAGCGTGGTTAGCTGGAGC
AACAAGTATGATGAGGTGGTTACCCCGGCGATCGGTGGCACCCTGAACGCGCTGCGTGCGGCGGCGGCGACCCCGAGCGTGAAACGTTTTGTTCTGAC
CAGCAGCACCGTGAGCGCGCTGATCCCGAAGCCGAACGTTGAGGGTATTTACCTGGATGAGAAGAGCTGGAACCTGGAGAGCATTGACAAGGCGAAAA
CCCTGCCGGAAAGCGATCCGCAAAAGAGCCTGTGGGTGTATGCGGCGAGCAAAACCGAGGCGGAACTGGCGGCGTGGAAGTTCATGGACGAGAACAAA
CCGCACTTTACCCTGAACGCGGTTCTGCCGAACTACACCATCGGTACCATTTTCGATCCGGAAACCCAGAGCGGTAGCACCAGCGGCTGGATGATGAG
CCTGTTTAACGGCGAGGTGTCTCCGGCGCTGGCGATGTGGCCGCCGAGTTACTATGTGAGCGCGGTTGACATCGGTCTGCTGCACCTGGGTTGCCTGG
TGCTGCCGCAAATTGAGCGTCGTCGTGTTTACGGTACCGCGGGCACCTTCGATTGGAACACCGTTCTGGCGACCTTTCGTAAGCTGTATCCGAGCAAA
ACCTTCCCGGCGGACTTTCCGGATCAGGGTCAAGACCTGAGCAAGTTCGATACCGCGCCGAGCATGGAAATTCTGAAAAGCCTGGGTCGTCCGGGTTG
GCGTAGCATCGAGGAAAGCATTAAAGACCTGGTTGGCAGCGAGATGGCGTAA

>*SsaI*-KRED_M3_nucleotide_sequence
ATGGCGAAGATCGACAACGCGGTGCTGCCGGAAGGTAGCCTGGTGCTGGTTACCGGTGCGAACGGTTTTGTGGCGAGCCACGTGGTTGAGCAGCTGCT
GGAACACGGTTACAAGGTTCGTGGTACCGCGCGTAGCGCGAGCAAACTGGCGAACCTGCAAAAGCGTTGGGACGCGAAATACCCGGGTCGTTTTGAGA
CCGCGGTGGTTGAAGACATGCTGAAGCAGGGCGCGTATGATGAAGTGATCAAGGGTGCGGCGGGCGTTGCGCACATTGCGAGCGTGGTTAGCTGGAGC
AACAAGTATGATGAGGTGGTTACCCCGGCGATCGGTGGCACCCTGAACGCGCTGCGTGCGGCGGCGGCGACCCCGAGCGTGAAACGTTTTGTTCTGAC
CAGCAGCACCGTGAGCGCGCTGATCCCGAAGCCGAACGTTGAGGGTATTTACCTGGATGAGAAGAGCTGGAACCTGGAGAGCATTGACAAGGCGAAAA
CCCTGCCGGAAAGCGATCCGCAAAAGAGCCTGTGGGTGTATGCGGCGAGCAAAACCGAGGCGGAACTGGCGGCGTGGAAGTTCATGGACGAGAACAAA
CCGCACTTTACCCTGAACGCGGTTCTGCCGAACTACACCATCGGTACCATTTTCGATCCGGAAACCCAGAGCGGTAGCACCAGCGGCTGGATGATGAG
CCTGTTTAACGGCGAGGTGTCTCCGAAGCTGGCGATGTGGCCGCCGAGTTACTATGTGAGCGCGGTTGACATCGGTCTGCTGCACCTGGGTTGCCTGG
TGCTGCCGCAAATTGAGCGTCGTCGTGTTTACGGTACCGCGGGCACCTTCGATTGGAACACCGTTCTGGCGACCTTTCGTAAGCTGTATCCGAGCAAA
ACCTTCCCGGCGGACTTTCCGGATCAGGGTCAAGACCTGAGCAAGTTCGATACCGCGCCGAGCATGGAAATTCTGAAAAGCCTGGGTCGTCCGGGTTG
GCGTAGCATCGAGGAAAGCATTAAAGACCTGGTTGGCAGCGAGATGGCGTAA

>*Ssal*-KRED_M4_nucleotide_sequence

ATGGCGAAGATCGACAACGCGGTGCTGCCGGAAGGTAGCCTGGTGCTGGTTACCGGTGCGAACGGTTTTGTGGCGAGCCACGTGGTTGAGCAGCTGCT
GGAACACGGTTACAAGGTTCGTGGTACCGCGCGTAGCGCGAGCAAACTGGCGAACCTGCAAAAGCGTTGGGACGCGAAATACCCGGGTCGTTTTGAGA
CCGCGGTGGTTGAAGACATGCTGAAGCAGGGCGCGTATGATGAAGTGATCAAGGGTGCGGCGGGCGTTGCGCACATTGCGAGCGTGGTTAGCTGGAGC
AACAAGTATGATGAGGTGGTTACCCCGGCGATCGGTGGCACCCTGAACGCGCTGCGTGCGGCGGCGGCGACCCCGAGCGTGAAACGTTTTGTTCTGAC
CAGCAGCACCGTGAGCGCGCTGATCCCGAAGCCGAACGTTGAGGGTATTTACCTGGATGAGAAGAGCTGGAACCTGGAGAGCATTGACAAGGCGAAAA
CCCTGCCGGAAAGCGATCCGCAAAAGAGCCTGTGGGTGTATGCGGCGAGCAAAACCGAGGCGGAACTGGCGGCGTGGAAGTTCATGGACGAGAACAAA
CCGCACTTTACCCTGAACGCGGTTCTGCCGAACTACACCATCGGTACCATTTTCGATCCGGAAACCCAGAGCGGTAGCACCAGCGGCTGGATGATGAG
CCTGTTTAACGGCGAGGTGTCTCCGAAGCTGGCGATGTGGCCGCCGAGTGGTTATGTGAGCGCGGTTGACATCGGTCTGCTGCACCTGGGTTGCCTGG
TGCTGCCGCAAATTGAGCGTCGTCGTGTTTACGGTACCGCGGGCACCTTCGATTGGAACACCGTTCTGGCGACCTTTCGTAAGCTGTATCCGAGCAAA
ACCTTCCCGGCGGACTTTCCGGATCAGGGTCAAGACCTGAGCAAGTTCGATACCGCGCCGAGCATGGAAATTCTGAAAAGCCTGGGTCGTCCGGGTTG
GCGTAGCATCGAGGAAAGCATTAAAGACCTGGTTGGCAGCGAGATGGCGTAA

>*Ssal*-KRED_M5_nucleotide_sequence

ATGGCGAAGATCGACAACGCGGTGCTGCCGGAAGGTAGCCTGGTGCTGGTTACCGGTGCGAACGGTTTTGTGGCGAGCCACGTGGTTGAGCAGCTGCT
GGAACACGGTTACAAGGTTCGTGGTACCGCGCGTAGCGCGAGCAAACTGGCGAACCTGCAAAAGCGTTGGGACGCGAAATACCCGGGTCGTTTTGAGA
CCGCGGTGGTTGAAGACATGCTGAAGCAGGGCGCGTATGATGAAGTGATCAAGGGTGCGGCGGGCGTTGCGCACATTGCGAGCGTGGTTAGCTGGAGC
AACAAGTATGATGAGGTGGTTACCCCGGCGATCGGTGGCACCCTGAACGCGCTGCGTGCGGCGGCGGCGACCCCGAGCGTGAAACGTTTTGTTCTGAC
CAGCAGCACCGTGAGCGCGCTGATCCCGAAGCCGAACGTTGAGGGTATTTACCTGGATGAGAAGAGCTGGAACCTGGAGAGCATTGACAAGGCGAAAA
CCCTGCCGGAAAGCGATCCGCAAAAGAGCCTGTGGGTGTATGCGGCGAGCAAAACCGAGGCGGAACTGGCGGCGTGGAAGTTCATGGACGAGAACAAA
CCGCACTTTACCCTGAACGCGGTTCTGCCGAACTACACCATCGGTACCATTTTCGATCCGGAAACCCAGAGCGGTAGCACCGCTGGCTGGATGATGAG
CCTGTTTAACGGCGAGGTGTCTCCGAAGCTGGCGATGTGGCCGCCGAGTGGTTATGTGAGCGCGGTTGACATCGGTCTGCTGCACCTGGGTTGCCTGG
TGCTGCCGCAAATTGAGCGTCGTCGTGTTTACGGTACCGCGGGCACCTTCGATTGGAACACCGTTCTGGCGACCTTTCGTAAGCTGTATCCGAGCAAA
ACCTTCCCGGCGGACTTTCCGGATCAGGGTCAAGACCTGAGCAAGTTCGATACCGCGCCGAGCATGGAAATTCTGAAAAGCCTGGGTCGTCCGGGTTG
GCGTAGCATCGAGGAAAGCATTAAAGACCTGGTTGGCAGCGAGATGGCGTAA

>*Ssal*-KRED_M6_nucleotide_sequence

ATGGCGAAGATCGACAACGCGGTGCTGCCGGAAGGTAGCCTGGTGCTGGTTACCGGTGCGAACGGTTTTGTGGCGAGCCACGTGGTTGAGCAGCTGCT
GGAACACGGTTACAAGGTTCGTGGTACCGCGCGTAGCGCGAGCAAACTGGCGAACCTGCAAAAGCGTTGGGACGCGAAATACCCGGGTCGTTTTGAGA
CCGCGGTGGTTGAAGACATGCTGAAGCAGGGCGCGTATGATGAAGTGATCAAGGGTGCGGCGGGCGTTGCGCACATTGCGAGCGTGGTTAGCTGGAGC
AACAAGTATGATGAGGTGGTTACCCCGGCGATCGGTGGCACCCTGAACGCGCTGCGTGCGGCGGCGGCGACCCCGAGCGTGAAACGTTTTGTTCTGAC
CAGCAGCGTTGTTAGCGCGCTGATCCCGAAGCCGAACGTTGAGGGTATTTACCTGGATGAGAAGAGCTGGAACCTGGAGAGCATTGACAAGGCGAAAA
CCCTGCCGGAAAGCGATCCGCAAAAGAGCCTGTGGGTGTATGCGGCGAGCAAAACCGAGGCGGAACTGGCGGCGTGGAAGTTCATGGACGAGAACAAA
CCGCACTTTACCCTGAACGCGGTTCTGCCGAACTACACCATCGGTACCATTTTCGATCCGGAAACCCAGAGCGGTAGCACCGCTGGCTGGATGATGAG
CCTGTTTAACGGCGAGGTGTCTCCGAAGCTGGCGATGTGGCCGCCGAGTGGTTATGTGAGCGCGGTTGACATCGGTCTGCTGCACCTGGGTTGCCTGG
TGCTGCCGCAAATTGAGCGTCGTCGTGTTTACGGTACCGCGGGCACCTTCGATTGGAACACCGTTCTGGCGACCTTTCGTAAGCTGTATCCGAGCAAA
ACCTTCCCGGCGGACTTTCCGGATCAGGGTCAAGACCTGAGCAAGTTCGATACCGCGCCGAGCATGGAAATTCTGAAAAGCCTGGGTCGTCCGGGTTG
GCGTAGCATCGAGGAAAGCATTAAAGACCTGGTTGGCAGCGAGATGGCGTAA

Amino acid sequences of the wild type and hit variants

>*Ssal*-KRED_WT_amino_acid_sequence
MAKIDNAVLPEGSLVLVTGANGFVASHVVEQLLEHGYKVRGTARSASKLANLQKRWDAKYPGRFETAVVEDMLKQGAYDEVIKGAAGVAHIASVVSFS
NKYDEVVTPAIGGTLNALRAAAATPSVKRFVLTSSTVSALIPKPNVEGIYLDEKSWNLESIDKAKTLPESDPQKSLWVYAASKTEAELAAWKFMDENK
PHFTLNAVLPNYTIGTIFDPETQSGSTSGWMMSLFNGEVSPALALMPPQYYVSAVDIGLLHLGCLVLPQIERRRVYGTAGTFDWNTVLATFRKLYPSK
TFPADFPDQGQDLSKFDTAPSLEILKSLGRPGWRSIEESIKDLVGSETA

>*Ssal*-KRED_M1_amino_acid_sequence
MAKIDNAVLPEGSLVLVTGANGFVASHVVEQLLEHGYKVRGTARSASKLANLQKRWDAKYPGRFETAVVEDMLKQGAYDEVIKGAAGVAHIASVVSWS
NKYDEVVTPAIGGTLNALRAAAATPSVKRFVLTSSTVSALIPKPNVEGIYLDEKSWNLESIDKAKTLPESDPQKSLWVYAASKTEAELAAWKFMDENK
PHFTLNAVLPNYTIGTIFDPETQSGSTSGWMMSLFNGEVSPALAMWPPSYYVSAVDIGLLHLGCLVLPQIERRRVYGTAGTFDWNTVLATFRKLYPSK
TFPADFPDQGQDLSKFDTAPSLEILKSLGRPGWRSIEESIKDLVGSETA

>*Ssal*-KRED_M2_amino_acid_sequence
MAKIDNAVLPEGSLVLVTGANGFVASHVVEQLLEHGYKVRGTARSASKLANLQKRWDAKYPGRFETAVVEDMLKQGAYDEVIKGAAGVAHIASVVSWS
NKYDEVVTPAIGGTLNALRAAAATPSVKRFVLTSSTVSALIPKPNVEGIYLDEKSWNLESIDKAKTLPESDPQKSLWVYAASKTEAELAAWKFMDENK
PHFTLNAVLPNYTIGTIFDPETQSGSTSGWMMSLFNGEVSPALAMWPPSYYVSAVDIGLLHLGCLVLPQIERRRVYGTAGTFDWNTVLATFRKLYPSK
TFPADFPDQGQDLSKFDTAPSMEILKSLGRPGWRSIEESIKDLVGSEMA

>*Ssal*-KRED_M3_amino_acid_sequence
MAKIDNAVLPEGSLVLVTGANGFVASHVVEQLLEHGYKVRGTARSASKLANLQKRWDAKYPGRFETAVVEDMLKQGAYDEVIKGAAGVAHIASVVSWS
NKYDEVVTPAIGGTLNALRAAAATPSVKRFVLTSSTVSALIPKPNVEGIYLDEKSWNLESIDKAKTLPESDPQKSLWVYAASKTEAELAAWKFMDENK
PHFTLNAVLPNYTIGTIFDPETQSGSTSGWMMSLFNGEVSPKLAMWPPSYYVSAVDIGLLHLGCLVLPQIERRRVYGTAGTFDWNTVLATFRKLYPSK
TFPADFPDQGQDLSKFDTAPSMEILKSLGRPGWRSIEESIKDLVGSEMA

>*Ssal*-KRED_M4_amino_acid_sequence
MAKIDNAVLPEGSLVLVTGANGFVASHVVEQLLEHGYKVRGTARSASKLANLQKRWDAKYPGRFETAVVEDMLKQGAYDEVIKGAAGVAHIASVVSWS
NKYDEVVTPAIGGTLNALRAAAATPSVKRFVLTSSTVSALIPKPNVEGIYLDEKSWNLESIDKAKTLPESDPQKSLWVYAASKTEAELAAWKFMDENK
PHFTLNAVLPNYTIGTIFDPETQSGSTSGWMMSLFNGEVSPKLAMWPPSGYVSAVDIGLLHLGCLVLPQIERRRVYGTAGTFDWNTVLATFRKLYPSK
TFPADFPDQGQDLSKFDTAPSMEILKSLGRPGWRSIEESIKDLVGSEMA

>*Ssal*-KRED_M5_amino_acid_sequence
MAKIDNAVLPEGSLVLVTGANGFVASHVVEQLLEHGYKVRGTARSASKLANLQKRWDAKYPGRFETAVVEDMLKQGAYDEVIKGAAGVAHIASVVSWS
NKYDEVVTPAIGGTLNALRAAAATPSVKRFVLTSSTVSALIPKPNVEGIYLDEKSWNLESIDKAKTLPESDPQKSLWVYAASKTEAELAAWKFMDENK
PHFTLNAVLPNYTIGTIFDPETQSGSTAGWMMSLFNGEVSPKLAMWPPSGYVSAVDIGLLHLGCLVLPQIERRRVYGTAGTFDWNTVLATFRKLYPSK
TFPADFPDQGQDLSKFDTAPSMEILKSLGRPGWRSIEESIKDLVGSEMA

>*Ssal*-KRED_M6_amino_acid_sequence
MAKIDNAVLPEGSLVLVTGANGFVASHVVEQLLEHGYKVRGTARSASKLANLQKRWDAKYPGRFETAVVEDMLKQGAYDEVIKGAAGVAHIASVVSWS
NKYDEVVTPAIGGTLNALRAAAATPSVKRFVLTSSVVSALIPKPNVEGIYLDEKSWNLESIDKAKTLPESDPQKSLWVYAASKTEAELAAWKFMDENK
PHFTLNAVLPNYTIGTIFDPETQSGSTAGWMMSLFNGEVSPKLAMWPPSGYVSAVDIGLLHLGCLVLPQIERRRVYGTAGTFDWNTVLATFRKLYPSK
TFPADFPDQGQDLSKFDTAPSMEILKSLGRPGWRSIEESIKDLVGSEMA

5. Supplementary References

1. Voss, M. *et al.* Multi-faceted Set-up of a Diverse Ketoreductase Library Enables the Synthesis of Pharmaceutically-relevant Secondary Alcohols. *ChemCatChem* **13**, 1538–1545 (2021).
2. Kille, S. *et al.* Reducing codon redundancy and screening effort of combinatorial protein libraries created by saturation mutagenesis. *ACS Synth. Biol.* **2**, 83–92 (2013).
3. Xia, Y. *et al.* T5 exonuclease-dependent assembly offers a low-cost method for efficient cloning and site-directed mutagenesis. *Nucleic Acids Res.* **47**, 1–11 (2019).
4. Miyazaki, K. & Takenouchi, M. Creating Random Mutagenesis Libraries Using PCR of Whole Plasmid. *Biotechniques* **33**, 1033–1034, 1036–1038 (2002).
5. Goldenzweig, A. *et al.* Automated Structure- and Sequence-Based Design of Proteins for High Bacterial Expression and Stability. *Mol. Cell* **63**, 337–346 (2016).
6. MacQueen, James and others. Some methods for classification and analysis of multivariate observations. *Proceedings of the fifth Berkeley Symposium on mathematical statistics and probability* **1**, 281–297 (1967).
7. van der Maaten, L. & Hinton, G. Visualizing data using t-sne. *J. Mach. Learn. Res.* **9**, 2579–2605 (2008).
8. Schlieben, N. H. *et al.* Atomic resolution structures of R-specific alcohol dehydrogenase from *Lactobacillus brevis* provide the structural bases of its substrate and cosubstrate specificity. *J. Mol. Biol.* **349**, 801–813 (2005).
9. Niefind, K., Müller, J., Riebel, B., Hummel, W. & Schomburg, D. The crystal structure of *R*-specific alcohol dehydrogenase from *Lactobacillus brevis* suggests the structural basis of its metal dependency. *J. Mol. Biol.* **327**, 317–328 (2003).
10. Noey, E. L. *et al.* Origins of stereoselectivity in evolved ketoreductases. *Proc. Natl. Acad. Sci. USA* **112**, E7065–E7072 (2015).
11. Weckbecker, A. & Hummel, W. Cloning, expression, and characterization of an (*R*)-specific alcohol dehydrogenase from *Lactobacillus kefir*. *Biocatal. Biotransformation* **24**, 380–389 (2006).
12. Hong, H. *et al.* Ketoreductase mutant and the method for producing chiral alcohol. CN110257351A (2019).
13. Perinbam, K., Balaram, H., Guru Row, T. N. & Gopal, B. Probing the influence of non-covalent contact networks identified by charge density analysis on the oxidoreductase BacC. *Protein Eng. Des. Sel.* **30**, 265–272 (2017).
14. Xu, G. C., Shang, Y. P., Yu, H. L. & Xu, J. H. Identification of key residues in *Debaryomyces hansenii* carbonyl reductase for highly productive preparation of (S)-aryl halohydrins. *Chem. Commun.* **51**, 15728–15731 (2015).
15. Nie, Y. *et al.* Purification, characterization, gene cloning, and expression of a novel alcohol dehydrogenase with anti-prelog stereospecificity from *Candida parapsilosis*. *Appl. Environ. Microbiol.* **73**, 3759–3764 (2007).
16. Yasohara, Y. *et al.* Molecular cloning and overexpression of the gene encoding an NADPH-dependent carbonyl reductase from *Candida magnoliae*, involved in stereoselective reduction of ethyl 4-chloro-3-oxobutanoate. *Biosci. Biotechnol. Biochem.* **64**, 1430–1436 (2000).
17. Xu, G., Zhang, Y., Wang, Y. & Ni, Y. Genome hunting of carbonyl reductases from *Candida glabrata* for efficient preparation of chiral secondary alcohols. *Bioresour. Technol.* **247**, 553–560 (2018).
18. Wang, S. *et al.* Application of ketoreductase in preparation of (*S*)-1, 1-di(4-fluorophenyl)-2-propanol and preparation. CN111057725A (2020).
19. Yang, Y., Drolet, M. & Kayser, M. M. The dynamic kinetic resolution of 3-oxo-4-phenyl-β-lactam by recombinant *E. coli* overexpressing yeast reductase Ara1p. *Tetrahedron Asymmetry* **16**, 2748–2753 (2005).
20. Yokochi, N., Yoshikane, Y., Trongpanich, Y., Ohnishi, K. & Yagi, T. Molecular cloning, expression, and properties of an unusual aldo-keto reductase family enzyme, pyridoxal 4-dehydrogenase, that catalyzes irreversible oxidation of pyridoxal. *J. Biol. Chem.* **279**, 37377–37384 (2004).
21. Kosjek, B. *et al.* Purification and Characterization of a Chemotolerant Alcohol Dehydrogenase Applicable to Coupled Redox Reactions. *Biotechnol. Bioeng.* **86**, 55–62 (2004).
22. Abokitse, K. & Hummel, W. Cloning, sequence analysis, and heterologous expression of the gene encoding a (*S*)-specific alcohol dehydrogenase from *Rhodococcus erythropolis* DSM 43297. *Appl. Microbiol. Biotechnol.* **62**, 380–386 (2003).
23. Wang, S. *et al.* Unconserved substrate-binding sites direct the stereoselectivity of medium-chain alcohol dehydrogenase. *Chem. Commun.* **50**, 7770–7772 (2014).
24. Vieille, C., Zeikus, J. G. & Ziegelmann-Fjeld, K. I. Molecular design of thermostable alcohol dehydrogenase for synthesis for chiral aromatic alcohols. WO2008013949A2 (2008).
25. Contente, M. L. *et al.* Stereoselective reduction of aromatic ketones by a new ketoreductase from *Pichia glucozyma. Appl. Microbiol. Biotechnol.* **100**, 193–201 (2016).
26. Petit, P. *et al.* Crystal Structure of Grape Dihydroflavonol 4-Reductase, a Key Enzyme in Flavonoid Biosynthesis. *J. Mol. Biol.* **368**, 1345–1357 (2007).
27. Paananen, P. *et al.* Structural and functional analysis of angucycline C-6 ketoreductase LanV involved in landomycin biosynthesis. *Biochemistry* **52**, 5304–5314 (2013).
28. Lanišnik Rižner, T., Moeller, G., Thole, H. H., Žakelj-Mavrič, M. & Adamski, J. A novel 17β-hydroxysteroid dehydrogenase in the fungus *Cochliobolus lunatus*: New insights into the evolution of steroid-hormone signalling. *Biochem. J.* **337**, 425–431 (1999).
29. Liao, D. I., Thompson, J. E., Fahnestock, S., Valent, B. & Jordan, D. B. A structural account of substrate and inhibitor specificity differences between two naphthol reductases. *Biochemistry* **40**, 8696–8704 (2001).
30. Kamitori, S., Iguchi, A., Ohtaki, A., Yamada, M. & Kita, K. X-ray structures of NADPH-dependent carbonyl reductase from *Sporobolomyces salmonicolor* provide insights into stereoselective reductions of carbonyl compounds. *J. Mol. Biol.* **352**, 551–558 (2005).

31. Iding, H., Reents, R., Scalone, M. & Gosselin, F. Processes for the preparation of pyrimidinylcyclopentane compounds. US20160297773A1 (2016).

32. Li, J. *et al.* Structure-Guided Directed Evolution of a Carbonyl Reductase Enables the Stereoselective Synthesis of (2S,3S)-2,2-Disubstituted-3-hydroxycyclopentanones via Desymmetric Reduction. *Org. Lett.* **22**, 3444–3448 (2020).

33. Li, H., Yang, Y., Zhu, D., Hua, L. & Kantardjieff, K. Highly enantioselective mutant carbonyl reductases created via structure-based site-saturation mutagenesis. *J. Org. Chem.* **75**, 7559–7564 (2010).

34. Li, H., Zhu, D., Hua, L. & Biehl, E. R. Enantioselective reduction of diaryl ketones catalyzed by a carbonyl reductase from *Sporobolomyces salmonicolor* and its mutant enzymes. *Adv. Synth. Catal.* **351**, 583–588 (2009).

35. Chen, X., Zhang, H., Feng, J., Wu, Q. & Zhu, D. Molecular Basis for the High Activity and Enantioselectivity of the Carbonyl Reductase from *Sporobolomyces salmonicolor* toward α-Haloacetophenones. *ACS Catal.* **8**, 3525–3531 (2018).
