# Supplementary material for: Effective engineering of a ketoreductase for the biocatalytic synthesis of an ipatasertib precursor
Source: Commun Chem. 2024 Feb 28;7:46. doi: 10.1038/s42004-024-01130-5 (PMC10902378; doi:10.1038/s42004-024-01130-5)
Supplement: Supplementary file 3 — Description of Additional Supplementary Files [file 42004_2024_1130_MOESM3_ESM.pdf]

Description of Additional Supplementary Files

File name: Supplementary Data 1

Description: Pages 1 – 2) 100 mL-scale reaction catalyzed by SsalKRED_M6 using 1a as starting material and iPrOH as reductant. Page 3) 1H NMR spectrum of of 2a. Page 4) 1H NMR spectrum of of 2a (expansion region 1). Page 5) 1H NMR spectrum of of 2a (expansion region 2). Pages 6 - 7) 1H−13C HSQC spectrum of 2a (600/150 MHz, CDCl3). Page 8) Superimposed NMR plots of 2a from two different batches.

File name: Supplementary Data 2

Description: Source Data_Figure 2

File name: Supplementary Data 3

Description: Source Data_Figure 9

File name: Supplementary Data 4

Description: Source Data_Figure 10

File name: Supplementary Data 5

Description: Source Data_Figure 11

File name: Supplementary Data 6

Description: Source Data Table 2
