# Supplementary material for: Effective engineering of a ketoreductase for the biocatalytic synthesis of an ipatasertib precursor
Source: Commun Chem. 2024 Feb 28;7:46. doi: 10.1038/s42004-024-01130-5 (PMC10902378; doi:10.1038/s42004-024-01130-5)

Structure Analytics, NMR & MS, non-GxP

NMR spectra

Structural_id_nmr	stereochemistry determination of **2a** (Batch 2021002020557)
Purity_1H_visual	>95%
Linebroadening_1H	no broad signals
Dynamics_1H	none
NMR_comment	NMR data identical with **2a** (Batch 2021002020556)
NMR_comment_2	Overlay plot shows:<br>red : Batch 2021002020557<br>blue : Batch 2021002020556

Final structure 20.10.2021

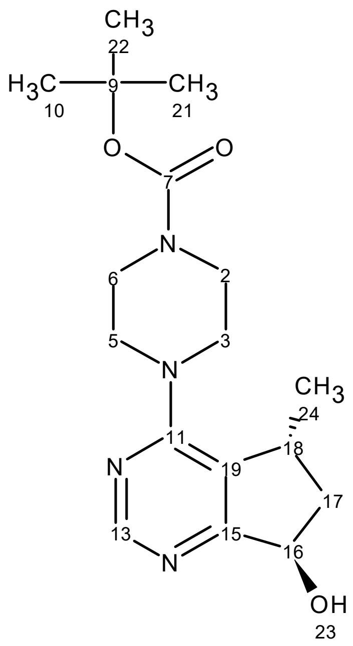

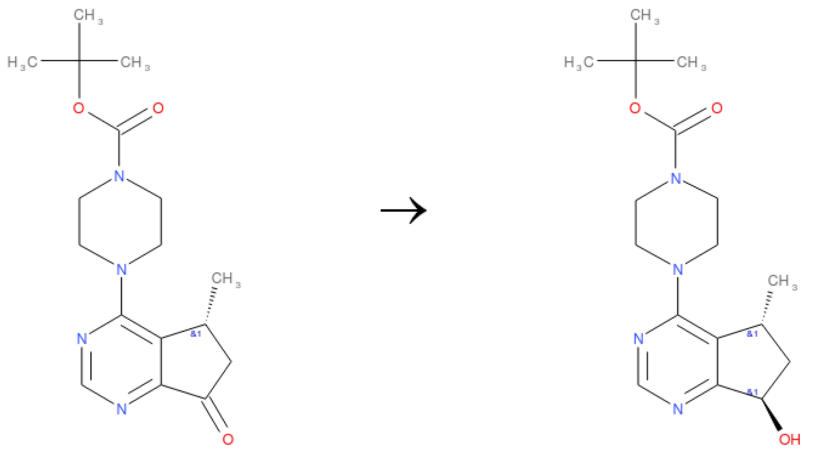

C17H24N4O3 (332.1848) --> C17H26N4O3 (334.2005)

Reactants:
4-[rac-(5R)-7-keto-5-methyl-5,6-dihydrocyclopenta[d]pyrimidin-4-yl]piperazine-1-carboxylic acid tert-butyl ester // Ssal-KRED_M6 lyo // NADP+
Solvents:
KPi 0.1 M, pH 7.2 // Isopropanol // MgCl2 * 6 H2O // water

**Method**	**Sample Preparation**	**Sample amount**	**Sample back**
NMR&MS	CDCl3	1.9 mg	N

Structure ID	Ref Order ID1	Ref Order ID2	Ref Order ID3	Ref Order ID4	Ref Order ID5
E1	2013001251714				

1H-NMR spectrum - overview

Date	18 Oct 2021 18:54:44 (GMT+02:00)
Frequency (MHz)	600.1300
Nucleus	1H
Number of Transients	8
Solvent	CHLOROFORM-d
Temperature (degree C)	25.027
File Name	NMR\nmrfs_u\data\actual\nmr\2021002020557\1000\PDATA\1\1r

Comment Sample amount 1.9 mg - Topic 7728 IPATASERTIB

^{1}H NMR (600 MHz, CHLOROFORM-*d*) δ ppm 8.54 (s, 1 H), 5.11 (t, *J*=7.2 Hz, 1 H), 3.78 (ddd, *J*=13.1, 7.3, 3.3 Hz, 2 H), 3.64 (ddd, *J*=13.0, 6.9, 3.3 Hz, 2 H), 3.46 - 3.60 (m, 5 H), 3.24 - 3.46 (m, 1 H), 2.12 - 2.23 (m, 2 H), 1.49 (s, 9 H), 1.20 (d, *J*=7.1 Hz, 3 H)

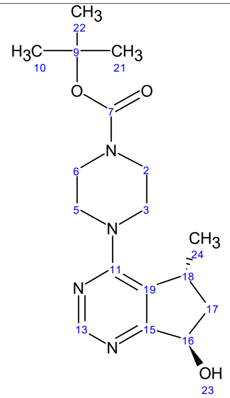

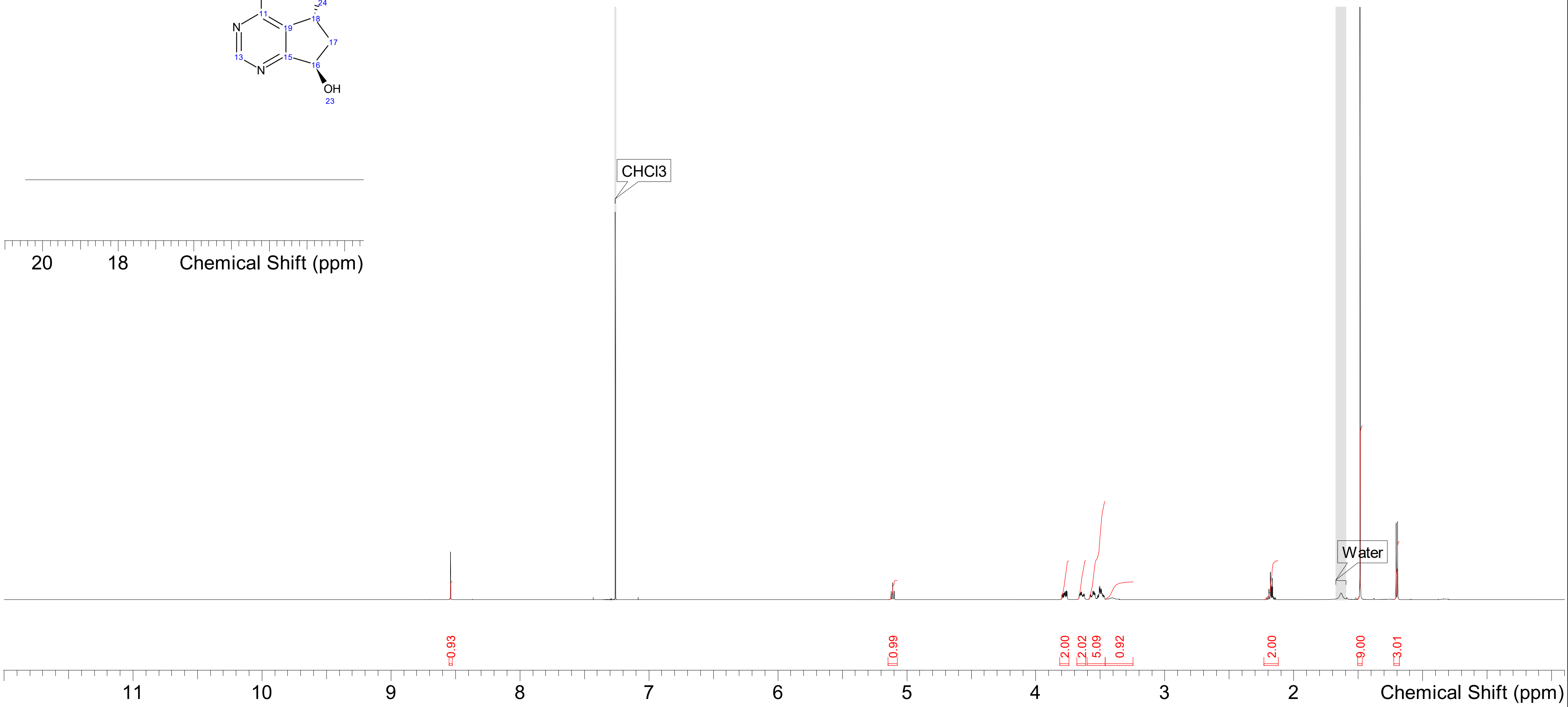

1H-NMR spectrum - expansion region 1

File Name	NMR\nmrfs_u\data\actual\nmr\20 21002020557\1000\PDATA\1\1r
Frequency (MHz)	600.1300
Nucleus	1H
Solvent	CHLOROFORM-d
Temperature (degree C)	25.027

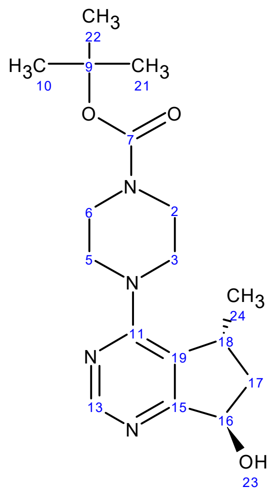

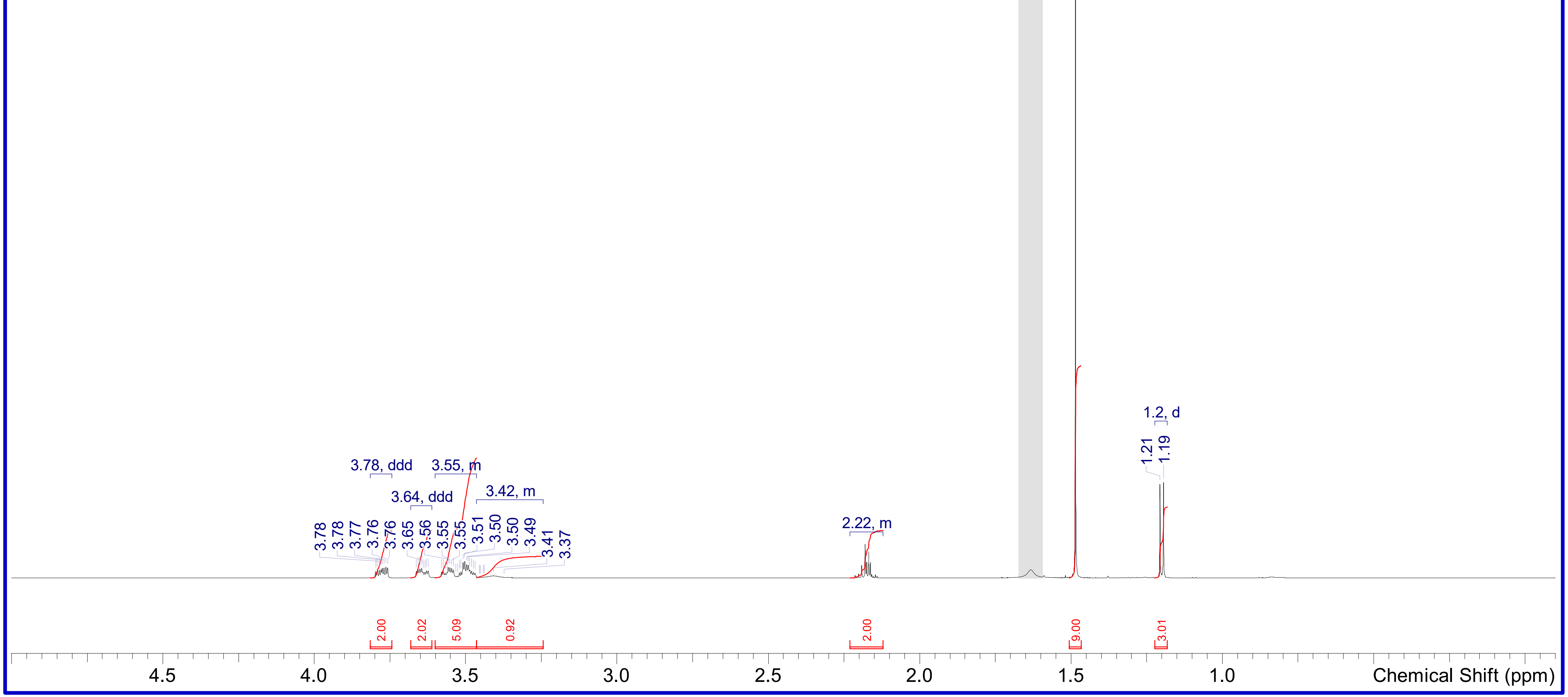

1H-NMR spectrum - expansion region 2

File Name	NMR\nmrfs_u\data\actual\nmr\20 21002020557\1000\PDATA\1\1r
Frequency (MHz)	600.1300
Nucleus	1H
Solvent	CHLOROFORM-d
Temperature (degree C)	25.027

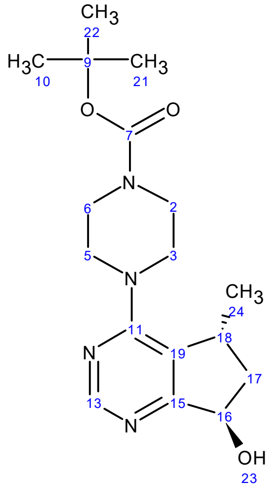

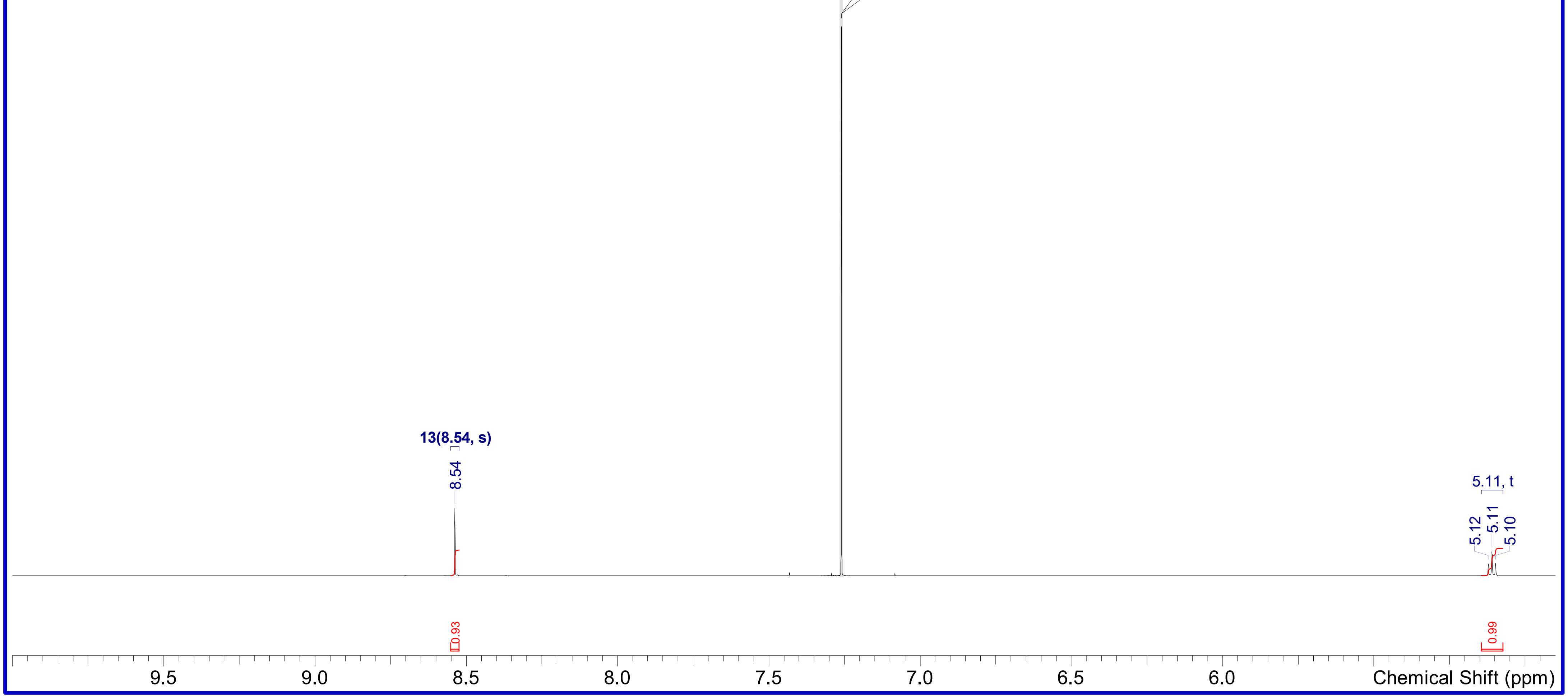

Date	18 Oct 2021 19:28:40	File Name	NMR\nmrfs_u\data\actual\nmr\2021002020557\1022\PDATA\1\2rr		
Frequency (MHz)	(600.1300, 150.9028)	Nucleus	(1H, 13C)	Solvent	CHLOROFORM-d
Spectrum Type	HSQC-DEPT				

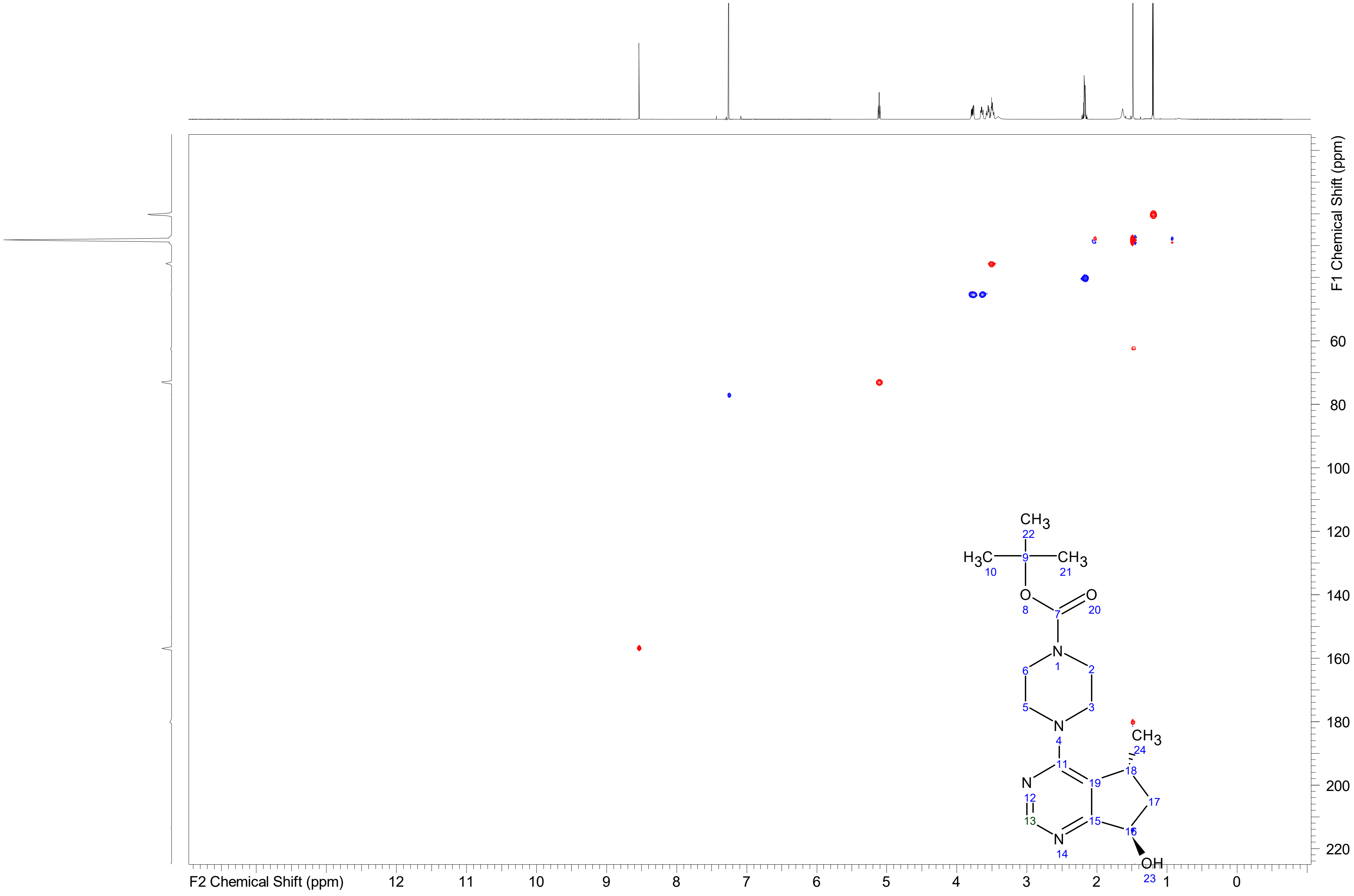

Date	18 Oct 2021 19:28:40	File Name	NMR\nmrfs_u\data\actual\nmr\2021002020557\1022\PDATA\1\2rr		
Frequency (MHz)	(600.1300, 150.9028)	Nucleus	(1H, 13C)	Solvent	CHLOROFORM-d
Spectrum Type	HSQC-DEPT				

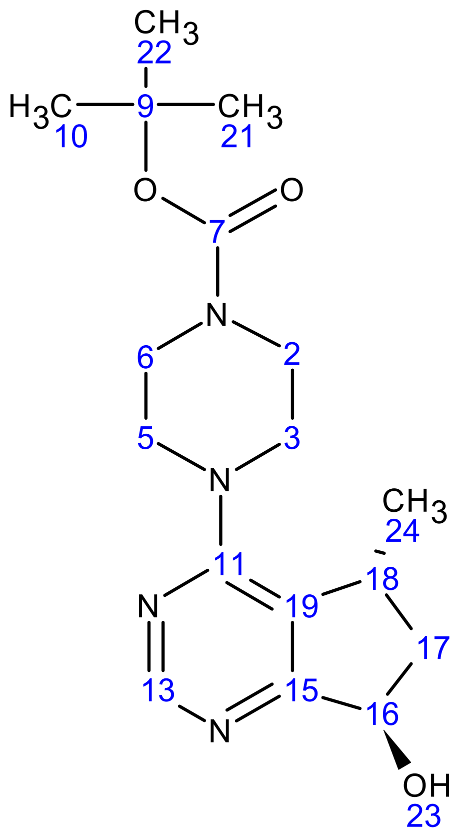

2021002020556 1000 1 Q:\data\actual\nmr

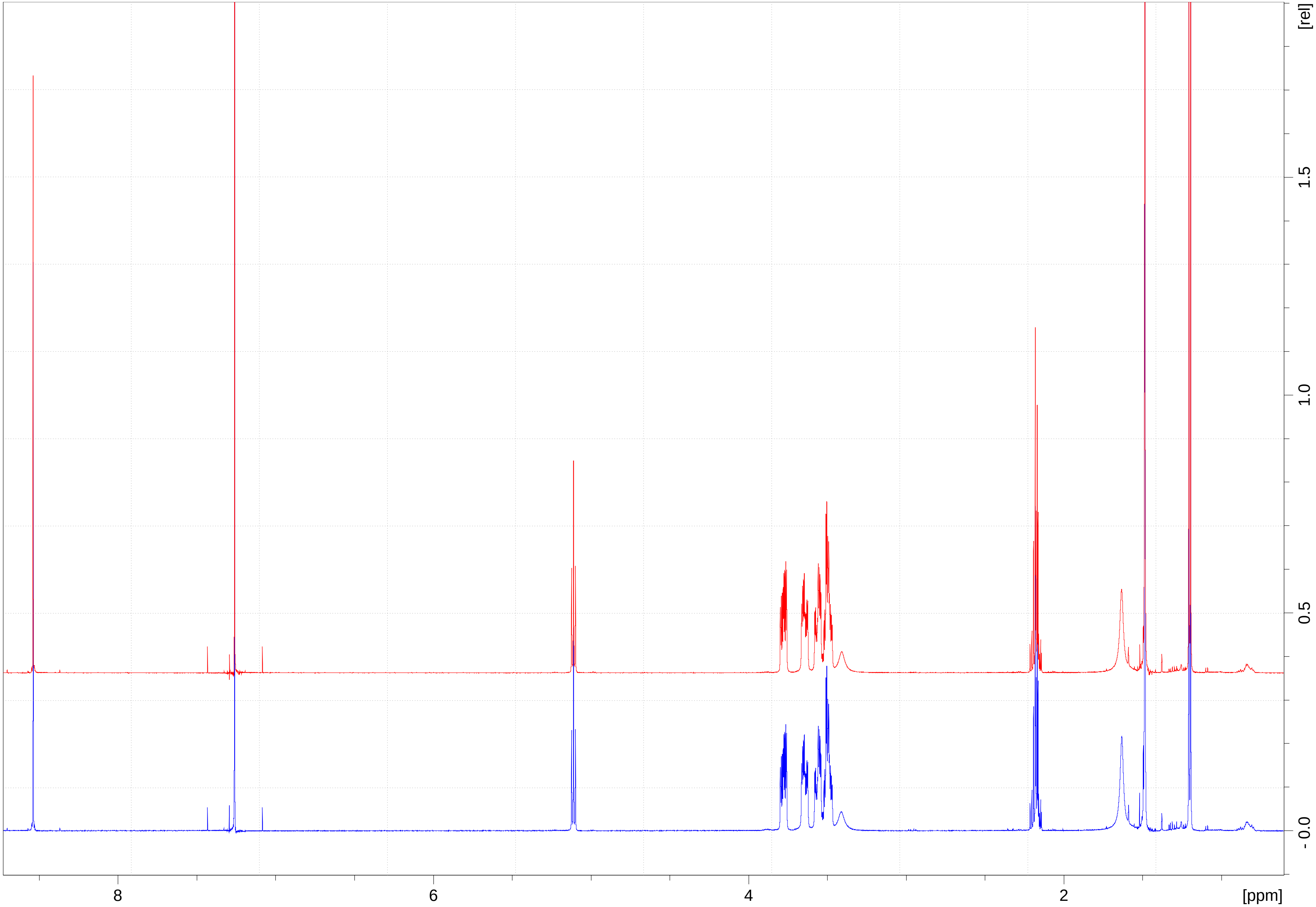

Supplement: Supplementary file 4 — Supplementary Data 1 [file 42004_2024_1130_MOESM4_ESM.pdf]
