# Supplementary material for: Effective engineering of a ketoreductase for the biocatalytic synthesis of an ipatasertib precursor
Source: Commun Chem. 2024 Feb 28;7:46. doi: 10.1038/s42004-024-01130-5 (PMC10902378; doi:10.1038/s42004-024-01130-5)
Supplement: Supplementary file 10 — Reporting Summary [file 42004_2024_1130_MOESM10_ESM.pdf]

nature portfolio

Corresponding author(s): Rebecca Buller

Last updated by author(s): February 5th, 2024

Reporting Summary

Nature Portfolio wishes to improve the reproducibility of the work that we publish. This form provides structure for consistency and transparency in reporting. For further information on Nature Portfolio policies, see our Editorial Policies and the Editorial Policy Checklist.

Statistics

For all statistical analyses, confirm that the following items are present in the figure legend, table legend, main text, or Methods section.

n/a	Confirmed	
☐	☒	The exact sample size (*n*) for each experimental group/condition, given as a discrete number and unit of measurement
☐	☒	A statement on whether measurements were taken from distinct samples or whether the same sample was measured repeatedly
☒	☐	The statistical test(s) used AND whether they are one- or two-sided<br>*Only common tests should be described solely by name; describe more complex techniques in the Methods section.*
☒	☐	A description of all covariates tested
☒	☐	A description of any assumptions or corrections, such as tests of normality and adjustment for multiple comparisons
☐	☒	A full description of the statistical parameters including central tendency (e.g. means) or other basic estimates (e.g. regression coefficient) AND variation (e.g. standard deviation) or associated estimates of uncertainty (e.g. confidence intervals)
☒	☐	For null hypothesis testing, the test statistic (e.g. *F*, *t*, *r*) with confidence intervals, effect sizes, degrees of freedom and *P* value noted<br>*Give P values as exact values whenever suitable.*
☒	☐	For Bayesian analysis, information on the choice of priors and Markov chain Monte Carlo settings
☒	☐	For hierarchical and complex designs, identification of the appropriate level for tests and full reporting of outcomes
☒	☐	Estimates of effect sizes (e.g. Cohen's *d*, Pearson's *r*), indicating how they were calculated

Our web collection on statistics for biologists contains articles on many of the points above.

Software and code

Policy information about availability of computer code

Data collection	SoftMax Pro (4.7.1) or SparkControl (2.1) were used for recording UV absorbance reads during kinetic screening assays. Agilent OpenLAB CDS (2.4, Build 2.204.0661) was utilized for the acquisition of LC-UV/MS data. NMR spectra were recorded on a Bruker Avance III 600 MHz spectrometer using TopSpin (4.x) as software.
Data analysis	Agilent OpenLAB CDS was utilized for chromatogram peak integration. Initial reaction rates sequencing alignments were handled using Microsoft Excel (Version 2102, Build 13801.21004). GraphPad Prism (9.2.0) was used for non-linear curve-fitting. ACD/NMR Workbook 2019 was utilized for the analysis of NMR data. Microsoft Excel, BioRender, Adobe Illustrator 24.1.1, ChemDraw 21.0, PyMOL 2.5.x, Plotly (5.x) Python (3.8.x) were employed for illustration purposes. MOE (2019.01) and Moloc were used for enzyme modelling. Machine learning scripts for the prediction of enzyme function are provided in the link: https://github.com/ccbiozhaw/Ssal-KRED_evolution.

For manuscripts utilizing custom algorithms or software that are central to the research but not yet described in published literature, software must be made available to editors and reviewers. We strongly encourage code deposition in a community repository (e.g. GitHub). See the Nature Portfolio guidelines for submitting code & software for further information.

Data

Policy information about availability of data

All manuscripts must include a data availability statement. This statement should provide the following information, where applicable:

- Accession codes, unique identifiers, or web links for publicly available datasets
- A description of any restrictions on data availability
- For clinical datasets or third party data, please ensure that the statement adheres to our policy

Source data are provided with this paper. NMR spectra can be found in Supplementary Data 1. The crystal structure used for the substrate docking and homology modelling experiments can be accessed via PDB ID: 1Y1P.

Human research participants

Policy information about studies involving human research participants and Sex and Gender in Research.

Reporting on sex and gender	There were no human research participants involved in this study.
Population characteristics	There were no human research participants involved in this study.
Recruitment	There were no human research participants involved in this study.
Ethics oversight	There were no human research participants involved in this study.

Note that full information on the approval of the study protocol must also be provided in the manuscript.

Field-specific reporting

Please select the one below that is the best fit for your research. If you are not sure, read the appropriate sections before making your selection.

☒ Life sciences ☐ Behavioural & social sciences ☐ Ecological, evolutionary & environmental sciences

For a reference copy of the document with all sections, see nature.com/documents/nr-reporting-summary-flat.pdf

Life sciences study design

All studies must disclose on these points even when the disclosure is negative.

Sample size	No sample size calculation was performed to predetermine sample size. Unless otherwise specified, 2 - 4 biological replicates (i.e., 2 - 4 clones containing the same plasmid construct) were used in in vitro experiments to verify improved enzyme variants after screening. The activity of improved enzyme variants was verified by Michaelis-Menten kinetics using at least 3 technical replicates. The number of samples is indicated either in the title or in the description of each table or figure.
Data exclusions	No data were excluded from the analyses.
Replication	All reported attempts at replication were successful as improved enzyme variants were utilized as positive controls in successive evolution rounds (in 3 or more independent experiments). Averaged fold-improvement-over-the-parent calculations based on initial reaction rates or averaged conversion levels are provided. When applicable, standard deviations are indicated as values or as error bars.
Randomization	Randomization was not applicable for the in vitro experiments performed in this study. However, for the prediction of improved enzyme function machine learning was conducted by splitting the data randomly into ten subsets, and each one of them was used for validation. The random seed was fixed to ensure reproducibility, e.g., for the splitting and the training procedures.
Blinding	Blinding was not necessary since this study does not involve animal or human subjects or group allocations.

Reporting for specific materials, systems and methods

We require information from authors about some types of materials, experimental systems and methods used in many studies. Here, indicate whether each material, system or method listed is relevant to your study. If you are not sure if a list item applies to your research, read the appropriate section before selecting a response.

Materials & experimental systems

n/a	Involved in the study
☒	☐ Antibodies
☒	☐ Eukaryotic cell lines
☒	☐ Palaeontology and archaeology
☒	☐ Animals and other organisms
☒	☐ Clinical data
☒	☐ Dual use research of concern

Methods

n/a	Involved in the study
☒	☐ ChIP-seq
☒	☐ Flow cytometry
☒	☐ MRI-based neuroimaging
